# Differential Proteomics Analysis of the Subcutaneous Connective Tissues in Alcian Blue Tracks along Conception Vessel and Adjacent Nonmeridian in Rats

**DOI:** 10.1155/2021/5550694

**Published:** 2021-05-04

**Authors:** Xiaojing Song, Weibo Zhang, Shuyong Jia, Shuyou Wang, Guangjun Wang, Feng Xiong, Hongyan Li

**Affiliations:** Department of Biomedical Engineering, Institute of Acupuncture & Moxibustion, China Academy of Chinese Medical Sciences, 16 Dongzhimen Nei Nanxiao Road, Dongcheng District, Beijing 100700, China

## Abstract

In more than half a century, exploring the biological connotation of the meridians was one of the core components of scientific research studies in traditional Chinese medicine (TCM). Based on the previous works of low hydraulic resistance channel (LHRC) along meridians (LHRCM), the differential proteomics between the Alcian blue track (ABT) on LHRC along the conception vessel (CV) and nonmeridians tissue next to the CV were investigated in this study to explore the material basis and biological function of LHRCM. Proteomics based on LC-MS was introduced into the subcutaneous connective tissues (SCT) of ABT along the CV and the adjacent nonmeridian (1 cm from the CV). A total of 2328 proteins were identified from ABT along the CV and adjacent nonmeridian based on data-dependent acquisition (DDA) mode. In total, 1970 proteins were quantified based on the SWATH (sequential window acquisition of all theoretical fragment ions) label-free model, and the nonstandard and quantitative methods of MSALL were applied to analyze the data. There were 468 proteins differentially expressed. GO analytic results showed that the differential proteins were of varieties in molecular function and biological process. Most of differential proteins were involved in metabolic process, cellular process, response to hormone, and response to wounding. Further analysis showed that the upregulated differential proteins involved in ATP metabolism (ATP5E, GAPDH), redox reactions (Gpx-3), and Ca^2+^ transmembrane transport (CACNA2D1) were closely related to meridian phenomenon and acupuncture effect. These differential proteins would be potential characteristic proteins of the LHRC along the CV in rats which may be useful to deepen the knowledge on LHRCM.

## 1. Introduction

For more than half a century, exploring the biological connotation of meridians has been an attractive topic. With the help of modern methods such as biology, physics, and imaging, scholars worldwide have carried out much research to observe the structural characteristics and material basis of meridians [1, 2]. Some scholars have carried out research in the field of specific protein and meridian material bases. Zhou proposed one point of view that there are proteins related to meridians in the cell membrane of the tissues along the meridians. Under certain conditions, these proteins can be sequentially altered, rearranged, and coupled with each other, resulting in complex resonance. The protein molecules along the meridian form an energy band structure with the functions of energy, information transmission, and conversion [3]. Feng considered that fibrin in meridians and acupoints is involved in the generation and transmission of acupuncture effects and that fibrin in the body is an important part of meridian essence [4]. Through a systematic study on the biological characteristics of the sensory nerve-specific protein 29-kD protein, Meng et al.[5] proposed that the traditional Chinese medicine (TCM) theory of “no obstruction no pain and vice versa,” qi sensation along meridians, acupuncture stimulation, and amplification effect of acupoint injection are all used to transmit stimulation information through the bridging of the 29-kD protein. A common differential protein band in the fat belt is observed along the stomach meridian and conception vessel (CV) but not in the fat belt beside the meridian [6]. In addition, it was found that the expression of connexin 43 was significantly higher in the plantar part of the kidney and the dorsal part of the bladder than in the adjacent nonmeridian control line [7]. These results suggest that there is a certain type of protein in meridian tissues, which may be one of the material bases of meridians.

At present, the research on the protein specificity along meridian tissues is just beginning. A few reported results are mainly the analysis based on the known results of meridian research and modern life science knowledge. Although it is scientific, the additional experimental evidence is absent. For the specific proteins found in the tissues of meridians, the identification involved in the functions of promoting blood and qi, connections between viscera, and the transmission of acupuncture signals is still unknown. Therefore, it is possible to find a new material, energy, and information transmission system in the human body, which will help clarify the essence of meridians and the mechanism of acupuncture and moxibustion.

Zhang proposed that the meridians are the low hydraulic resistance channels (LHRC) distributed in the body's interstitium. The hypothesis was verified by tracing the LHRC along the meridians (LHRCM) [8], observing the microstructure in vivo [9] and blocking the LHRCM [10]. Recently, the LHRC along the CV in rats was shown by injecting Alcian blue (AB) [11]. In this paper, the differential protein expression of the subcutaneous connective tissues between the AB tracks (ABT) on LHRC along the CV and the adjacent nonmeridian area were analyzed for the first time, to explore the material basis and biological function of the meridians. The results will provide new evidence to understand LHRCM and may be helpful to reveal the biological connotation of meridians.

## 2. Materials and Methods

### 2.1. Animals

In total, 15 healthy adult male rats with an average age of 11 weeks and a weight of 380-420 g were used in this study. All animals were provided by Biotechnology Co., Ltd. (SCXK (Beijing) 2016-0002). They were treated in accordance with international standards for the care and use of laboratory animals, and the entire experiment was performed in accordance with the protocol approved by the Animal Ethics Committee of the Institute of Acupuncture and Moxibustion at the China Academy of Chinese Medical Science.

### 2.2. The Extraction of Connective Tissue in ABT along CV and Adjacent Nonmeridian

**Figure 1:**
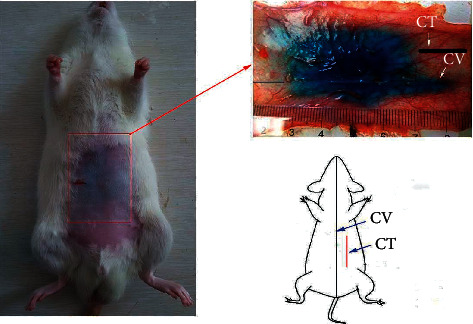
The location of the tissue sample in rats. The position of the ventral midline was considered the CV path. CV indicated the location of the tissue sample that is the SCT of the ABTs. CT indicated the location of adjacent nonmeridian tissue.

After anesthesia by 10% urethane, the subcutaneous connective tissues (2 mm wide and 1 mm thick) of the ABT along the CV and adjacent nonmeridian (the control tissue, CT, along the line where the median line of the abdomen is 1 cm horizontally) in the abdominal skin of the rats were cut (Figure 1). The tissues in the same group were divided into 5 parts, approximately 30 mg each, washed with 4°C PBS, and were kept at -80°C.

### 2.3. Extraction and Quantification of Total Protein

About 0.1 g of sample tissue was cut into pieces and added to cold protein lysate (300 *μ*L/100 g) for homogenization. The mixture was transferred to an EP tube (4°C) and shocked and lysed at 4°C and 120 rpm for 20 min. It was centrifuged at 20000 × g and 4°C for 10 min in a high-speed centrifuge to obtain the clear supernatant, which was the total protein solution of the subcutaneous connective tissues of CV and CT. The total protein concentration in the supernatant was quantified by Bradford assays (Thermo Pierce™ Rapid Gold BCA Protein Assay Kit, Thermo Scientific™, USA).

### 2.4. Protein Precipitation

Then, 20% trichloroacetic acid was added to 110 *μ*g of protein, which was extracted from each sample to a final concentration of 10%. After incubation at −80°C for 2 h, the protein was precipitated as much as possible. It was centrifuged at 14000 × g at 4°C for 20 min. Acetone (4°C) was added to the precipitate, and the solution was precipitated at −80°C overnight after ultrasonic suspension and then centrifuged at 14000 × g at 4°C for 20 min. The supernatant was discarded, and the acetone was volatilized at room temperature.

### 2.5. Protein Sample Pretreatment and Peptide Solution Preparation

Each sample was pretreated, and a peptide solution was prepared according to the following methods. The sample was added to 70 *μ*L of ammonium bicarbonate solution (50 mM) with 0.2% Rapigest SF for ultrasonic dissolving protein. The residual acetone volatilized at 37°C and 300 rpm for 10 min, and then 8 *μ*L of ammonium bicarbonate solution (50 mM) with Tris (2-carboxyethyl) phosphine (100 mM) and 2 *μ*L of ammonium bicarbonate solution (50 mM) were added. The mixture was evenly mixed for reaction at 60°C for 30 min. After cooling to room temperature, 9 *μ*L of ammonium bicarbonate solution (50 mM) with 100 mM iodoacetamide and 1 *μ*L of ammonium bicarbonate solution (50 mM) were added, and the mixture was evenly mixed for a photoprotective reaction for 30 min. Then, 5 *μ*L of restricted trypsin (0.4 *μ*g/*μ*L) was added and digested overnight at 37°C and 140 rpm. Then, 5 *μ*L of trifluoroacetic acid (10%) was added for termination digestion at 37°C and 140 rpm for 30 min. The supernatant containing the digested peptide was transferred to a 10 kDa ultrafiltration membrane and centrifuged at 14000 r/min for 15 min. The filtrate (1 *μ*g/*μ*L) was preserved at −80°C.

### 2.6. DDA Mass Spectrometry

The data of mixed samples were acquired based on a data-dependent acquisition (DDA) model by the AB Sciex5600 + TripleTOF platform. The peptide solutions of CV (5 *μ*L) and CT (5 *μ*L) samples were mixed. Mixed sample preconcentration was performed by nanoliquid chromatography and nanoliter-high-performance liquid chromatography elution. A capillary direct injection chromatographic column was used for sample separation and analysis, and the parameters of gradient elution were as follows (Table 1).

Phase A: methane-acetonitrile-water (0.1 : 2:98, v/v/v); Phase B: methane-acetonitrile-water (0.1 : 98 : 2, v/v/v).

The parameters of TOS MS were as follows: nano electrospray ionization; nebulizing gas (GS1), 10 kPa; CUR, 30 kPa; ISDF, 2500 V; IHF, 150; DP, 100 V; MS/MS CE, 10 eV; time, 250 ms; mass number deviation, 50 mDa; dynamic exclusion acquisition time, 15 s. The first-order spectra of the samples were scanned in the range of 350-650 m/*z* and 645-1250 m/*z* by online classification method. The ion level 2 spectra of fragments were collected in high sensitivity mode, MS2 (m/*z*), 100～1500; one cycle acquisition time, 100 ms. The mass spectrometry data of CT-F1, CT-F2, CV-F1, CV-F2, Mix-F1, and Mix-F2 were obtained.

### 2.7. Qualitative Identification of Protein

The FASTA files of rat proteins were downloaded from NCBI and imported into the temp of ProteinPilot.

The protein identification method was established to search for results from the identification protein library.

### 2.8. SWATH Analysis

The sequential window acquisition of all theoretical fragment ions (SWATH) method was established. The mixed peptide data were collected in the range of 350-1250 M/*Z*. The peptide sample data were collected by nano-LC and SWATH MSALL. There were 5 biological duplications in the CV group and 4 biological duplications in the CT group. Each sample was collected 3 times.

### 2.9. Data Processing and Biological Information Analysis

The SWATH data were analyzed by the Peak View SWATH Processing Micro App. The proteins that can be quantified by SWATH were extracted from the identification protein library, and the corresponding peptide and fragment ion information were extracted. The confidence interval was 95%, and the false discovery rate (FDR) was 1.0%. The relative quantitative comparison was made between the CV group and the CT group by Markervie. Differential expression of proteins between the CV group and CT group was selected by *t*-test, *P* < 0.05, and CV/CT ≥ 1.5 or ≤0.067. The total protein and differential protein data were analyzed by GO, Kyoto Encyclopedia of Genes and Genomes (KEGG), protein-protein interaction (PPI), and BP link KEGG. The functions of differential proteins and their biological processes were analyzed by consulting the UniProt database. Then, according to natural studies of TCM meridians, the relationship between differential proteins and TCM meridians was analyzed, and four or five proteins that are closely related to the function of meridians were identified.

### 2.10. Western Blot Analysis

The supernatant (4 *μ*g/*μ*L) mixed with a certain proportion of 5 ×  and 1 ×  SDS-PAGE loading buffer (Solarbio, China) was boiled at 95°C for 10 min. Equal amounts of protein (50 *μ*g/20 *μ*L) were separated by 10% SDS-PAGE (SDS-PAGE Gel Kit, Solarbio Life Sciences, China). Proteins were electrophoretically transferred to polyvinylidene difluoride membranes (Bio-Rad, Richmond, USA), which were then blotted with an antibody against ATP synthase subunit epsilon (ATP5E) (ThermoFisher, USA) diluted 1 : 500, voltage-dependent calcium channel subunit alpha-2/delta-1 (CACNA2D1) (Abcam, Hong Kong) diluted 1 : 10000, glutathione peroxidase 3 (Gpx-3) (Santa, USA) diluted 1 : 2000, and glyceraldehyde-3-phosphate dehydrogenase (GAPDH) (Immunoway, USA) diluted 1 : 20000. A secondary antibody (Abcam, Hong Kong) was used to detect the primary antibody. Anti-beta actin antibody (Immunoway, USA) and secondary antibody (Abcam, Hong Kong) were used as references. Finally, chemiluminescence analysis, development, and fixation were carried out. Band signals were detected using an iBright CL750 western blot imaging system (ThermoFisher, USA) and analyzed using iBright Software (ThermoFisher, USA).

### 2.11. Statistical Analysis

SPSS 19.0 statistical software was used for statistical analysis. One-way ANOVA was used to compare differences in the same index among the groups, and the LSD test was performed to determine significant differences between two groups. The data are expressed as the mean ± SD, with *P* < 0.05 indicating statistical significance.

## 3. Results

### 3.1. Proteomic Identification of ABT along CV and CT

**Figure 2:**
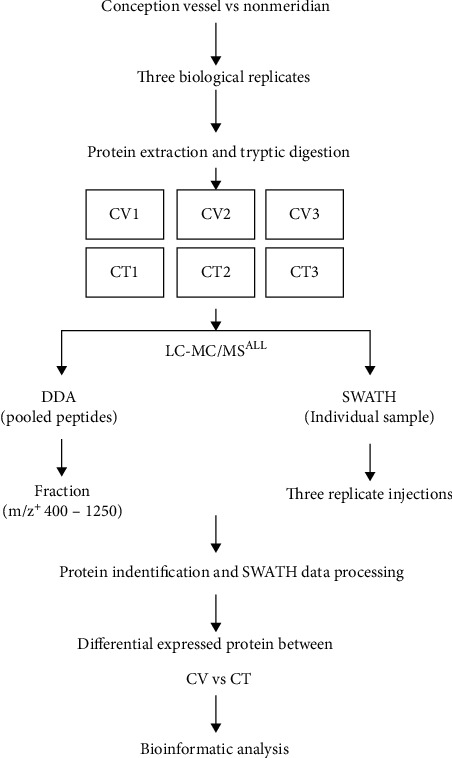
Scheme for the subcutaneous connective tissues in ABT along CV and CT proteomics. CV was the SCT of the ABT along the conception vessel. CT was the SCT of the adjacent nonmeridian.

**Figure 3:**
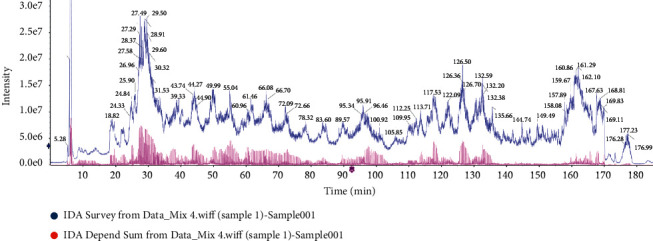
Mass spectrogram of total ions in sample IDA-dependent sum mode from Data Mix 4.

The subcutaneous connective tissues of ABT along CV and CT in three rats of the same body were chosen as experimental specimen. The pooled and tryptic digested protein samples were analyzed by tandem LC-MS in data-dependent acquisition (DDA) model, and the acquired data were processed by ParagonTM (AB Sciex) (Figure 2). ProteinPilot identified 169161 secondary spectra with 1% confidence level, 15196 peptide fragments within the global FDR 63.7% and 1% confidence level, and 2328 proteins within the global FDR 97.6% and 1% confidence level (Figure 3).

### 3.2. Differential Proteomics Analysis of ABT along CV and CT

**Figure 4:**
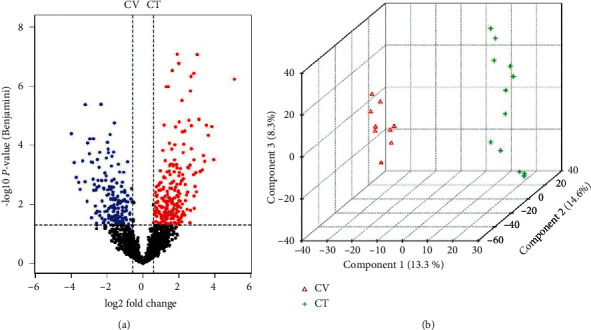
Proteome volcano map of differential proteins and PCA score plots of proteome data between the CV and CT. Note: (a) CV vs CT *t*-test result log (fold change) versus *P* value for A to B. (b) PCA plots compared between CV and CT. CV was the SCT of the ABT along the conception vessel. CT was the SCT of the adjacent nonmeridian.

There is a good linear relationship between the DDA database and SWATH data. In total, 1988 proteins were quantified from 2328 proteins. Among them, 1970 were effectively quantified. The peak areas of all quantified proteins were normalized. CT3 and CV1 were eliminated because there was no significant difference between CT3 and CV1 in principal component analysis (Figure 4(a)). The remaining groups were subjected to PAC and *t*-test analysis, and 468 proteins were found to be differentially expressed (Figure 4(b)).

**Figure 5:**
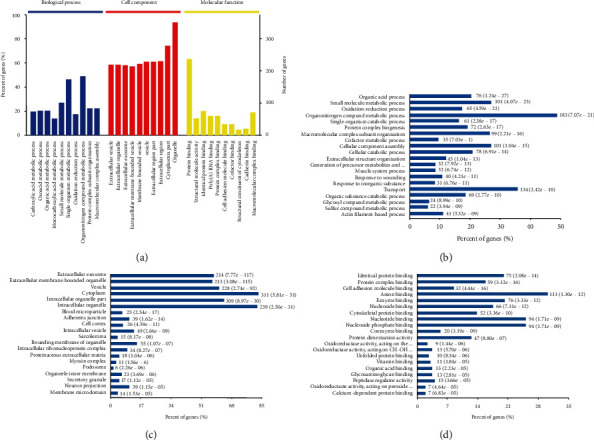
GO analysis of differential proteins between the SCT of the ABT along the CV and adjacent nonmeridian tissue. (a) GO annotations of all quantified proteins. (b) GO-cell component, CC. (c) GO-molecular function, FC. (d) GO-biological process, BP.

GO analysis showed that the differential proteins were distributed in the cell membrane or the structure of organelle intima, such as extracellular membrane-bound organelles, adhesion junction, organelle binding membrane, organelle outer membrane, and organelle inner membrane; in the extracellular matrix; and in the structure of cell membrane or organelle intima, such as extracellular membrane-bound organelles, adhesion junction membrane, organelle outer membrane, organelle inner membrane, endoplasmic reticulum, membrane microstructure domain, and muscle fiber membrane. The differential proteins were also found to be located in the cytoplasm, tricarboxylate cyclase complex, blood cell microparticles, projection neurons, and glial cell projections (Figures 5(a) and 5(b)). The molecular functions of the differential proteins may be isoprotein binding, coenzyme binding, enzyme binding, peptide binding, nucleoside binding, nucleotide binding, cell adhesion molecule binding, protein complex binding, S100 protein binding, vitamin binding, anion binding, oxidoreductase activation, intramolecular oxidoreductase activation, nucleoactivation of carbon oxygen lyase, activation of protein dimers, activation of peptidase regulators, and activation of glutathione oxide enzymes (Figure 5(c)). Most of the differentially expressed proteins were involved in small molecule metabolism, organic acid, single-organism catabolism, oxidation-reduction, organonitrogen compound, cellular catabolism, cofactor metabolism, organic substance catabolism, inorganic response, sulfur compound metabolism, organic response, cellular lipid metabolism, single-organism biosynthesis, hormone response, cellular aldehyde metabolism, lipid metabolism, organophosphate metabolism, aging, wounding response, and the tricarboxylic acid cycle (Figure 5(d)).

Based on previous studies on meridians, ATP metabolism-related proteins are differentially expressed between the ABT along CV tissues and adjacent nonmeridian tissues in rats. Among them, the expression of proteins involved in glycolysis was upregulated. Glyceraldehyde-3-phosphate dehydrogenase (GADPH, P04797) is a key enzyme in glycolysis. It is involved in the first step of catalyzing the biochemical reaction and producing a large amount of ATP. The differential expression of Gxp-3 suggested that the glycolytic metabolism in the ABT along the CV was more active than that in adjacent nonmeridian tissues.

At the same time, ATP synthase, one of the key substances involved in the oxidative phosphorylation process, was differentially expressed between the subcutaneous connective tissues of ABT along the CV and nonmeridian tissue in rats, such as ATP synthase subunit epsilon, mitochondria (P29418), ATP synthase subunit *d*, mitochondria (P31399), ATP synthase-coupling factor 6, mitochondrial (P21571). ATP synthase subunits are distributed in the inner membrane of mitochondria. During the process of oxidative phosphorylation, they participate in the biological process of ATPase activation to directly affect ATP synthesis. The differential expression of ATP synthase suggested that the process of oxidative phosphorylation is more active in the subcutaneous connective tissues of ABT along the CV than in that of adjacent nonmeridian tissues and that the efficiency of ATP production was increased.

Proteins involved in the redox reaction process were differentially expressed between the subcutaneous connective tissues of ABT along the CV and nonmeridian tissue in rats. For example, glutathione peroxidase 3 (Gpx-3, P23764) is involved in glutathione metabolism, the hydrogen peroxide catabolism process, and the response to oxidative stress. The differential expression of Gpx-3 suggested that the reactive oxygen species scavenging occurs along the CV.

The functional proteins related to Ca^2+^ transmembrane transport were differentially expressed between the subcutaneous connective tissues of the ABT along the CV and nonmeridian tissue in rats. These differentially expressed proteins mainly included the voltage-dependent L-type calcium channel protein family, such as voltage-dependent L-type calcium channel subunit beta-1 (P54283) and voltage-dependent calcium channel subunit alpha-2/delta-1 (CACNA2D1, P54290). CACNA2D1 can regulate physical properties to affect the calcium current density and activation/deactivation of calcium channels. It plays an important role in excitation contraction coupling and excitation secretion coupling. Ca^2+^ is an important substance for information transmission and sensation transmission along meridians after acupuncture stimulation. The upregulated expression of CACNA2D1 suggested that the transmembrane transport of Ca^2+^ occurs along the CV.

### 3.3. KEGG Analysis

**Figure 6:**
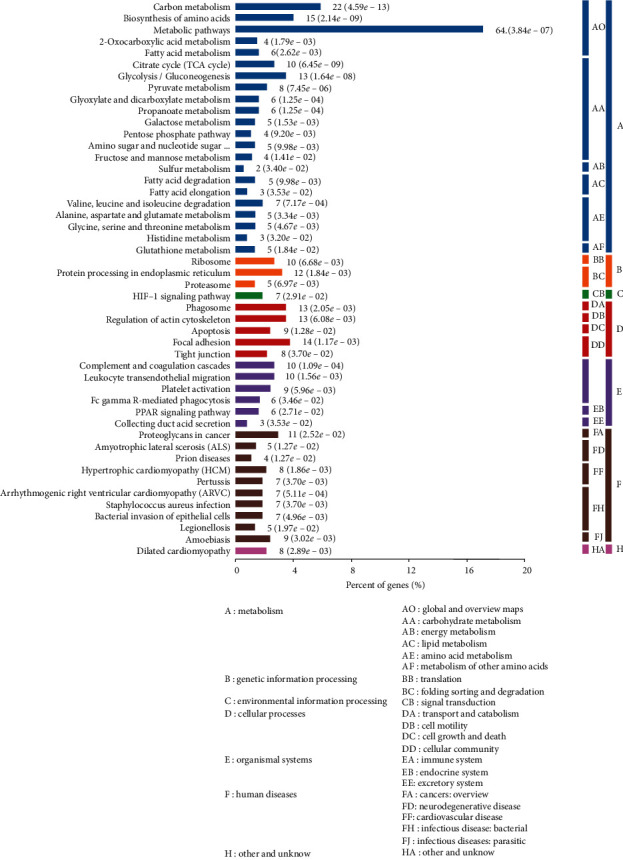
KEGG analysis of differential expressed proteins between the SCT of the ABT along the CV and adjacent nonmeridian tissue.

Differential proteins were involved in the following metabolic pathways: metabolism, genetic information processing, environmental information processing, cellular processes, organic systems, and disease formation. Most proteins (207) were involved in metabolic processes, mainly carbon metabolism (22), amino acid biosynthesis (15), metabolic pathways (64), carbohydrate metabolism (61), glucose metabolism (18), glycolysis (13), amino acid metabolism (20), fat metabolism (8), and glutathione metabolism (5). Some of the differentially expressed proteins were involved in genetic information processing, cellular processes, organismal systems, and human diseases (Figure 6). The GO analysis results indicate that the differential proteins were mainly involved in material and energy metabolism.

### 3.4. Differential Protein Expression

**Figure 7:**
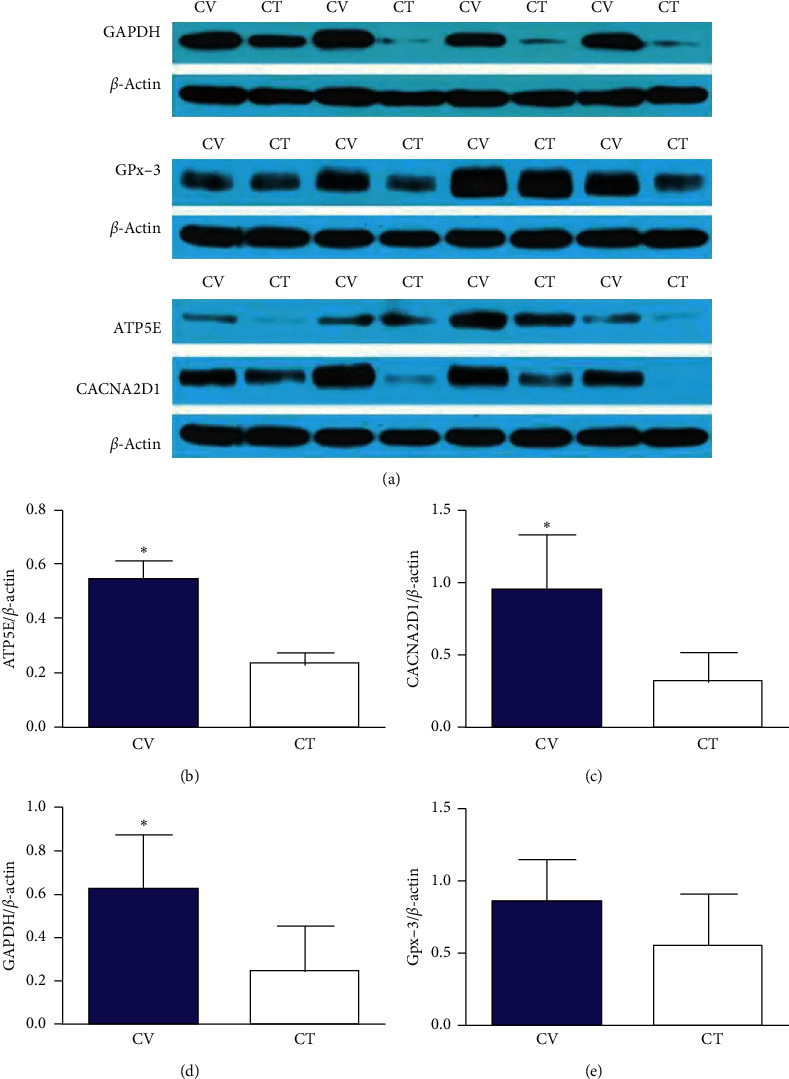
Expression of differentially expressed proteins between the SCT of the ABT along the CV and adjacent nonmeridian tissue. Note: ^*∗*^*P* < 0.05 vs CT (LSD two-group comparison test). The data are shown as the mean ± SD.

Western blot results indicated that ATP5E, CACNA2D1, and GAPDH expression was significantly higher in the ABT along the CV than in nonmeridian tissue (*P*=0.01 < 0.05 (Figures 7(a) and 7(b)), *P*=0.013 < 0.05 (Figures 7(a) and 7(c)), *P*=0.002 < 0.05 (Figures 7(a) and 7(d))). Gpx-3 expression in the ABT along the CV was also higher than that in nonmeridian tissue (*P*=0.08) (Figures 7(a) and 7(e)).

## 4. Discussion

The results showed that the differentially expressed proteins were mainly distributed in organelles such as vesicles, secretory vesicles, and the structure of the cell membrane or inner membrane of organelles. They are involved in the transportation, identification, and transfer of transmitters, cytokines, and ions. The molecular functions of the differentially expressed proteins were mainly the binding of proteins, nucleosides, enzymes, mucopolysaccharides, and ions. The biological processes associated with the differential proteins were the material metabolism pathway, response to hormones, and response to mechanical injury. Based on the meridian phenomena and acupuncture effects, we proposed that the expression levels of GAPDH, CACNA2D1, ATP5E, and Gpx-3 were upregulated in the ABT along the SCT of CV compared with nonmeridian tissues in rats. These proteins are involved in ATP metabolism, redox reactions, and calcium ion transmembrane transport which are related to the function of meridians.

Meridians in TCM are the channels for running qi and blood. They can connect between viscera and body surface and between different parts of body surface to form a regulatory system on human body. All material transportation and information transmission must consume energy. Therefore, it is inferred that the energy metabolism of meridians tissues should be high. In fact, it was higher infrared radiation [12], ultramicro luminescence, increased oxygen consumption [13], carbon dioxide release [14], and blood perfusion along meridians compared with the adjacent nonmeridian regions [15] which indicated an active energy metabolism along the meridians. In addition, higher nerve ending density, higher K+, Na+, and Ca^2+^ concentrations, and aggregated mast cells were observed in the tissues along meridians [16]. It is conducive to an active biological process along the meridians. Of course, a large amount of ATP is involved.

ATP is an important high-energy phosphate compound which is the main energy form used by cells directly. ATP synthase is the key substance in the process of catalyzing ATP production in the process of oxidative phosphorylation, which is involved in the activation of ATPase and ATP synthesis [17–20]. In this study, ATP5E, one of the key substances involved in the process of oxidative phosphorylation, was differentially expressed between ABT along the CV and adjacent nonmeridian tissue. This result indicated that the oxidative phosphorylation process in the subcutaneous connective tissues along the CV was more active and the efficiency of ATP production was increased, providing an evidence for the meridian characteristics of high energy metabolism.

However, when the body is in a state of stress, hypoxia, or strenuous exercise, energy is mainly provided by glycolysis. After acupuncture and moxibustion stimulation, tissues along the meridian path are stressed, and the energy metabolism is mainly provided by glycolysis. The class of cells with extremely active metabolism, such as nerve cells and white blood cells, also need some of the energy provided by glycolysis, even in the absence of hypoxia [21]. There are abundant nerve endings, neurons, and immune cells in the tissues of acupoints and meridian paths. A large amount of ATP is required for maintaining physiological metabolism of these cells. Therefore, glycolysis in tissues along the meridian path is active. GAPDH is the key enzyme during glycolysis and is involved in the 1-step reaction. The quantity and activity of GAPDH directly affect ATP synthesis [22, 23]. In this study, GAPDH expression was upregulated in the ABT along the CV, indicating that glycolysis was more active in the SCT along the CV than that in nonmeridian tissues. GAPDH provides energy for metabolic activities and information transmission along meridians which is important for the transmission of energy along meridians.

ATP production in eukaryotic cells mainly occurs in mitochondria with the formation of mitochondrial respiration chains (MRCs) and superoxide anions. Superoxide anions are reduced in mitochondria to produce strong oxidizing reactive oxygen species, H_2_O_2_ and hydroxyl radicals (·OH). The chemical properties of these reactive oxygen species are very active which can cause oxidative damage to proteins, DNA, and other macromolecules. Nevertheless, the body can remove active oxygen species in time via endogenous antioxidant enzymes [24]. GPX is an antioxidant enzyme that widely exists in the body and Gpx-3 is one of the main members in GPX family. As the energy metabolism of the tissues along the meridian path is very active, it can be speculated that the production of reactive oxygen species is also increased in these tissues. Guo et al. [25, 26] found that the concentration of free radicals is high in the meridian of the abdominal wall of rats. They suggested that the antioxidant reduction reaction was active in the tissues along the meridians to maintain the steady state of the local internal environment. In this study, we observed that ATP5E expression was upregulated in the ABT along the CV compared with nonmeridian tissues. It can be inferred that the production of H_2_O_2_ and peroxides (R-O-OH) is also increased and a large amount of ATP synthesis occurs thereafter. Therefore, a large number of antioxidant enzymes are needed to efficiently remove free radicals. Gpx-3 expression was upregulated in the ABT along the CV, suggesting that the catalytic reduction reaction process may be more active in tissues along the meridian path. Gpx-3 can effectively eliminate the reactive oxygen radicals produced by the high energy metabolism of meridians.

Ca^2+^ is an important second messenger in cells. The biological activity of Ca^2+^ in acupoints and meridians is closely related to the activities of meridians and the acupuncture effects [27, 28]. The results showed that the concentration of Ca^2+^ at acupoints was higher than that at non-acupoints, and this trend was more significant after acupuncture [29]. After the Ca^2+^ along meridians was complexed to reduce the concentration of Ca^2+^, the recruitment and degranulation of mast cell was inhibited, and then the acupuncture effect on visceral regulation disappeared. Blocking the voltage-gated Ca^2+^ channel on the cell membrane of acupoint area or blocking the biological activity of calmodulin in the acupoint area can affect acupuncture effects [30]. Many studies have proven that a high content of Ca^2+^ is present in the tissues along meridians and this feature is the key to meridian phenomena and acupuncture effects. The biological function of Ca^2+^ is produced by a series of cascade reactions caused by intracellular Ca^2+^ transmembrane transport [31, 32]. The functional proteins related to Ca^2+^ transmembrane transport were differentially expressed between the ABT in CV tissues and nonmeridian tissues in rats. The change in Ca^2+^ concentration inside and outside the cell membrane is the key factor in Ca^2+^ transmembrane transport, and the important factor affecting the change in Ca^2+^ concentration is the biological activity of calcium channel proteins and calmodulin on the cell membrane [33]. As the expression of proteins related to Ca^2+^ transmembrane transport, such as CACNA2D1, was upregulated in the ABT along the CV, it is suggested that the biological process of Ca^2+^ transmembrane transport was more active in the tissues of ABT than in adjacent nonmeridian tissues. As Ca^2+^ in the tissue along meridians is relatively high, when acupuncture, massage, moxibustion, or other physical stimulations are exerted on the acupoints along meridians, nerve endings and MCs are activated to cause a local short reflection and induce the changes of mechanical pressure and voltage of local tissue which may activate the opening of voltage-gated Ca^2+^ channel proteins and increase Ca^2+^ influx. Additionally, it can cause a potential change in local tissue and excite adjacent cells, further activating the upregulation of CACNA2D1 expression in the tissues along the meridians to cause Ca^2+^ influx, triggering a series of cascade reactions. Then, the metabolism of the tissue along meridians would be increased accompanied by the release and transmission of information substances like neurotransmitters, ions, and cytokines. In the tissues along meridians, materials, information, and energy are transmitted in a chemotactic way which can produce meridian phenomena and acupuncture effects. Of course, this conjecture needs to be further verified by follow-up studies.

In the subcutaneous connective tissue of the ABT including the LHRCs along the CV, the functional proteins involved in ATP metabolism, redox reactions, and Ca^2+^ transmembrane transport were upregulated. The biological functions of these proteins may have a strong correlation with meridian phenomena and acupuncture effects. This study provided new evidence to verify and explain the theory of LHRCM at the protein level.

The research of meridian is in a low tide period at present and the study on the essence of meridians has almost been stagnated in the past ten years. Research on the expression of characteristic proteins in meridian tissues is so limited that useful information is scarce. This is the first time to study the material basis of LHRCM using differential proteomics technology. In our study, a very large differential proteomics dataset was obtained. As the information available about the material basis of meridians was limited at present, there were some limitations in the selection of characteristic proteins in the tissues of ABT along meridians. Future exploration of these differential proteomics data is necessary by paying attention to the new progress in meridian essence research and life sciences.

## 5. Conclusion

In summary, the present study demonstrates that the ATP5E, CACNA2D1, GAPDH, and Gpx-3 which were upregulated expression in ABT along meridians were closely related to the meridian phenomena and acupuncture effects. Those proteins could be regarded as the potential characteristic proteins of LHRC along the CV in rats.

## Figures and Tables

**Table 1:** Gradient elution table.

Time (min)	Phase A (%)	Phase B (%)
**0**	89	11
**25**	87.7	12.3
**56**	87.3	12.7
**83**	86	14
**101**	85	15
**141.5**	77	23
**151**	73	27
**156**	57.3	42.7
**174**	20	80
**178**	20	80
**179**	89	11
**185**	89	11

## Data Availability

The data used to support the findings of this study are included within the supplemental files.

## Abbreviations

AB: Alcian blue
ABT: Alcian blue track
ATP: Adenosine-triphosphate
ATP5E: ATP synthase subunit epsilon
CV: Conception vessel
CACNA2D1: Voltage-dependent calcium channel subunit alpha-2/delta-1
CT: Control tissue
DDA: Data-dependent acquisition
GAPDH: Glyceraldehyde-3-phosphate dehydrogenase
GO: Gene Ontology
Gpx-3: Glutathione peroxidase 3
KEGG: Kyoto Encyclopedia of Genes and Genomes
LHRC: Low hydraulic resistance channel
LHRCM: LHRC meridian
SCT: Subcutaneous connective tissues.

## Supplementary Materials

Supplementary Materials 1. The data of qualitative identification of protein. 2. The false discovery rate (FDR) of SWATH data. 3. The data of biological process (Gene Ontology (GO) analysis of differential proteins). 4. The data of cell component (GO analysis of differential proteins). 5. The data of molecular function (GO analysis of differential proteins). 6. The data of KEGG analysis. 7. The data of biological progress and KEEG link. 8. The data of protein-protein interaction (PPI). 9. The information analysis note. 10. The data of western blot analysis: the upregulated differential proteins involved in ATP metabolism (ATP5E, GAPDH), redox reactions (Gpx-3), and Ca^2+^ transmembrane transport (CACNA2D1).
