# Supplementary material for: Differential Proteomics Analysis of the Subcutaneous Connective Tissues in Alcian Blue Tracks along Conception Vessel and Adjacent Nonmeridian in Rats
Source: Evid Based Complement Alternat Med. 2021 May 4;2021:5550694. doi: 10.1155/2021/5550694 (PMC8116161; doi:10.1155/2021/5550694)
Supplement: Supplementary Materials — 1. The data of qualitative identification of protein. 2. The false discovery rate (FDR) of SWATH data. 3. The data of biological process (Gene Ontology (GO) analysis of differential proteins). 4. The data of cell component (GO analysis of differential proteins). 5. The data of molecular function (GO analysis of differential proteins). 6. The data of KEGG analysis. 7. The data of biological progress and KEEG link. 8. The data of protein-protein interaction (PPI). 9. The information analysis note. 10. The data of western blot analysis: the upregulated differential proteins involved in ATP metabolism (ATP5E, GAPDH), redox reactions (Gpx-3), and Ca2+ transmembrane transport (CACNA2D1). [file 5550694.f1.zip › 5550694.f1/10-WB original data.docx]

| RM |  |  |  |  |  |  |  |
| --- | --- | --- | --- | --- | --- | --- | --- |
| ATP | CACNA2D1 | β-actin | ATP | CACNA2D1 |  |  |  |
| 194777 | 234188 | 292224 | 0.666533 | 0.801399 |  |  |  |
| 105736 | 331775 | 345727 | 0.305837 | 0.959644 |  |  |  |
| 268347 | 382419 | 311953 | 0.860216 | 1.225887 |  |  |  |
| 131869 | 258482 | 296731 | 0.444406 | 0.871099 |  |  |  |
| 133027 | 295452 | 373812 | 0.355866 | 0.790376 |  |  |  |
| 236444 | 492081 | 325686 | 0.725988 | 1.510906 |  |  |  |
| 188365 | 405659 | 347068 | 0.542732 | 1.168817 |  |  |  |
| 123517 | 56770 | 284384 | 0.434332 | 0.199624 |  |  |  |
|  |  |  | 0.541989 | 0.940969 |  |  |  |
| CM |  |  |  |  |  |  |  |
| ATP | CACNA2D1 | β-actin | ATP | CACNA2D1 |  |  |  |
| 16684 | 52560 | 325513 | 0.051254 | 0.161468 |  |  |  |
| 111662 | 118875 | 399603 | 0.279432 | 0.297483 |  |  |  |
| 60442 | 46230 | 354012 | 0.170734 | 0.130589 |  |  |  |
| 20213 | 176364 | 315864 | 0.063993 | 0.558354 |  |  |  |
| 77569 | 38525 | 302806 | 0.256167 | 0.127227 |  |  |  |
| 81969 | 158476 | 303550 | 0.270035 | 0.522075 |  |  |  |
| 84825 | 35637 | 251707 | 0.336999 | 0.141581 |  |  |  |
| 119633 | 171455 | 317740 | 0.376512 | 0.539608 |  |  |  |
|  |  |  | 0.225641 | 0.309798 |  |  |  |
|  |  |  |  |  |  |  |  |
|  |  |  |  |  |  |  |  |
| CM |  |  |  | RM |  |  |  |
| GAPDH | β-actin | GAPDH/β-actin | | Gpx-3 | β-actin | GAPDH/β-actin | |
| 32363 | 296825 | 0.109031 |  | 219471 | 296825 | 0.739395 |  |
| 31570 | 378711 | 0.083362 |  | 328281 | 378711 | 0.866838 |  |
| 24495 | 322521 | 0.075949 |  | 295175 | 322521 | 0.915212 |  |
| 180326 | 361501 | 0.498826 |  | 181217 | 361501 | 0.50129 |  |
| 22519 | 329147 | 0.068416 |  | 179946 | 329147 | 0.546704 |  |
| 81651 | 321106 | 0.254281 |  | 280889 | 321106 | 0.874755 |  |
| 187425 | 299969 | 0.624815 |  | 444838 | 299969 | 1.482947 |  |
| 116215 | 346665 | 0.335237 |  | 338442 | 346665 | 0.97628 |  |
|  |  | 0.256239 |  |  |  | 0.862928 |  |
| RM |  |  |  | CM |  |  |  |
| GAPDH | β-actin | GAPDH/β-actin | | Gpx-3 | β-actin | GAPDH/β-actin | |
| 240544 | 409253 | 0.587764 |  | 291372 | 409253 | 0.711961 |  |
| 196666 | 383571 | 0.512724 |  | 307843 | 383571 | 0.802571 |  |
| 286592 | 362714 | 0.790132 |  | 127325 | 362714 | 0.351034 |  |
| 253187 | 333360 | 0.7595 |  | 56989 | 333360 | 0.170953 |  |
| 183894 | 384608 | 0.478134 |  | 158985 | 384608 | 0.413369 |  |
| 286450 | 290015 | 0.987708 |  | 143284 | 290015 | 0.494057 |  |
| 222673 | 336505 | 0.661723 |  | 406569 | 336505 | 1.208211 |  |
| 24346 | 364164 | 0.066854 |  | 157393 | 364164 | 0.432204 |  |
|  |  | 0.605567 |  |  |  | 0.573045 |  |
|  |  |  |  |  |  |  |  |
