# Supplementary material for: Differential Proteomics Analysis of the Subcutaneous Connective Tissues in Alcian Blue Tracks along Conception Vessel and Adjacent Nonmeridian in Rats
Source: Evid Based Complement Alternat Med. 2021 May 4;2021:5550694. doi: 10.1155/2021/5550694 (PMC8116161; doi:10.1155/2021/5550694)
Supplement: Supplementary Materials — 1. The data of qualitative identification of protein. 2. The false discovery rate (FDR) of SWATH data. 3. The data of biological process (Gene Ontology (GO) analysis of differential proteins). 4. The data of cell component (GO analysis of differential proteins). 5. The data of molecular function (GO analysis of differential proteins). 6. The data of KEGG analysis. 7. The data of biological progress and KEEG link. 8. The data of protein-protein interaction (PPI). 9. The information analysis note. 10. The data of western blot analysis: the upregulated differential proteins involved in ATP metabolism (ATP5E, GAPDH), redox reactions (Gpx-3), and Ca2+ transmembrane transport (CACNA2D1). [file 5550694.f1.zip › 5550694.f1/8-PPI.docx]

| **node1** | **node1_gene** | **node1_string** | **node2** | **node2_gene** | **node2_string** | **score** |
| --- | --- | --- | --- | --- | --- | --- |
| Q29RW1 | Myh4 | 10116.ENSRNOP00000046362 | P10759 | Ampd1 | 10116.ENSRNOP00000025248 | 500 |
| Q29RW1 | Myh4 | 10116.ENSRNOP00000046362 | P49134 | Itgb1 | 10116.ENSRNOP00000014785 | 539 |
| Q29RW1 | Myh4 | 10116.ENSRNOP00000046362 | P19633 | Casq1 | 10116.ENSRNOP00000009344 | 517 |
| Q29RW1 | Myh4 | 10116.ENSRNOP00000046362 | Q64119 | Myl6 | 10116.ENSRNOP00000067282 | 680 |
| Q29RW1 | Myh4 | 10116.ENSRNOP00000046362 | P18588 | Mx1 | 10116.ENSRNOP00000043001 | 402 |
| Q29RW1 | Myh4 | 10116.ENSRNOP00000046362 | P60711 | Actb | 10116.ENSRNOP00000044296 | 925 |
| Q29RW1 | Myh4 | 10116.ENSRNOP00000046362 | P63259 | Actg1 | 10116.ENSRNOP00000044296 | 925 |
| Q29RW1 | Myh4 | 10116.ENSRNOP00000046362 | P04466 | Mylpf | 10116.ENSRNOP00000023944 | 880 |
| Q29RW1 | Myh4 | 10116.ENSRNOP00000046362 | P17209 | Myl4 | 10116.ENSRNOP00000064224 | 742 |
| Q29RW1 | Myh4 | 10116.ENSRNOP00000046362 | Q8CFN2 | Cdc42 | 10116.ENSRNOP00000030928 | 583 |
| Q29RW1 | Myh4 | 10116.ENSRNOP00000046362 | P02625 | Pvalb | 10116.ENSRNOP00000058825 | 687 |
| Q29RW1 | Myh4 | 10116.ENSRNOP00000046362 | P13832 | Rlc-a | 10116.ENSRNOP00000021048 | 779 |
| Q29RW1 | Myh4 | 10116.ENSRNOP00000046362 | F1LMY4 | Ryr1 | 10116.ENSRNOP00000027893 | 424 |
| Q29RW1 | Myh4 | 10116.ENSRNOP00000046362 | Q66HD0 | Hsp90b1 | 10116.ENSRNOP00000034846 | 426 |
| P12785 | Fasn | 10116.ENSRNOP00000064445 | P13697 | Me1 | 10116.ENSRNOP00000013244 | 489 |
| P12785 | Fasn | 10116.ENSRNOP00000064445 | P55053 | Fabp5 | 10116.ENSRNOP00000064809 | 424 |
| P12785 | Fasn | 10116.ENSRNOP00000064445 | P11497 | Acaca | 10116.ENSRNOP00000049438 | 998 |
| P12785 | Fasn | 10116.ENSRNOP00000064445 | Q499N5 | Acsf2 | 10116.ENSRNOP00000004673 | 611 |
| P12785 | Fasn | 10116.ENSRNOP00000064445 | P41350 | Cav1 | 10116.ENSRNOP00000009253 | 723 |
| P12785 | Fasn | 10116.ENSRNOP00000064445 | O70351 | Hsd17b10 | 10116.ENSRNOP00000043608 | 426 |
| P12785 | Fasn | 10116.ENSRNOP00000064445 | Q64591 | Decr1 | 10116.ENSRNOP00000011330 | 606 |
| P12785 | Fasn | 10116.ENSRNOP00000064445 | P16638 | Acly | 10116.ENSRNOP00000023447 | 992 |
| P12785 | Fasn | 10116.ENSRNOP00000064445 | P52873 | Pc | 10116.ENSRNOP00000026316 | 462 |
| P12785 | Fasn | 10116.ENSRNOP00000064445 | P04143 | Thrsp | 10116.ENSRNOP00000016540 | 522 |
| Q63041 | A1m | 10116.ENSRNOP00000009467 | Q64240 | Ambp | 10116.ENSRNOP00000009248 | 441 |
| Q63041 | A1m | 10116.ENSRNOP00000009467 | Q63514 | C4bpa | 10116.ENSRNOP00000005461 | 639 |
| P31000 | Vim | 10116.ENSRNOP00000024430 | Q6IFV3 | Krt15 | 10116.ENSRNOP00000019037 | 900 |
| P31000 | Vim | 10116.ENSRNOP00000024430 | P08699 | Lgals3 | 10116.ENSRNOP00000014216 | 636 |
| P31000 | Vim | 10116.ENSRNOP00000024430 | Q6P6Q2 | Krt5 | 10116.ENSRNOP00000011644 | 901 |
| P31000 | Vim | 10116.ENSRNOP00000024430 | Q07936 | Anxa2 | 10116.ENSRNOP00000038428 | 498 |
| P31000 | Vim | 10116.ENSRNOP00000024430 | P15684 | Anpep | 10116.ENSRNOP00000020002 | 560 |
| P31000 | Vim | 10116.ENSRNOP00000024430 | P60711 | Actb | 10116.ENSRNOP00000044296 | 553 |
| P31000 | Vim | 10116.ENSRNOP00000024430 | P63259 | Actg1 | 10116.ENSRNOP00000044296 | 553 |
| P31000 | Vim | 10116.ENSRNOP00000024430 | P04797 | Gapdh | 10116.ENSRNOP00000040878 | 412 |
| P31000 | Vim | 10116.ENSRNOP00000024430 | P11762 | Lgals1 | 10116.ENSRNOP00000013538 | 504 |
| P31000 | Vim | 10116.ENSRNOP00000024430 | Q6IFU8 | Krt17 | 10116.ENSRNOP00000005382 | 903 |
| P02091 | Hbb | 10116.ENSRNOP00000048250 | Q5XIC0 | Eci2 | 10116.ENSRNOP00000022022 | 449 |
| P02091 | Hbb | 10116.ENSRNOP00000048250 | P23965 | Eci1 | 10116.ENSRNOP00000011784 | 428 |
| P02091 | Hbb | 10116.ENSRNOP00000048250 | Q6AYG5 | Echdc1 | 10116.ENSRNOP00000015440 | 422 |
| P02091 | Hbb | 10116.ENSRNOP00000048250 | P06866 | Hp | 10116.ENSRNOP00000049003 | 498 |
| P02091 | Hbb | 10116.ENSRNOP00000048250 | P30839 | Aldh3a2 | 10116.ENSRNOP00000061762 | 405 |
| P02091 | Hbb | 10116.ENSRNOP00000048250 | P04797 | Gapdh | 10116.ENSRNOP00000040878 | 529 |
| P18163 | Acsl1 | 10116.ENSRNOP00000014235 | P07483 | Fabp3 | 10116.ENSRNOP00000017325 | 709 |
| P18163 | Acsl1 | 10116.ENSRNOP00000014235 | P10960 | Psap | 10116.ENSRNOP00000000696 | 471 |
| P18163 | Acsl1 | 10116.ENSRNOP00000014235 | P00507 | Got2 | 10116.ENSRNOP00000015956 | 517 |
| P18163 | Acsl1 | 10116.ENSRNOP00000014235 | P11497 | Acaca | 10116.ENSRNOP00000049438 | 725 |
| P18163 | Acsl1 | 10116.ENSRNOP00000014235 | P08503 | Acadm | 10116.ENSRNOP00000013238 | 882 |
| P18163 | Acsl1 | 10116.ENSRNOP00000014235 | Q8K4D8 | Aldh1a3 | 10116.ENSRNOP00000045261 | 450 |
| P18163 | Acsl1 | 10116.ENSRNOP00000014235 | Q04462 | Vars | 10116.ENSRNOP00000001160 | 471 |
| P18163 | Acsl1 | 10116.ENSRNOP00000014235 | P28037 | Aldh1l1 | 10116.ENSRNOP00000065882 | 437 |
| P18163 | Acsl1 | 10116.ENSRNOP00000014235 | O55171 | Acot2 | 10116.ENSRNOP00000013515 | 551 |
| P18163 | Acsl1 | 10116.ENSRNOP00000014235 | P97852 | Hsd17b4 | 10116.ENSRNOP00000021646 | 488 |
| P18163 | Acsl1 | 10116.ENSRNOP00000014235 | P30839 | Aldh3a2 | 10116.ENSRNOP00000061762 | 489 |
| P18163 | Acsl1 | 10116.ENSRNOP00000014235 | P23965 | Eci1 | 10116.ENSRNOP00000011784 | 587 |
| P18163 | Acsl1 | 10116.ENSRNOP00000014235 | Q9JLJ3 | Aldh9a1 | 10116.ENSRNOP00000005611 | 455 |
| P18163 | Acsl1 | 10116.ENSRNOP00000014235 | P45953 | Acadvl | 10116.ENSRNOP00000024973 | 801 |
| P18163 | Acsl1 | 10116.ENSRNOP00000014235 | Q6AYG5 | Echdc1 | 10116.ENSRNOP00000015440 | 456 |
| P18163 | Acsl1 | 10116.ENSRNOP00000014235 | Q62969 | Ptgis | 10116.ENSRNOP00000010891 | 435 |
| P18163 | Acsl1 | 10116.ENSRNOP00000014235 | Q5XIC0 | Eci2 | 10116.ENSRNOP00000022022 | 660 |
| P18163 | Acsl1 | 10116.ENSRNOP00000014235 | P16638 | Acly | 10116.ENSRNOP00000023447 | 507 |
| P46462 | Vcp | 10116.ENSRNOP00000040121 | Q5I0H9 | Pdia5 | 10116.ENSRNOP00000059945 | 440 |
| P46462 | Vcp | 10116.ENSRNOP00000040121 | Q63081 | Pdia6 | 10116.ENSRNOP00000064632 | 541 |
| P46462 | Vcp | 10116.ENSRNOP00000040121 | P41350 | Cav1 | 10116.ENSRNOP00000009253 | 453 |
| P46462 | Vcp | 10116.ENSRNOP00000040121 | Q4KMA2 | Rad23b | 10116.ENSRNOP00000021629 | 875 |
| P46462 | Vcp | 10116.ENSRNOP00000040121 | P62193 | Psmc1 | 10116.ENSRNOP00000005329 | 710 |
| P46462 | Vcp | 10116.ENSRNOP00000040121 | Q66HD0 | Hsp90b1 | 10116.ENSRNOP00000034846 | 486 |
| P46462 | Vcp | 10116.ENSRNOP00000040121 | Q9Z269 | Vapb | 10116.ENSRNOP00000007554 | 726 |
| P46462 | Vcp | 10116.ENSRNOP00000040121 | P17220 | Psma2 | 10116.ENSRNOP00000066950 | 589 |
| Q07936 | Anxa2 | 10116.ENSRNOP00000038428 | P41350 | Cav1 | 10116.ENSRNOP00000009253 | 727 |
| Q07936 | Anxa2 | 10116.ENSRNOP00000038428 | Q6B345 | S100a11 | 10116.ENSRNOP00000013393 | 533 |
| Q07936 | Anxa2 | 10116.ENSRNOP00000038428 | P08699 | Lgals3 | 10116.ENSRNOP00000014216 | 625 |
| Q07936 | Anxa2 | 10116.ENSRNOP00000038428 | P14668 | Anxa5 | 10116.ENSRNOP00000019552 | 448 |
| Q07936 | Anxa2 | 10116.ENSRNOP00000038428 | Q8CFN2 | Cdc42 | 10116.ENSRNOP00000030928 | 785 |
| Q07936 | Anxa2 | 10116.ENSRNOP00000038428 | Q01177 | Plg | 10116.ENSRNOP00000023370 | 796 |
| Q07936 | Anxa2 | 10116.ENSRNOP00000038428 | Q66HD0 | Hsp90b1 | 10116.ENSRNOP00000034846 | 518 |
| Q07936 | Anxa2 | 10116.ENSRNOP00000038428 | P11762 | Lgals1 | 10116.ENSRNOP00000013538 | 424 |
| P04466 | Mylpf | 10116.ENSRNOP00000023944 | Q64119 | Myl6 | 10116.ENSRNOP00000067282 | 817 |
| P04466 | Mylpf | 10116.ENSRNOP00000023944 | Q8CFN2 | Cdc42 | 10116.ENSRNOP00000030928 | 519 |
| P04466 | Mylpf | 10116.ENSRNOP00000023944 | P60711 | Actb | 10116.ENSRNOP00000044296 | 869 |
| P04466 | Mylpf | 10116.ENSRNOP00000023944 | P63259 | Actg1 | 10116.ENSRNOP00000044296 | 869 |
| P04466 | Mylpf | 10116.ENSRNOP00000023944 | P17209 | Myl4 | 10116.ENSRNOP00000064224 | 795 |
| P04466 | Mylpf | 10116.ENSRNOP00000023944 | P10759 | Ampd1 | 10116.ENSRNOP00000025248 | 443 |
| P04466 | Mylpf | 10116.ENSRNOP00000023944 | P19633 | Casq1 | 10116.ENSRNOP00000009344 | 650 |
| P04466 | Mylpf | 10116.ENSRNOP00000023944 | P15429 | Eno3 | 10116.ENSRNOP00000005612 | 463 |
| Q01177 | Plg | 10116.ENSRNOP00000023370 | P36953 | Afm | 10116.ENSRNOP00000057275 | 496 |
| Q01177 | Plg | 10116.ENSRNOP00000023370 | Q63514 | C4bpa | 10116.ENSRNOP00000005461 | 788 |
| Q01177 | Plg | 10116.ENSRNOP00000023370 | P02680 | Fgg | 10116.ENSRNOP00000032735 | 860 |
| Q01177 | Plg | 10116.ENSRNOP00000023370 | P04797 | Gapdh | 10116.ENSRNOP00000040878 | 661 |
| Q01177 | Plg | 10116.ENSRNOP00000023370 | Q64240 | Ambp | 10116.ENSRNOP00000009248 | 807 |
| Q01177 | Plg | 10116.ENSRNOP00000023370 | P04636 | Mdh2 | 10116.ENSRNOP00000001958 | 618 |
| Q01177 | Plg | 10116.ENSRNOP00000023370 | P15429 | Eno3 | 10116.ENSRNOP00000005612 | 573 |
| Q01177 | Plg | 10116.ENSRNOP00000023370 | Q6IG05 | Krt75 | 10116.ENSRNOP00000012640 | 417 |
| Q01177 | Plg | 10116.ENSRNOP00000023370 | Q8CFN2 | Cdc42 | 10116.ENSRNOP00000030928 | 479 |
| P52873 | Pc | 10116.ENSRNOP00000026316 | Q497B0 | Nit2 | 10116.ENSRNOP00000034144 | 837 |
| P52873 | Pc | 10116.ENSRNOP00000026316 | P16638 | Acly | 10116.ENSRNOP00000023447 | 966 |
| P52873 | Pc | 10116.ENSRNOP00000026316 | P15337 | Creb1 | 10116.ENSRNOP00000018326 | 823 |
| P52873 | Pc | 10116.ENSRNOP00000026316 | P49432 | Pdhb | 10116.ENSRNOP00000010545 | 921 |
| P52873 | Pc | 10116.ENSRNOP00000026316 | P00507 | Got2 | 10116.ENSRNOP00000015956 | 877 |
| P52873 | Pc | 10116.ENSRNOP00000026316 | Q66HG6 | Ca5b | 10116.ENSRNOP00000043235 | 453 |
| P52873 | Pc | 10116.ENSRNOP00000026316 | P48500 | Tpi1 | 10116.ENSRNOP00000067409 | 462 |
| P52873 | Pc | 10116.ENSRNOP00000026316 | P13697 | Me1 | 10116.ENSRNOP00000013244 | 953 |
| P52873 | Pc | 10116.ENSRNOP00000026316 | P14141 | Ca3 | 10116.ENSRNOP00000014177 | 453 |
| P52873 | Pc | 10116.ENSRNOP00000026316 | O88989 | Mdh1 | 10116.ENSRNOP00000011429 | 969 |
| P52873 | Pc | 10116.ENSRNOP00000026316 | P11497 | Acaca | 10116.ENSRNOP00000049438 | 662 |
| P52873 | Pc | 10116.ENSRNOP00000026316 | P04797 | Gapdh | 10116.ENSRNOP00000040878 | 443 |
| P52873 | Pc | 10116.ENSRNOP00000026316 | B0BNN3 | Ca1 | 10116.ENSRNOP00000014267 | 495 |
| P52873 | Pc | 10116.ENSRNOP00000026316 | P45953 | Acadvl | 10116.ENSRNOP00000024973 | 418 |
| P52873 | Pc | 10116.ENSRNOP00000026316 | P04636 | Mdh2 | 10116.ENSRNOP00000001958 | 978 |
| P52873 | Pc | 10116.ENSRNOP00000026316 | P27139 | Ca2 | 10116.ENSRNOP00000013354 | 468 |
| P16638 | Acly | 10116.ENSRNOP00000023447 | Q9EQS0 | Taldo1 | 10116.ENSRNOP00000024863 | 463 |
| P16638 | Acly | 10116.ENSRNOP00000023447 | P13697 | Me1 | 10116.ENSRNOP00000013244 | 889 |
| P16638 | Acly | 10116.ENSRNOP00000023447 | P41562 | Idh1 | 10116.ENSRNOP00000020322 | 952 |
| P16638 | Acly | 10116.ENSRNOP00000023447 | Q7TPB1 | Cct4 | 10116.ENSRNOP00000012847 | 589 |
| P16638 | Acly | 10116.ENSRNOP00000023447 | G3V9R8 | Hnrnpc | 10116.ENSRNOP00000057257 | 523 |
| P16638 | Acly | 10116.ENSRNOP00000023447 | P04797 | Gapdh | 10116.ENSRNOP00000040878 | 592 |
| P16638 | Acly | 10116.ENSRNOP00000023447 | Q7TQ94 | Nit1 | 10116.ENSRNOP00000005194 | 466 |
| P16638 | Acly | 10116.ENSRNOP00000023447 | P23965 | Eci1 | 10116.ENSRNOP00000011784 | 503 |
| P16638 | Acly | 10116.ENSRNOP00000023447 | P13803 | Etfa | 10116.ENSRNOP00000020544 | 604 |
| P16638 | Acly | 10116.ENSRNOP00000023447 | Q497B0 | Nit2 | 10116.ENSRNOP00000034144 | 888 |
| P16638 | Acly | 10116.ENSRNOP00000023447 | O35244 | Prdx6 | 10116.ENSRNOP00000030323 | 575 |
| P16638 | Acly | 10116.ENSRNOP00000023447 | P11497 | Acaca | 10116.ENSRNOP00000049438 | 994 |
| P16638 | Acly | 10116.ENSRNOP00000023447 | P15429 | Eno3 | 10116.ENSRNOP00000005612 | 838 |
| P16638 | Acly | 10116.ENSRNOP00000023447 | P85834 | Tufm | 10116.ENSRNOP00000025203 | 579 |
| P16638 | Acly | 10116.ENSRNOP00000023447 | P04636 | Mdh2 | 10116.ENSRNOP00000001958 | 999 |
| P16638 | Acly | 10116.ENSRNOP00000023447 | Q6P799 | Sars | 10116.ENSRNOP00000038448 | 516 |
| P16638 | Acly | 10116.ENSRNOP00000023447 | Q9ER34 | Aco2 | 10116.ENSRNOP00000029144 | 998 |
| P16638 | Acly | 10116.ENSRNOP00000023447 | Q6P6V0 | Gpi | 10116.ENSRNOP00000029515 | 528 |
| P16638 | Acly | 10116.ENSRNOP00000023447 | P35738 | Bckdhb | 10116.ENSRNOP00000013249 | 803 |
| P16638 | Acly | 10116.ENSRNOP00000023447 | Q68FU3 | Etfb | 10116.ENSRNOP00000024083 | 607 |
| P16638 | Acly | 10116.ENSRNOP00000023447 | P62832 | Rpl23 | 10116.ENSRNOP00000005471 | 525 |
| P16638 | Acly | 10116.ENSRNOP00000023447 | P49432 | Pdhb | 10116.ENSRNOP00000010545 | 834 |
| P16638 | Acly | 10116.ENSRNOP00000023447 | P00507 | Got2 | 10116.ENSRNOP00000015956 | 904 |
| P16638 | Acly | 10116.ENSRNOP00000023447 | P97852 | Hsd17b4 | 10116.ENSRNOP00000021646 | 553 |
| P16638 | Acly | 10116.ENSRNOP00000023447 | P31399 | Atp5h | 10116.ENSRNOP00000004836 | 400 |
| P16638 | Acly | 10116.ENSRNOP00000023447 | P35571 | Gpd2 | 10116.ENSRNOP00000043749 | 883 |
| P16638 | Acly | 10116.ENSRNOP00000023447 | Q5XIC0 | Eci2 | 10116.ENSRNOP00000022022 | 536 |
| P16638 | Acly | 10116.ENSRNOP00000023447 | Q8K4D8 | Aldh1a3 | 10116.ENSRNOP00000045261 | 635 |
| P16638 | Acly | 10116.ENSRNOP00000023447 | Q66HD0 | Hsp90b1 | 10116.ENSRNOP00000034846 | 513 |
| P16638 | Acly | 10116.ENSRNOP00000023447 | P32089 | Slc25a1 | 10116.ENSRNOP00000000306 | 963 |
| P16638 | Acly | 10116.ENSRNOP00000023447 | Q6P6R2 | Dld | 10116.ENSRNOP00000008980 | 910 |
| P16638 | Acly | 10116.ENSRNOP00000023447 | Q68FR6 | Eef1g | 10116.ENSRNOP00000027305 | 554 |
| P16638 | Acly | 10116.ENSRNOP00000023447 | Q68FQ0 | Cct5 | 10116.ENSRNOP00000015886 | 589 |
| P16638 | Acly | 10116.ENSRNOP00000023447 | P48500 | Tpi1 | 10116.ENSRNOP00000067409 | 676 |
| P16638 | Acly | 10116.ENSRNOP00000023447 | P18445 | Rpl27a | 10116.ENSRNOP00000019247 | 506 |
| P16638 | Acly | 10116.ENSRNOP00000023447 | P08503 | Acadm | 10116.ENSRNOP00000013238 | 684 |
| P16638 | Acly | 10116.ENSRNOP00000023447 | P25113 | Pgam1 | 10116.ENSRNOP00000065690 | 528 |
| P16638 | Acly | 10116.ENSRNOP00000023447 | Q4KMA2 | Rad23b | 10116.ENSRNOP00000021629 | 727 |
| P16638 | Acly | 10116.ENSRNOP00000023447 | Q6AYG5 | Echdc1 | 10116.ENSRNOP00000015440 | 570 |
| P16638 | Acly | 10116.ENSRNOP00000023447 | Q8CFN2 | Cdc42 | 10116.ENSRNOP00000030928 | 544 |
| P16638 | Acly | 10116.ENSRNOP00000023447 | Q499N5 | Acsf2 | 10116.ENSRNOP00000004673 | 600 |
| P16638 | Acly | 10116.ENSRNOP00000023447 | P30839 | Aldh3a2 | 10116.ENSRNOP00000061762 | 635 |
| P16638 | Acly | 10116.ENSRNOP00000023447 | P28037 | Aldh1l1 | 10116.ENSRNOP00000065882 | 635 |
| P16638 | Acly | 10116.ENSRNOP00000023447 | P50137 | Tkt | 10116.ENSRNOP00000021862 | 558 |
| P16638 | Acly | 10116.ENSRNOP00000023447 | Q63081 | Pdia6 | 10116.ENSRNOP00000064632 | 672 |
| P16638 | Acly | 10116.ENSRNOP00000023447 | P41565 | Idh3g | 10116.ENSRNOP00000053220 | 967 |
| P16638 | Acly | 10116.ENSRNOP00000023447 | P08461 | Dlat | 10116.ENSRNOP00000032890 | 987 |
| P16638 | Acly | 10116.ENSRNOP00000023447 | P26772 | Hspe1 | 10116.ENSRNOP00000020066 | 620 |
| P16638 | Acly | 10116.ENSRNOP00000023447 | P05708 | Hk1 | 10116.ENSRNOP00000066611 | 420 |
| P16638 | Acly | 10116.ENSRNOP00000023447 | P60892 | Prps1 | 10116.ENSRNOP00000029405 | 573 |
| P16638 | Acly | 10116.ENSRNOP00000023447 | Q9JLJ3 | Aldh9a1 | 10116.ENSRNOP00000005611 | 656 |
| P16638 | Acly | 10116.ENSRNOP00000023447 | P45953 | Acadvl | 10116.ENSRNOP00000024973 | 638 |
| P16638 | Acly | 10116.ENSRNOP00000023447 | O88989 | Mdh1 | 10116.ENSRNOP00000011429 | 999 |
| P16638 | Acly | 10116.ENSRNOP00000023447 | P25409 | Gpt | 10116.ENSRNOP00000044411 | 484 |
| P16638 | Acly | 10116.ENSRNOP00000023447 | Q5I0H9 | Pdia5 | 10116.ENSRNOP00000059945 | 640 |
| P11497 | Acaca | 10116.ENSRNOP00000049438 | Q7TQ94 | Nit1 | 10116.ENSRNOP00000005194 | 447 |
| P11497 | Acaca | 10116.ENSRNOP00000049438 | Q5XIC0 | Eci2 | 10116.ENSRNOP00000022022 | 890 |
| P11497 | Acaca | 10116.ENSRNOP00000049438 | Q497B0 | Nit2 | 10116.ENSRNOP00000034144 | 447 |
| P11497 | Acaca | 10116.ENSRNOP00000049438 | O88989 | Mdh1 | 10116.ENSRNOP00000011429 | 734 |
| P11497 | Acaca | 10116.ENSRNOP00000049438 | P08503 | Acadm | 10116.ENSRNOP00000013238 | 800 |
| P11497 | Acaca | 10116.ENSRNOP00000049438 | Q04462 | Vars | 10116.ENSRNOP00000001160 | 483 |
| P11497 | Acaca | 10116.ENSRNOP00000049438 | P13803 | Etfa | 10116.ENSRNOP00000020544 | 594 |
| P11497 | Acaca | 10116.ENSRNOP00000049438 | P15429 | Eno3 | 10116.ENSRNOP00000005612 | 547 |
| P11497 | Acaca | 10116.ENSRNOP00000049438 | P49432 | Pdhb | 10116.ENSRNOP00000010545 | 813 |
| P11497 | Acaca | 10116.ENSRNOP00000049438 | P41565 | Idh3g | 10116.ENSRNOP00000053220 | 770 |
| P11497 | Acaca | 10116.ENSRNOP00000049438 | Q62969 | Ptgis | 10116.ENSRNOP00000010891 | 422 |
| P11497 | Acaca | 10116.ENSRNOP00000049438 | P45953 | Acadvl | 10116.ENSRNOP00000024973 | 788 |
| P11497 | Acaca | 10116.ENSRNOP00000049438 | P08461 | Dlat | 10116.ENSRNOP00000032890 | 752 |
| P11497 | Acaca | 10116.ENSRNOP00000049438 | P62752 | Rpl23a | 10116.ENSRNOP00000036391 | 503 |
| P11497 | Acaca | 10116.ENSRNOP00000049438 | Q9JLJ3 | Aldh9a1 | 10116.ENSRNOP00000005611 | 635 |
| P11497 | Acaca | 10116.ENSRNOP00000049438 | Q499N5 | Acsf2 | 10116.ENSRNOP00000004673 | 691 |
| P11497 | Acaca | 10116.ENSRNOP00000049438 | Q66HG6 | Ca5b | 10116.ENSRNOP00000043235 | 505 |
| P11497 | Acaca | 10116.ENSRNOP00000049438 | P15337 | Creb1 | 10116.ENSRNOP00000018326 | 402 |
| P11497 | Acaca | 10116.ENSRNOP00000049438 | P14141 | Ca3 | 10116.ENSRNOP00000014177 | 587 |
| P11497 | Acaca | 10116.ENSRNOP00000049438 | P50878 | Rpl4 | 10116.ENSRNOP00000013462 | 508 |
| P11497 | Acaca | 10116.ENSRNOP00000049438 | B0BNN3 | Ca1 | 10116.ENSRNOP00000014267 | 511 |
| P11497 | Acaca | 10116.ENSRNOP00000049438 | P97852 | Hsd17b4 | 10116.ENSRNOP00000021646 | 930 |
| P11497 | Acaca | 10116.ENSRNOP00000049438 | Q6AYG5 | Echdc1 | 10116.ENSRNOP00000015440 | 773 |
| P11497 | Acaca | 10116.ENSRNOP00000049438 | P06686 | Atp1a2 | 10116.ENSRNOP00000054947 | 413 |
| P11497 | Acaca | 10116.ENSRNOP00000049438 | P04797 | Gapdh | 10116.ENSRNOP00000040878 | 664 |
| P11497 | Acaca | 10116.ENSRNOP00000049438 | Q8K4D8 | Aldh1a3 | 10116.ENSRNOP00000045261 | 600 |
| P11497 | Acaca | 10116.ENSRNOP00000049438 | P13697 | Me1 | 10116.ENSRNOP00000013244 | 766 |
| P11497 | Acaca | 10116.ENSRNOP00000049438 | Q7TPB1 | Cct4 | 10116.ENSRNOP00000012847 | 807 |
| P11497 | Acaca | 10116.ENSRNOP00000049438 | P23965 | Eci1 | 10116.ENSRNOP00000011784 | 783 |
| P11497 | Acaca | 10116.ENSRNOP00000049438 | P35738 | Bckdhb | 10116.ENSRNOP00000013249 | 813 |
| P11497 | Acaca | 10116.ENSRNOP00000049438 | Q64591 | Decr1 | 10116.ENSRNOP00000011330 | 550 |
| P11497 | Acaca | 10116.ENSRNOP00000049438 | P30839 | Aldh3a2 | 10116.ENSRNOP00000061762 | 600 |
| P11497 | Acaca | 10116.ENSRNOP00000049438 | Q68FQ0 | Cct5 | 10116.ENSRNOP00000015886 | 808 |
| P11497 | Acaca | 10116.ENSRNOP00000049438 | Q02253 | Aldh6a1 | 10116.ENSRNOP00000015545 | 911 |
| P11497 | Acaca | 10116.ENSRNOP00000049438 | P12007 | Ivd | 10116.ENSRNOP00000013829 | 672 |
| P11497 | Acaca | 10116.ENSRNOP00000049438 | Q6P7Q4 | Glo1 | 10116.ENSRNOP00000000650 | 475 |
| P11497 | Acaca | 10116.ENSRNOP00000049438 | P28037 | Aldh1l1 | 10116.ENSRNOP00000065882 | 635 |
| P11497 | Acaca | 10116.ENSRNOP00000049438 | Q4KMA2 | Rad23b | 10116.ENSRNOP00000021629 | 704 |
| P11497 | Acaca | 10116.ENSRNOP00000049438 | P48500 | Tpi1 | 10116.ENSRNOP00000067409 | 415 |
| P11497 | Acaca | 10116.ENSRNOP00000049438 | Q66HD0 | Hsp90b1 | 10116.ENSRNOP00000034846 | 419 |
| P11497 | Acaca | 10116.ENSRNOP00000049438 | P60868 | Rps20 | 10116.ENSRNOP00000066077 | 416 |
| P11497 | Acaca | 10116.ENSRNOP00000049438 | P26772 | Hspe1 | 10116.ENSRNOP00000020066 | 421 |
| P11497 | Acaca | 10116.ENSRNOP00000049438 | P60892 | Prps1 | 10116.ENSRNOP00000029405 | 492 |
| P11497 | Acaca | 10116.ENSRNOP00000049438 | P25409 | Gpt | 10116.ENSRNOP00000044411 | 598 |
| P11497 | Acaca | 10116.ENSRNOP00000049438 | Q9ER34 | Aco2 | 10116.ENSRNOP00000029144 | 506 |
| P11497 | Acaca | 10116.ENSRNOP00000049438 | P04636 | Mdh2 | 10116.ENSRNOP00000001958 | 734 |
| P11497 | Acaca | 10116.ENSRNOP00000049438 | P27139 | Ca2 | 10116.ENSRNOP00000013354 | 509 |
| P11497 | Acaca | 10116.ENSRNOP00000049438 | Q6P6V0 | Gpi | 10116.ENSRNOP00000029515 | 499 |
| P11497 | Acaca | 10116.ENSRNOP00000049438 | P41562 | Idh1 | 10116.ENSRNOP00000020322 | 451 |
| P15429 | Eno3 | 10116.ENSRNOP00000005612 | Q9Z2L0 | Vdac1 | 10116.ENSRNOP00000008477 | 417 |
| P15429 | Eno3 | 10116.ENSRNOP00000005612 | O88989 | Mdh1 | 10116.ENSRNOP00000011429 | 784 |
| P15429 | Eno3 | 10116.ENSRNOP00000005612 | P60868 | Rps20 | 10116.ENSRNOP00000066077 | 417 |
| P15429 | Eno3 | 10116.ENSRNOP00000005612 | P41562 | Idh1 | 10116.ENSRNOP00000020322 | 410 |
| P15429 | Eno3 | 10116.ENSRNOP00000005612 | P25113 | Pgam1 | 10116.ENSRNOP00000065690 | 985 |
| P15429 | Eno3 | 10116.ENSRNOP00000005612 | Q6P6R2 | Dld | 10116.ENSRNOP00000008980 | 708 |
| P15429 | Eno3 | 10116.ENSRNOP00000005612 | P04636 | Mdh2 | 10116.ENSRNOP00000001958 | 835 |
| P15429 | Eno3 | 10116.ENSRNOP00000005612 | P13803 | Etfa | 10116.ENSRNOP00000020544 | 573 |
| P15429 | Eno3 | 10116.ENSRNOP00000005612 | Q9ER34 | Aco2 | 10116.ENSRNOP00000029144 | 428 |
| P15429 | Eno3 | 10116.ENSRNOP00000005612 | P50137 | Tkt | 10116.ENSRNOP00000021862 | 887 |
| P15429 | Eno3 | 10116.ENSRNOP00000005612 | P05708 | Hk1 | 10116.ENSRNOP00000066611 | 480 |
| P15429 | Eno3 | 10116.ENSRNOP00000005612 | Q7M0E3 | Dstn | 10116.ENSRNOP00000007794 | 513 |
| P15429 | Eno3 | 10116.ENSRNOP00000005612 | Q68FQ0 | Cct5 | 10116.ENSRNOP00000015886 | 426 |
| P15429 | Eno3 | 10116.ENSRNOP00000005612 | P41350 | Cav1 | 10116.ENSRNOP00000009253 | 518 |
| P15429 | Eno3 | 10116.ENSRNOP00000005612 | P10111 | Ppia | 10116.ENSRNOP00000009407 | 427 |
| P15429 | Eno3 | 10116.ENSRNOP00000005612 | P62914 | Rpl11 | 10116.ENSRNOP00000037110 | 404 |
| P15429 | Eno3 | 10116.ENSRNOP00000005612 | Q6P6V0 | Gpi | 10116.ENSRNOP00000029515 | 978 |
| P15429 | Eno3 | 10116.ENSRNOP00000005612 | P08461 | Dlat | 10116.ENSRNOP00000032890 | 541 |
| P15429 | Eno3 | 10116.ENSRNOP00000005612 | P49432 | Pdhb | 10116.ENSRNOP00000010545 | 420 |
| P15429 | Eno3 | 10116.ENSRNOP00000005612 | Q68FR6 | Eef1g | 10116.ENSRNOP00000027305 | 794 |
| P15429 | Eno3 | 10116.ENSRNOP00000005612 | Q7TPB1 | Cct4 | 10116.ENSRNOP00000012847 | 436 |
| P15429 | Eno3 | 10116.ENSRNOP00000005612 | A0JPJ7 | Ola1 | 10116.ENSRNOP00000026040 | 436 |
| P15429 | Eno3 | 10116.ENSRNOP00000005612 | P48500 | Tpi1 | 10116.ENSRNOP00000067409 | 991 |
| P15429 | Eno3 | 10116.ENSRNOP00000005612 | P04797 | Gapdh | 10116.ENSRNOP00000040878 | 954 |
| P15429 | Eno3 | 10116.ENSRNOP00000005612 | P35427 | Rpl13a | 10116.ENSRNOP00000027976 | 560 |
| P15429 | Eno3 | 10116.ENSRNOP00000005612 | P00507 | Got2 | 10116.ENSRNOP00000015956 | 432 |
| P15429 | Eno3 | 10116.ENSRNOP00000005612 | Q9EQS0 | Taldo1 | 10116.ENSRNOP00000024863 | 903 |
| P15429 | Eno3 | 10116.ENSRNOP00000005612 | P50878 | Rpl4 | 10116.ENSRNOP00000013462 | 451 |
| P15429 | Eno3 | 10116.ENSRNOP00000005612 | Q04462 | Vars | 10116.ENSRNOP00000001160 | 594 |
| P15429 | Eno3 | 10116.ENSRNOP00000005612 | P10759 | Ampd1 | 10116.ENSRNOP00000025248 | 424 |
| P15429 | Eno3 | 10116.ENSRNOP00000005612 | P60711 | Actb | 10116.ENSRNOP00000044296 | 473 |
| P15429 | Eno3 | 10116.ENSRNOP00000005612 | P63259 | Actg1 | 10116.ENSRNOP00000044296 | 473 |
| Q9ER34 | Aco2 | 10116.ENSRNOP00000029144 | P48500 | Tpi1 | 10116.ENSRNOP00000067409 | 513 |
| Q9ER34 | Aco2 | 10116.ENSRNOP00000029144 | O88989 | Mdh1 | 10116.ENSRNOP00000011429 | 656 |
| Q9ER34 | Aco2 | 10116.ENSRNOP00000029144 | Q6P6R2 | Dld | 10116.ENSRNOP00000008980 | 706 |
| Q9ER34 | Aco2 | 10116.ENSRNOP00000029144 | O35077 | Gpd1 | 10116.ENSRNOP00000026200 | 462 |
| Q9ER34 | Aco2 | 10116.ENSRNOP00000029144 | P28037 | Aldh1l1 | 10116.ENSRNOP00000065882 | 471 |
| Q9ER34 | Aco2 | 10116.ENSRNOP00000029144 | P41565 | Idh3g | 10116.ENSRNOP00000053220 | 997 |
| Q9ER34 | Aco2 | 10116.ENSRNOP00000029144 | Q8K4D8 | Aldh1a3 | 10116.ENSRNOP00000045261 | 471 |
| Q9ER34 | Aco2 | 10116.ENSRNOP00000029144 | P49432 | Pdhb | 10116.ENSRNOP00000010545 | 568 |
| Q9ER34 | Aco2 | 10116.ENSRNOP00000029144 | P41562 | Idh1 | 10116.ENSRNOP00000020322 | 990 |
| Q9ER34 | Aco2 | 10116.ENSRNOP00000029144 | P35745 | Acyp2 | 10116.ENSRNOP00000061028 | 489 |
| Q9ER34 | Aco2 | 10116.ENSRNOP00000029144 | P08461 | Dlat | 10116.ENSRNOP00000032890 | 506 |
| Q9ER34 | Aco2 | 10116.ENSRNOP00000029144 | P26772 | Hspe1 | 10116.ENSRNOP00000020066 | 476 |
| Q9ER34 | Aco2 | 10116.ENSRNOP00000029144 | P13803 | Etfa | 10116.ENSRNOP00000020544 | 425 |
| Q9ER34 | Aco2 | 10116.ENSRNOP00000029144 | Q6P6V0 | Gpi | 10116.ENSRNOP00000029515 | 430 |
| Q9ER34 | Aco2 | 10116.ENSRNOP00000029144 | P30839 | Aldh3a2 | 10116.ENSRNOP00000061762 | 471 |
| Q9ER34 | Aco2 | 10116.ENSRNOP00000029144 | P04636 | Mdh2 | 10116.ENSRNOP00000001958 | 890 |
| Q9ER34 | Aco2 | 10116.ENSRNOP00000029144 | Q9JLJ3 | Aldh9a1 | 10116.ENSRNOP00000005611 | 557 |
| P51886 | Lum | 10116.ENSRNOP00000006109 | Q08163 | Cap1 | 10116.ENSRNOP00000018711 | 489 |
| P51886 | Lum | 10116.ENSRNOP00000006109 | P49134 | Itgb1 | 10116.ENSRNOP00000014785 | 941 |
| P51886 | Lum | 10116.ENSRNOP00000006109 | Q5RJR8 | Lrrc59 | 10116.ENSRNOP00000004941 | 423 |
| P51886 | Lum | 10116.ENSRNOP00000006109 | Q01129 | Dcn | 10116.ENSRNOP00000006070 | 938 |
| P51886 | Lum | 10116.ENSRNOP00000006109 | Q9EQP5 | Prelp | 10116.ENSRNOP00000004241 | 905 |
| P51886 | Lum | 10116.ENSRNOP00000006109 | Q66HD0 | Hsp90b1 | 10116.ENSRNOP00000034846 | 659 |
| Q66HD0 | Hsp90b1 | 10116.ENSRNOP00000034846 | Q01129 | Dcn | 10116.ENSRNOP00000006070 | 655 |
| Q66HD0 | Hsp90b1 | 10116.ENSRNOP00000034846 | Q62920 | Pdlim5 | 10116.ENSRNOP00000022387 | 703 |
| Q66HD0 | Hsp90b1 | 10116.ENSRNOP00000034846 | P85834 | Tufm | 10116.ENSRNOP00000025203 | 478 |
| Q66HD0 | Hsp90b1 | 10116.ENSRNOP00000034846 | P41350 | Cav1 | 10116.ENSRNOP00000009253 | 539 |
| Q66HD0 | Hsp90b1 | 10116.ENSRNOP00000034846 | P50399 | Gdi2 | 10116.ENSRNOP00000024952 | 471 |
| Q66HD0 | Hsp90b1 | 10116.ENSRNOP00000034846 | Q63081 | Pdia6 | 10116.ENSRNOP00000064632 | 994 |
| Q66HD0 | Hsp90b1 | 10116.ENSRNOP00000034846 | Q9Z269 | Vapb | 10116.ENSRNOP00000007554 | 427 |
| Q66HD0 | Hsp90b1 | 10116.ENSRNOP00000034846 | Q8CFN2 | Cdc42 | 10116.ENSRNOP00000030928 | 427 |
| Q66HD0 | Hsp90b1 | 10116.ENSRNOP00000034846 | Q6P6V0 | Gpi | 10116.ENSRNOP00000029515 | 515 |
| Q66HD0 | Hsp90b1 | 10116.ENSRNOP00000034846 | Q68FQ0 | Cct5 | 10116.ENSRNOP00000015886 | 757 |
| Q66HD0 | Hsp90b1 | 10116.ENSRNOP00000034846 | P04797 | Gapdh | 10116.ENSRNOP00000040878 | 660 |
| Q66HD0 | Hsp90b1 | 10116.ENSRNOP00000034846 | Q62658 | Fkbp1a | 10116.ENSRNOP00000012608 | 581 |
| Q66HD0 | Hsp90b1 | 10116.ENSRNOP00000034846 | Q68FR6 | Eef1g | 10116.ENSRNOP00000027305 | 528 |
| Q66HD0 | Hsp90b1 | 10116.ENSRNOP00000034846 | P13383 | Ncl | 10116.ENSRNOP00000024712 | 753 |
| Q66HD0 | Hsp90b1 | 10116.ENSRNOP00000034846 | P10111 | Ppia | 10116.ENSRNOP00000009407 | 433 |
| Q66HD0 | Hsp90b1 | 10116.ENSRNOP00000034846 | P80067 | Ctsc | 10116.ENSRNOP00000022342 | 687 |
| Q66HD0 | Hsp90b1 | 10116.ENSRNOP00000034846 | Q5I0H9 | Pdia5 | 10116.ENSRNOP00000059945 | 517 |
| Q66HD0 | Hsp90b1 | 10116.ENSRNOP00000034846 | P26772 | Hspe1 | 10116.ENSRNOP00000020066 | 889 |
| Q66HD0 | Hsp90b1 | 10116.ENSRNOP00000034846 | Q7TPB1 | Cct4 | 10116.ENSRNOP00000012847 | 802 |
| Q66HD0 | Hsp90b1 | 10116.ENSRNOP00000034846 | Q63538 | Mapk12 | 10116.ENSRNOP00000046455 | 456 |
| Q66HD0 | Hsp90b1 | 10116.ENSRNOP00000034846 | Q9Z1Z9 | Pdlim7 | 10116.ENSRNOP00000018899 | 703 |
| Q66HD0 | Hsp90b1 | 10116.ENSRNOP00000034846 | G3V9R8 | Hnrnpc | 10116.ENSRNOP00000057257 | 458 |
| Q66HD0 | Hsp90b1 | 10116.ENSRNOP00000034846 | P60711 | Actb | 10116.ENSRNOP00000044296 | 529 |
| Q66HD0 | Hsp90b1 | 10116.ENSRNOP00000034846 | P63259 | Actg1 | 10116.ENSRNOP00000044296 | 529 |
| Q66HD0 | Hsp90b1 | 10116.ENSRNOP00000034846 | Q9EQP5 | Prelp | 10116.ENSRNOP00000004241 | 660 |
| Q66HD0 | Hsp90b1 | 10116.ENSRNOP00000034846 | Q63525 | Nudc | 10116.ENSRNOP00000009933 | 544 |
| Q66HD0 | Hsp90b1 | 10116.ENSRNOP00000034846 | Q6DGG0 | Ppid | 10116.ENSRNOP00000035797 | 937 |
| Q01129 | Dcn | 10116.ENSRNOP00000006070 | Q08163 | Cap1 | 10116.ENSRNOP00000018711 | 489 |
| Q01129 | Dcn | 10116.ENSRNOP00000006070 | Q62920 | Pdlim5 | 10116.ENSRNOP00000022387 | 541 |
| Q01129 | Dcn | 10116.ENSRNOP00000006070 | P31720 | C1qa | 10116.ENSRNOP00000017385 | 594 |
| Q01129 | Dcn | 10116.ENSRNOP00000006070 | P23965 | Eci1 | 10116.ENSRNOP00000011784 | 503 |
| P36953 | Afm | 10116.ENSRNOP00000057275 | Q64591 | Decr1 | 10116.ENSRNOP00000011330 | 416 |
| P36953 | Afm | 10116.ENSRNOP00000057275 | P06866 | Hp | 10116.ENSRNOP00000049003 | 424 |
| P36953 | Afm | 10116.ENSRNOP00000057275 | P08699 | Lgals3 | 10116.ENSRNOP00000014216 | 606 |
| P36953 | Afm | 10116.ENSRNOP00000057275 | Q64240 | Ambp | 10116.ENSRNOP00000009248 | 442 |
| P04636 | Mdh2 | 10116.ENSRNOP00000001958 | P04797 | Gapdh | 10116.ENSRNOP00000040878 | 523 |
| P04636 | Mdh2 | 10116.ENSRNOP00000001958 | P25113 | Pgam1 | 10116.ENSRNOP00000065690 | 561 |
| P04636 | Mdh2 | 10116.ENSRNOP00000001958 | Q497B0 | Nit2 | 10116.ENSRNOP00000034144 | 800 |
| P04636 | Mdh2 | 10116.ENSRNOP00000001958 | P21571 | Atp5j | 10116.ENSRNOP00000002116 | 403 |
| P04636 | Mdh2 | 10116.ENSRNOP00000001958 | Q6P6R2 | Dld | 10116.ENSRNOP00000008980 | 867 |
| P04636 | Mdh2 | 10116.ENSRNOP00000001958 | P30839 | Aldh3a2 | 10116.ENSRNOP00000061762 | 616 |
| P04636 | Mdh2 | 10116.ENSRNOP00000001958 | P35571 | Gpd2 | 10116.ENSRNOP00000043749 | 522 |
| P04636 | Mdh2 | 10116.ENSRNOP00000001958 | P08461 | Dlat | 10116.ENSRNOP00000032890 | 679 |
| P04636 | Mdh2 | 10116.ENSRNOP00000001958 | P41565 | Idh3g | 10116.ENSRNOP00000053220 | 753 |
| P04636 | Mdh2 | 10116.ENSRNOP00000001958 | P25409 | Gpt | 10116.ENSRNOP00000044411 | 678 |
| P04636 | Mdh2 | 10116.ENSRNOP00000001958 | O88989 | Mdh1 | 10116.ENSRNOP00000011429 | 986 |
| P04636 | Mdh2 | 10116.ENSRNOP00000001958 | Q68FU3 | Etfb | 10116.ENSRNOP00000024083 | 451 |
| P04636 | Mdh2 | 10116.ENSRNOP00000001958 | P13697 | Me1 | 10116.ENSRNOP00000013244 | 979 |
| P04636 | Mdh2 | 10116.ENSRNOP00000001958 | P13803 | Etfa | 10116.ENSRNOP00000020544 | 489 |
| P04636 | Mdh2 | 10116.ENSRNOP00000001958 | P00507 | Got2 | 10116.ENSRNOP00000015956 | 984 |
| P04636 | Mdh2 | 10116.ENSRNOP00000001958 | Q9JLJ3 | Aldh9a1 | 10116.ENSRNOP00000005611 | 602 |
| P04636 | Mdh2 | 10116.ENSRNOP00000001958 | P28037 | Aldh1l1 | 10116.ENSRNOP00000065882 | 652 |
| P04636 | Mdh2 | 10116.ENSRNOP00000001958 | Q8K4D8 | Aldh1a3 | 10116.ENSRNOP00000045261 | 608 |
| P04636 | Mdh2 | 10116.ENSRNOP00000001958 | Q9Z2L0 | Vdac1 | 10116.ENSRNOP00000008477 | 743 |
| P04636 | Mdh2 | 10116.ENSRNOP00000001958 | P48500 | Tpi1 | 10116.ENSRNOP00000067409 | 795 |
| P04636 | Mdh2 | 10116.ENSRNOP00000001958 | Q6P6V0 | Gpi | 10116.ENSRNOP00000029515 | 700 |
| P04636 | Mdh2 | 10116.ENSRNOP00000001958 | P08503 | Acadm | 10116.ENSRNOP00000013238 | 458 |
| P04636 | Mdh2 | 10116.ENSRNOP00000001958 | P41562 | Idh1 | 10116.ENSRNOP00000020322 | 889 |
| P04636 | Mdh2 | 10116.ENSRNOP00000001958 | P49432 | Pdhb | 10116.ENSRNOP00000010545 | 820 |
| P04636 | Mdh2 | 10116.ENSRNOP00000001958 | Q05962 | Slc25a4 | 10116.ENSRNOP00000014704 | 414 |
| P04636 | Mdh2 | 10116.ENSRNOP00000001958 | P26772 | Hspe1 | 10116.ENSRNOP00000020066 | 454 |
| P04636 | Mdh2 | 10116.ENSRNOP00000001958 | Q68FQ0 | Cct5 | 10116.ENSRNOP00000015886 | 545 |
| P04636 | Mdh2 | 10116.ENSRNOP00000001958 | Q7TPB1 | Cct4 | 10116.ENSRNOP00000012847 | 535 |
| P04636 | Mdh2 | 10116.ENSRNOP00000001958 | Q9EQS0 | Taldo1 | 10116.ENSRNOP00000024863 | 500 |
| P04636 | Mdh2 | 10116.ENSRNOP00000001958 | P31399 | Atp5h | 10116.ENSRNOP00000004836 | 677 |
| P04636 | Mdh2 | 10116.ENSRNOP00000001958 | P85834 | Tufm | 10116.ENSRNOP00000025203 | 429 |
| P04636 | Mdh2 | 10116.ENSRNOP00000001958 | P35738 | Bckdhb | 10116.ENSRNOP00000013249 | 425 |
| P04636 | Mdh2 | 10116.ENSRNOP00000001958 | O35077 | Gpd1 | 10116.ENSRNOP00000026200 | 538 |
| P04636 | Mdh2 | 10116.ENSRNOP00000001958 | P05708 | Hk1 | 10116.ENSRNOP00000066611 | 506 |
| Q9Z2L0 | Vdac1 | 10116.ENSRNOP00000008477 | Q6AYZ1 | Tuba1c | 10116.ENSRNOP00000020932 | 471 |
| Q9Z2L0 | Vdac1 | 10116.ENSRNOP00000008477 | Q05962 | Slc25a4 | 10116.ENSRNOP00000014704 | 886 |
| Q9Z2L0 | Vdac1 | 10116.ENSRNOP00000008477 | P05708 | Hk1 | 10116.ENSRNOP00000066611 | 468 |
| Q9Z2L0 | Vdac1 | 10116.ENSRNOP00000008477 | F1MA98 | Tpr | 10116.ENSRNOP00000062172 | 540 |
| Q9Z2L0 | Vdac1 | 10116.ENSRNOP00000008477 | P04797 | Gapdh | 10116.ENSRNOP00000040878 | 583 |
| Q9Z2L0 | Vdac1 | 10116.ENSRNOP00000008477 | P55314 | C8b | 10116.ENSRNOP00000010100 | 540 |
| Q9Z2L0 | Vdac1 | 10116.ENSRNOP00000008477 | Q6DGG0 | Ppid | 10116.ENSRNOP00000035797 | 565 |
| Q9Z2L0 | Vdac1 | 10116.ENSRNOP00000008477 | P10111 | Ppia | 10116.ENSRNOP00000009407 | 758 |
| Q9Z2L0 | Vdac1 | 10116.ENSRNOP00000008477 | Q6P6R2 | Dld | 10116.ENSRNOP00000008980 | 484 |
| Q9Z2L0 | Vdac1 | 10116.ENSRNOP00000008477 | O88767 | Park7 | 10116.ENSRNOP00000024711 | 619 |
| P04797 | Gapdh | 10116.ENSRNOP00000040878 | P49432 | Pdhb | 10116.ENSRNOP00000010545 | 615 |
| P04797 | Gapdh | 10116.ENSRNOP00000040878 | P15337 | Creb1 | 10116.ENSRNOP00000018326 | 803 |
| P04797 | Gapdh | 10116.ENSRNOP00000040878 | P01015 | Agt | 10116.ENSRNOP00000024917 | 805 |
| P04797 | Gapdh | 10116.ENSRNOP00000040878 | P08461 | Dlat | 10116.ENSRNOP00000032890 | 546 |
| P04797 | Gapdh | 10116.ENSRNOP00000040878 | Q7TPB1 | Cct4 | 10116.ENSRNOP00000012847 | 403 |
| P04797 | Gapdh | 10116.ENSRNOP00000040878 | P50137 | Tkt | 10116.ENSRNOP00000021862 | 833 |
| P04797 | Gapdh | 10116.ENSRNOP00000040878 | P85834 | Tufm | 10116.ENSRNOP00000025203 | 429 |
| P04797 | Gapdh | 10116.ENSRNOP00000040878 | P60711 | Actb | 10116.ENSRNOP00000044296 | 906 |
| P04797 | Gapdh | 10116.ENSRNOP00000040878 | P63259 | Actg1 | 10116.ENSRNOP00000044296 | 906 |
| P04797 | Gapdh | 10116.ENSRNOP00000040878 | O88767 | Park7 | 10116.ENSRNOP00000024711 | 413 |
| P04797 | Gapdh | 10116.ENSRNOP00000040878 | P04041 | Gpx1 | 10116.ENSRNOP00000066577 | 627 |
| P04797 | Gapdh | 10116.ENSRNOP00000040878 | Q6P6V0 | Gpi | 10116.ENSRNOP00000029515 | 867 |
| P04797 | Gapdh | 10116.ENSRNOP00000040878 | O08651 | Phgdh | 10116.ENSRNOP00000053019 | 666 |
| P04797 | Gapdh | 10116.ENSRNOP00000040878 | Q6AYZ1 | Tuba1c | 10116.ENSRNOP00000020932 | 504 |
| P04797 | Gapdh | 10116.ENSRNOP00000040878 | P41562 | Idh1 | 10116.ENSRNOP00000020322 | 481 |
| P04797 | Gapdh | 10116.ENSRNOP00000040878 | P48500 | Tpi1 | 10116.ENSRNOP00000067409 | 987 |
| P04797 | Gapdh | 10116.ENSRNOP00000040878 | Q8CFN2 | Cdc42 | 10116.ENSRNOP00000030928 | 601 |
| P04797 | Gapdh | 10116.ENSRNOP00000040878 | O88989 | Mdh1 | 10116.ENSRNOP00000011429 | 488 |
| P04797 | Gapdh | 10116.ENSRNOP00000040878 | P10111 | Ppia | 10116.ENSRNOP00000009407 | 685 |
| P04797 | Gapdh | 10116.ENSRNOP00000040878 | P35738 | Bckdhb | 10116.ENSRNOP00000013249 | 556 |
| P04797 | Gapdh | 10116.ENSRNOP00000040878 | P14668 | Anxa5 | 10116.ENSRNOP00000019552 | 462 |
| P04797 | Gapdh | 10116.ENSRNOP00000040878 | Q9EQS0 | Taldo1 | 10116.ENSRNOP00000024863 | 462 |
| P04797 | Gapdh | 10116.ENSRNOP00000040878 | P08699 | Lgals3 | 10116.ENSRNOP00000014216 | 661 |
| P04797 | Gapdh | 10116.ENSRNOP00000040878 | P25113 | Pgam1 | 10116.ENSRNOP00000065690 | 866 |
| P04797 | Gapdh | 10116.ENSRNOP00000040878 | P62832 | Rpl23 | 10116.ENSRNOP00000005471 | 437 |
| P04797 | Gapdh | 10116.ENSRNOP00000040878 | P35427 | Rpl13a | 10116.ENSRNOP00000027976 | 932 |
| P04797 | Gapdh | 10116.ENSRNOP00000040878 | P06762 | Hmox1 | 10116.ENSRNOP00000019192 | 639 |
| P04797 | Gapdh | 10116.ENSRNOP00000040878 | P14141 | Ca3 | 10116.ENSRNOP00000014177 | 459 |
| P04797 | Gapdh | 10116.ENSRNOP00000040878 | O35077 | Gpd1 | 10116.ENSRNOP00000026200 | 564 |
| P14668 | Anxa5 | 10116.ENSRNOP00000019552 | Q6B345 | S100a11 | 10116.ENSRNOP00000013393 | 402 |
| P14668 | Anxa5 | 10116.ENSRNOP00000019552 | P11762 | Lgals1 | 10116.ENSRNOP00000013538 | 801 |
| P14668 | Anxa5 | 10116.ENSRNOP00000019552 | P01015 | Agt | 10116.ENSRNOP00000024917 | 690 |
| P50137 | Tkt | 10116.ENSRNOP00000021862 | Q6P6R2 | Dld | 10116.ENSRNOP00000008980 | 402 |
| P50137 | Tkt | 10116.ENSRNOP00000021862 | Q9JLJ3 | Aldh9a1 | 10116.ENSRNOP00000005611 | 493 |
| P50137 | Tkt | 10116.ENSRNOP00000021862 | Q6P6V0 | Gpi | 10116.ENSRNOP00000029515 | 977 |
| P50137 | Tkt | 10116.ENSRNOP00000021862 | P49432 | Pdhb | 10116.ENSRNOP00000010545 | 912 |
| P50137 | Tkt | 10116.ENSRNOP00000021862 | Q8K4D8 | Aldh1a3 | 10116.ENSRNOP00000045261 | 493 |
| P50137 | Tkt | 10116.ENSRNOP00000021862 | P05708 | Hk1 | 10116.ENSRNOP00000066611 | 871 |
| P50137 | Tkt | 10116.ENSRNOP00000021862 | P25113 | Pgam1 | 10116.ENSRNOP00000065690 | 860 |
| P50137 | Tkt | 10116.ENSRNOP00000021862 | Q9EQS0 | Taldo1 | 10116.ENSRNOP00000024863 | 996 |
| P50137 | Tkt | 10116.ENSRNOP00000021862 | P60892 | Prps1 | 10116.ENSRNOP00000029405 | 916 |
| P50137 | Tkt | 10116.ENSRNOP00000021862 | P41562 | Idh1 | 10116.ENSRNOP00000020322 | 448 |
| P50137 | Tkt | 10116.ENSRNOP00000021862 | P08461 | Dlat | 10116.ENSRNOP00000032890 | 542 |
| P50137 | Tkt | 10116.ENSRNOP00000021862 | P48500 | Tpi1 | 10116.ENSRNOP00000067409 | 866 |
| P50137 | Tkt | 10116.ENSRNOP00000021862 | P35738 | Bckdhb | 10116.ENSRNOP00000013249 | 573 |
| F1LMY4 | Ryr1 | 10116.ENSRNOP00000027893 | P19633 | Casq1 | 10116.ENSRNOP00000009344 | 683 |
| F1LMY4 | Ryr1 | 10116.ENSRNOP00000027893 | Q62658 | Fkbp1a | 10116.ENSRNOP00000012608 | 977 |
| P09006 | Serpina3n | 10116.ENSRNOP00000014073 | P00697 | Lyz1 | 10116.ENSRNOP00000007747 | 449 |
| P09006 | Serpina3n | 10116.ENSRNOP00000014073 | Q64240 | Ambp | 10116.ENSRNOP00000009248 | 431 |
| P09006 | Serpina3n | 10116.ENSRNOP00000014073 | P06866 | Hp | 10116.ENSRNOP00000049003 | 442 |
| P09006 | Serpina3n | 10116.ENSRNOP00000014073 | P08699 | Lgals3 | 10116.ENSRNOP00000014216 | 610 |
| P50399 | Gdi2 | 10116.ENSRNOP00000024952 | Q7M0E3 | Dstn | 10116.ENSRNOP00000007794 | 518 |
| P50399 | Gdi2 | 10116.ENSRNOP00000024952 | Q8CFN2 | Cdc42 | 10116.ENSRNOP00000030928 | 942 |
| P50399 | Gdi2 | 10116.ENSRNOP00000024952 | P50398 | Gdi1 | 10116.ENSRNOP00000053135 | 907 |
| P50399 | Gdi2 | 10116.ENSRNOP00000024952 | Q08163 | Cap1 | 10116.ENSRNOP00000018711 | 574 |
| P50399 | Gdi2 | 10116.ENSRNOP00000024952 | Q91Y81 | Sept2 | 10116.ENSRNOP00000024261 | 476 |
| Q05962 | Slc25a4 | 10116.ENSRNOP00000014704 | Q68FR6 | Eef1g | 10116.ENSRNOP00000027305 | 509 |
| Q05962 | Slc25a4 | 10116.ENSRNOP00000014704 | P62914 | Rpl11 | 10116.ENSRNOP00000037110 | 454 |
| Q05962 | Slc25a4 | 10116.ENSRNOP00000014704 | P50878 | Rpl4 | 10116.ENSRNOP00000013462 | 434 |
| P14141 | Ca3 | 10116.ENSRNOP00000014177 | P08699 | Lgals3 | 10116.ENSRNOP00000014216 | 537 |
| Q9Z1X1 | Esyt1 | 10116.ENSRNOP00000006119 | Q4KMA2 | Rad23b | 10116.ENSRNOP00000021629 | 466 |
| Q9Z1X1 | Esyt1 | 10116.ENSRNOP00000006119 | P18445 | Rpl27a | 10116.ENSRNOP00000019247 | 474 |
| P62260 | Ywhae | 10116.ENSRNOP00000007100 | P50878 | Rpl4 | 10116.ENSRNOP00000013462 | 445 |
| P62260 | Ywhae | 10116.ENSRNOP00000007100 | P24155 | Thop1 | 10116.ENSRNOP00000027045 | 514 |
| P62260 | Ywhae | 10116.ENSRNOP00000007100 | Q6AYH5 | Dctn2 | 10116.ENSRNOP00000008120 | 902 |
| P62260 | Ywhae | 10116.ENSRNOP00000007100 | P49134 | Itgb1 | 10116.ENSRNOP00000014785 | 453 |
| P62260 | Ywhae | 10116.ENSRNOP00000007100 | Q63258 | Itga7 | 10116.ENSRNOP00000058370 | 524 |
| P62260 | Ywhae | 10116.ENSRNOP00000007100 | P68255 | Ywhaq | 10116.ENSRNOP00000011501 | 812 |
| P62260 | Ywhae | 10116.ENSRNOP00000007100 | Q68FR6 | Eef1g | 10116.ENSRNOP00000027305 | 703 |
| P62260 | Ywhae | 10116.ENSRNOP00000007100 | P61983 | Ywhag | 10116.ENSRNOP00000001954 | 978 |
| P62260 | Ywhae | 10116.ENSRNOP00000007100 | Q63538 | Mapk12 | 10116.ENSRNOP00000046455 | 500 |
| P62260 | Ywhae | 10116.ENSRNOP00000007100 | P60711 | Actb | 10116.ENSRNOP00000044296 | 466 |
| P62260 | Ywhae | 10116.ENSRNOP00000007100 | P63259 | Actg1 | 10116.ENSRNOP00000044296 | 466 |
| Q9EQP5 | Prelp | 10116.ENSRNOP00000004241 | Q08163 | Cap1 | 10116.ENSRNOP00000018711 | 489 |
| P60711 | Actb | 10116.ENSRNOP00000044296 | P10111 | Ppia | 10116.ENSRNOP00000009407 | 725 |
| P60711 | Actb | 10116.ENSRNOP00000044296 | P20761 | Igh-1a | 10116.ENSRNOP00000044037 | 900 |
| P60711 | Actb | 10116.ENSRNOP00000044296 | P02625 | Pvalb | 10116.ENSRNOP00000058825 | 543 |
| P60711 | Actb | 10116.ENSRNOP00000044296 | Q6AYH5 | Dctn2 | 10116.ENSRNOP00000008120 | 594 |
| P60711 | Actb | 10116.ENSRNOP00000044296 | P13832 | Rlc-a | 10116.ENSRNOP00000021048 | 866 |
| P60711 | Actb | 10116.ENSRNOP00000044296 | P18588 | Mx1 | 10116.ENSRNOP00000043001 | 635 |
| P60711 | Actb | 10116.ENSRNOP00000044296 | P61983 | Ywhag | 10116.ENSRNOP00000001954 | 433 |
| P60711 | Actb | 10116.ENSRNOP00000044296 | P20759 | ENSRNOG00000030332 | 10116.ENSRNOP00000006975 | 909 |
| P60711 | Actb | 10116.ENSRNOP00000044296 | P35427 | Rpl13a | 10116.ENSRNOP00000027976 | 621 |
| P60711 | Actb | 10116.ENSRNOP00000044296 | Q08163 | Cap1 | 10116.ENSRNOP00000018711 | 701 |
| P60711 | Actb | 10116.ENSRNOP00000044296 | Q63538 | Mapk12 | 10116.ENSRNOP00000046455 | 458 |
| P60711 | Actb | 10116.ENSRNOP00000044296 | Q7TPB1 | Cct4 | 10116.ENSRNOP00000012847 | 966 |
| P60711 | Actb | 10116.ENSRNOP00000044296 | Q8CFN2 | Cdc42 | 10116.ENSRNOP00000030928 | 912 |
| P60711 | Actb | 10116.ENSRNOP00000044296 | Q68FQ0 | Cct5 | 10116.ENSRNOP00000015886 | 931 |
| P60711 | Actb | 10116.ENSRNOP00000044296 | Q5XFX0 | Tagln2 | 10116.ENSRNOP00000011208 | 485 |
| P60711 | Actb | 10116.ENSRNOP00000044296 | Q7M0E3 | Dstn | 10116.ENSRNOP00000007794 | 817 |
| P60711 | Actb | 10116.ENSRNOP00000044296 | P41350 | Cav1 | 10116.ENSRNOP00000009253 | 611 |
| P60711 | Actb | 10116.ENSRNOP00000044296 | Q63258 | Itga7 | 10116.ENSRNOP00000058370 | 803 |
| P60711 | Actb | 10116.ENSRNOP00000044296 | Q6AYZ1 | Tuba1c | 10116.ENSRNOP00000020932 | 958 |
| P60711 | Actb | 10116.ENSRNOP00000044296 | P49134 | Itgb1 | 10116.ENSRNOP00000014785 | 922 |
| P06866 | Hp | 10116.ENSRNOP00000049003 | P11762 | Lgals1 | 10116.ENSRNOP00000013538 | 688 |
| P06866 | Hp | 10116.ENSRNOP00000049003 | P08699 | Lgals3 | 10116.ENSRNOP00000014216 | 688 |
| P06866 | Hp | 10116.ENSRNOP00000049003 | P25409 | Gpt | 10116.ENSRNOP00000044411 | 468 |
| P10111 | Ppia | 10116.ENSRNOP00000009407 | P50878 | Rpl4 | 10116.ENSRNOP00000013462 | 413 |
| P10111 | Ppia | 10116.ENSRNOP00000009407 | Q63538 | Mapk12 | 10116.ENSRNOP00000046455 | 508 |
| P10111 | Ppia | 10116.ENSRNOP00000009407 | P63281 | Ube2i | 10116.ENSRNOP00000024406 | 705 |
| P10111 | Ppia | 10116.ENSRNOP00000009407 | P35427 | Rpl13a | 10116.ENSRNOP00000027976 | 622 |
| P10111 | Ppia | 10116.ENSRNOP00000009407 | P63259 | Actg1 | 10116.ENSRNOP00000044296 | 725 |
| P10111 | Ppia | 10116.ENSRNOP00000009407 | Q5XFX0 | Tagln2 | 10116.ENSRNOP00000011208 | 418 |
| P10111 | Ppia | 10116.ENSRNOP00000009407 | Q62658 | Fkbp1a | 10116.ENSRNOP00000012608 | 454 |
| Q63081 | Pdia6 | 10116.ENSRNOP00000064632 | Q641Y0 | Ddost | 10116.ENSRNOP00000062365 | 470 |
| Q63081 | Pdia6 | 10116.ENSRNOP00000064632 | O35244 | Prdx6 | 10116.ENSRNOP00000030323 | 610 |
| Q63081 | Pdia6 | 10116.ENSRNOP00000064632 | Q7TPB1 | Cct4 | 10116.ENSRNOP00000012847 | 513 |
| Q63081 | Pdia6 | 10116.ENSRNOP00000064632 | P26772 | Hspe1 | 10116.ENSRNOP00000020066 | 462 |
| Q63081 | Pdia6 | 10116.ENSRNOP00000064632 | Q6P6R2 | Dld | 10116.ENSRNOP00000008980 | 466 |
| Q63081 | Pdia6 | 10116.ENSRNOP00000064632 | Q68FQ0 | Cct5 | 10116.ENSRNOP00000015886 | 451 |
| Q63081 | Pdia6 | 10116.ENSRNOP00000064632 | P04041 | Gpx1 | 10116.ENSRNOP00000066577 | 420 |
| Q63081 | Pdia6 | 10116.ENSRNOP00000064632 | P61983 | Ywhag | 10116.ENSRNOP00000001954 | 453 |
| P02625 | Pvalb | 10116.ENSRNOP00000058825 | P63259 | Actg1 | 10116.ENSRNOP00000044296 | 543 |
| P02625 | Pvalb | 10116.ENSRNOP00000058825 | P11505 | Atp2b1 | 10116.ENSRNOP00000005491 | 427 |
| P15684 | Anpep | 10116.ENSRNOP00000020002 | P01015 | Agt | 10116.ENSRNOP00000024917 | 920 |
| P15684 | Anpep | 10116.ENSRNOP00000020002 | P00507 | Got2 | 10116.ENSRNOP00000015956 | 800 |
| P15684 | Anpep | 10116.ENSRNOP00000020002 | P61983 | Ywhag | 10116.ENSRNOP00000001954 | 404 |
| P15684 | Anpep | 10116.ENSRNOP00000020002 | P68255 | Ywhaq | 10116.ENSRNOP00000011501 | 472 |
| P15684 | Anpep | 10116.ENSRNOP00000020002 | Q68FR6 | Eef1g | 10116.ENSRNOP00000027305 | 409 |
| P15684 | Anpep | 10116.ENSRNOP00000020002 | P49134 | Itgb1 | 10116.ENSRNOP00000014785 | 516 |
| P15684 | Anpep | 10116.ENSRNOP00000020002 | P08699 | Lgals3 | 10116.ENSRNOP00000014216 | 712 |
| P48500 | Tpi1 | 10116.ENSRNOP00000067409 | Q4KMA2 | Rad23b | 10116.ENSRNOP00000021629 | 402 |
| P48500 | Tpi1 | 10116.ENSRNOP00000067409 | P31399 | Atp5h | 10116.ENSRNOP00000004836 | 411 |
| P48500 | Tpi1 | 10116.ENSRNOP00000067409 | O88989 | Mdh1 | 10116.ENSRNOP00000011429 | 708 |
| P48500 | Tpi1 | 10116.ENSRNOP00000067409 | P49432 | Pdhb | 10116.ENSRNOP00000010545 | 617 |
| P48500 | Tpi1 | 10116.ENSRNOP00000067409 | Q6P6V0 | Gpi | 10116.ENSRNOP00000029515 | 998 |
| P48500 | Tpi1 | 10116.ENSRNOP00000067409 | Q9EQS0 | Taldo1 | 10116.ENSRNOP00000024863 | 888 |
| P48500 | Tpi1 | 10116.ENSRNOP00000067409 | P35571 | Gpd2 | 10116.ENSRNOP00000043749 | 410 |
| P48500 | Tpi1 | 10116.ENSRNOP00000067409 | Q9JLJ3 | Aldh9a1 | 10116.ENSRNOP00000005611 | 511 |
| P48500 | Tpi1 | 10116.ENSRNOP00000067409 | P62278 | Rps13 | 10116.ENSRNOP00000036690 | 628 |
| P48500 | Tpi1 | 10116.ENSRNOP00000067409 | P28037 | Aldh1l1 | 10116.ENSRNOP00000065882 | 494 |
| P48500 | Tpi1 | 10116.ENSRNOP00000067409 | P30839 | Aldh3a2 | 10116.ENSRNOP00000061762 | 581 |
| P48500 | Tpi1 | 10116.ENSRNOP00000067409 | O35077 | Gpd1 | 10116.ENSRNOP00000026200 | 401 |
| P48500 | Tpi1 | 10116.ENSRNOP00000067409 | Q8K4D8 | Aldh1a3 | 10116.ENSRNOP00000045261 | 511 |
| P48500 | Tpi1 | 10116.ENSRNOP00000067409 | P05708 | Hk1 | 10116.ENSRNOP00000066611 | 448 |
| P48500 | Tpi1 | 10116.ENSRNOP00000067409 | P00507 | Got2 | 10116.ENSRNOP00000015956 | 571 |
| P48500 | Tpi1 | 10116.ENSRNOP00000067409 | O88767 | Park7 | 10116.ENSRNOP00000024711 | 716 |
| P48500 | Tpi1 | 10116.ENSRNOP00000067409 | P41565 | Idh3g | 10116.ENSRNOP00000053220 | 519 |
| P48500 | Tpi1 | 10116.ENSRNOP00000067409 | P25113 | Pgam1 | 10116.ENSRNOP00000065690 | 975 |
| P15800 | Lamb2 | 10116.ENSRNOP00000065052 | P49134 | Itgb1 | 10116.ENSRNOP00000014785 | 939 |
| P15800 | Lamb2 | 10116.ENSRNOP00000065052 | Q8CFN2 | Cdc42 | 10116.ENSRNOP00000030928 | 674 |
| P15800 | Lamb2 | 10116.ENSRNOP00000065052 | P11530 | Dmd | 10116.ENSRNOP00000029969 | 719 |
| P15800 | Lamb2 | 10116.ENSRNOP00000065052 | Q63258 | Itga7 | 10116.ENSRNOP00000058370 | 898 |
| Q63416 | Itih3 | 10116.ENSRNOP00000023984 | P08699 | Lgals3 | 10116.ENSRNOP00000014216 | 425 |
| Q63416 | Itih3 | 10116.ENSRNOP00000023984 | P11762 | Lgals1 | 10116.ENSRNOP00000013538 | 425 |
| Q63416 | Itih3 | 10116.ENSRNOP00000023984 | P17220 | Psma2 | 10116.ENSRNOP00000066950 | 468 |
| Q63416 | Itih3 | 10116.ENSRNOP00000023984 | Q64240 | Ambp | 10116.ENSRNOP00000009248 | 727 |
| P02680 | Fgg | 10116.ENSRNOP00000032735 | P49134 | Itgb1 | 10116.ENSRNOP00000014785 | 612 |
| P02680 | Fgg | 10116.ENSRNOP00000032735 | Q64240 | Ambp | 10116.ENSRNOP00000009248 | 815 |
| Q6P6R2 | Dld | 10116.ENSRNOP00000008980 | P26772 | Hspe1 | 10116.ENSRNOP00000020066 | 536 |
| Q6P6R2 | Dld | 10116.ENSRNOP00000008980 | Q6AYK6 | Cacybp | 10116.ENSRNOP00000003586 | 554 |
| Q6P6R2 | Dld | 10116.ENSRNOP00000008980 | P04041 | Gpx1 | 10116.ENSRNOP00000066577 | 586 |
| Q6P6R2 | Dld | 10116.ENSRNOP00000008980 | P41565 | Idh3g | 10116.ENSRNOP00000053220 | 973 |
| Q6P6R2 | Dld | 10116.ENSRNOP00000008980 | P35571 | Gpd2 | 10116.ENSRNOP00000043749 | 494 |
| Q6P6R2 | Dld | 10116.ENSRNOP00000008980 | P13803 | Etfa | 10116.ENSRNOP00000020544 | 688 |
| Q6P6R2 | Dld | 10116.ENSRNOP00000008980 | O88989 | Mdh1 | 10116.ENSRNOP00000011429 | 530 |
| Q6P6R2 | Dld | 10116.ENSRNOP00000008980 | O35244 | Prdx6 | 10116.ENSRNOP00000030323 | 445 |
| Q6P6R2 | Dld | 10116.ENSRNOP00000008980 | P17220 | Psma2 | 10116.ENSRNOP00000066950 | 441 |
| Q6P6R2 | Dld | 10116.ENSRNOP00000008980 | Q5I0H9 | Pdia5 | 10116.ENSRNOP00000059945 | 466 |
| Q6P6R2 | Dld | 10116.ENSRNOP00000008980 | P08461 | Dlat | 10116.ENSRNOP00000032890 | 999 |
| Q6P6R2 | Dld | 10116.ENSRNOP00000008980 | P49432 | Pdhb | 10116.ENSRNOP00000010545 | 999 |
| Q6P6R2 | Dld | 10116.ENSRNOP00000008980 | P35738 | Bckdhb | 10116.ENSRNOP00000013249 | 989 |
| Q6P6R2 | Dld | 10116.ENSRNOP00000008980 | P08503 | Acadm | 10116.ENSRNOP00000013238 | 664 |
| Q6P6R2 | Dld | 10116.ENSRNOP00000008980 | Q64611 | Csad | 10116.ENSRNOP00000016205 | 408 |
| Q6P6R2 | Dld | 10116.ENSRNOP00000008980 | P00507 | Got2 | 10116.ENSRNOP00000015956 | 427 |
| Q6P6R2 | Dld | 10116.ENSRNOP00000008980 | P45953 | Acadvl | 10116.ENSRNOP00000024973 | 469 |
| Q6P6R2 | Dld | 10116.ENSRNOP00000008980 | Q68FU3 | Etfb | 10116.ENSRNOP00000024083 | 469 |
| Q6P6R2 | Dld | 10116.ENSRNOP00000008980 | P41562 | Idh1 | 10116.ENSRNOP00000020322 | 623 |
| Q6P6R2 | Dld | 10116.ENSRNOP00000008980 | Q02253 | Aldh6a1 | 10116.ENSRNOP00000015545 | 519 |
| Q6P6R2 | Dld | 10116.ENSRNOP00000008980 | P13697 | Me1 | 10116.ENSRNOP00000013244 | 462 |
| Q6P6R2 | Dld | 10116.ENSRNOP00000008980 | P12007 | Ivd | 10116.ENSRNOP00000013829 | 905 |
| Q6P6R2 | Dld | 10116.ENSRNOP00000008980 | Q64591 | Decr1 | 10116.ENSRNOP00000011330 | 500 |
| P97852 | Hsd17b4 | 10116.ENSRNOP00000021646 | P23965 | Eci1 | 10116.ENSRNOP00000011784 | 738 |
| P97852 | Hsd17b4 | 10116.ENSRNOP00000021646 | P35571 | Gpd2 | 10116.ENSRNOP00000043749 | 476 |
| P97852 | Hsd17b4 | 10116.ENSRNOP00000021646 | P12007 | Ivd | 10116.ENSRNOP00000013829 | 415 |
| P97852 | Hsd17b4 | 10116.ENSRNOP00000021646 | P30839 | Aldh3a2 | 10116.ENSRNOP00000061762 | 460 |
| P97852 | Hsd17b4 | 10116.ENSRNOP00000021646 | O70351 | Hsd17b10 | 10116.ENSRNOP00000043608 | 597 |
| P97852 | Hsd17b4 | 10116.ENSRNOP00000021646 | P45953 | Acadvl | 10116.ENSRNOP00000024973 | 785 |
| P97852 | Hsd17b4 | 10116.ENSRNOP00000021646 | Q6AYG5 | Echdc1 | 10116.ENSRNOP00000015440 | 639 |
| P97852 | Hsd17b4 | 10116.ENSRNOP00000021646 | P41562 | Idh1 | 10116.ENSRNOP00000020322 | 401 |
| P97852 | Hsd17b4 | 10116.ENSRNOP00000021646 | Q64591 | Decr1 | 10116.ENSRNOP00000011330 | 617 |
| P97852 | Hsd17b4 | 10116.ENSRNOP00000021646 | Q9JLJ3 | Aldh9a1 | 10116.ENSRNOP00000005611 | 433 |
| P97852 | Hsd17b4 | 10116.ENSRNOP00000021646 | P08503 | Acadm | 10116.ENSRNOP00000013238 | 844 |
| P97852 | Hsd17b4 | 10116.ENSRNOP00000021646 | Q5XIC0 | Eci2 | 10116.ENSRNOP00000022022 | 885 |
| Q4V8H8 | Ehd2 | 10116.ENSRNOP00000017353 | P41350 | Cav1 | 10116.ENSRNOP00000009253 | 740 |
| Q4V8H8 | Ehd2 | 10116.ENSRNOP00000017353 | Q9EQS0 | Taldo1 | 10116.ENSRNOP00000024863 | 451 |
| P10759 | Ampd1 | 10116.ENSRNOP00000025248 | P19633 | Casq1 | 10116.ENSRNOP00000009344 | 740 |
| Q68FR6 | Eef1g | 10116.ENSRNOP00000027305 | P25113 | Pgam1 | 10116.ENSRNOP00000065690 | 441 |
| Q68FR6 | Eef1g | 10116.ENSRNOP00000027305 | P62914 | Rpl11 | 10116.ENSRNOP00000037110 | 904 |
| Q68FR6 | Eef1g | 10116.ENSRNOP00000027305 | P04041 | Gpx1 | 10116.ENSRNOP00000066577 | 473 |
| Q68FR6 | Eef1g | 10116.ENSRNOP00000027305 | Q5I0G4 | Gars | 10116.ENSRNOP00000014780 | 683 |
| Q68FR6 | Eef1g | 10116.ENSRNOP00000027305 | A0JPJ7 | Ola1 | 10116.ENSRNOP00000026040 | 741 |
| Q68FR6 | Eef1g | 10116.ENSRNOP00000027305 | P50878 | Rpl4 | 10116.ENSRNOP00000013462 | 987 |
| Q68FR6 | Eef1g | 10116.ENSRNOP00000027305 | P41123 | Rpl13 | 10116.ENSRNOP00000020635 | 941 |
| Q68FR6 | Eef1g | 10116.ENSRNOP00000027305 | P60868 | Rps20 | 10116.ENSRNOP00000066077 | 748 |
| Q68FR6 | Eef1g | 10116.ENSRNOP00000027305 | Q63413 | Ddx39b | 10116.ENSRNOP00000001115 | 692 |
| Q68FR6 | Eef1g | 10116.ENSRNOP00000027305 | P23358 | Rpl12 | 10116.ENSRNOP00000041462 | 795 |
| Q68FR6 | Eef1g | 10116.ENSRNOP00000027305 | P61983 | Ywhag | 10116.ENSRNOP00000001954 | 647 |
| Q68FR6 | Eef1g | 10116.ENSRNOP00000027305 | P62752 | Rpl23a | 10116.ENSRNOP00000036391 | 799 |
| Q68FR6 | Eef1g | 10116.ENSRNOP00000027305 | Q04462 | Vars | 10116.ENSRNOP00000001160 | 646 |
| Q68FR6 | Eef1g | 10116.ENSRNOP00000027305 | P35427 | Rpl13a | 10116.ENSRNOP00000027976 | 791 |
| Q68FR6 | Eef1g | 10116.ENSRNOP00000027305 | P21533 | Rpl6 | 10116.ENSRNOP00000051135 | 931 |
| Q68FR6 | Eef1g | 10116.ENSRNOP00000027305 | P68255 | Ywhaq | 10116.ENSRNOP00000011501 | 569 |
| Q68FR6 | Eef1g | 10116.ENSRNOP00000027305 | P62718 | Rpl18a | 10116.ENSRNOP00000025421 | 861 |
| Q68FR6 | Eef1g | 10116.ENSRNOP00000027305 | P18445 | Rpl27a | 10116.ENSRNOP00000019247 | 743 |
| Q68FR6 | Eef1g | 10116.ENSRNOP00000027305 | P62278 | Rps13 | 10116.ENSRNOP00000036690 | 817 |
| Q68FR6 | Eef1g | 10116.ENSRNOP00000027305 | Q68FQ0 | Cct5 | 10116.ENSRNOP00000015886 | 836 |
| Q68FR6 | Eef1g | 10116.ENSRNOP00000027305 | P62832 | Rpl23 | 10116.ENSRNOP00000005471 | 636 |
| Q68FR6 | Eef1g | 10116.ENSRNOP00000027305 | P60892 | Prps1 | 10116.ENSRNOP00000029405 | 652 |
| Q68FR6 | Eef1g | 10116.ENSRNOP00000027305 | Q7TPB1 | Cct4 | 10116.ENSRNOP00000012847 | 495 |
| P45953 | Acadvl | 10116.ENSRNOP00000024973 | P23965 | Eci1 | 10116.ENSRNOP00000011784 | 875 |
| P45953 | Acadvl | 10116.ENSRNOP00000024973 | Q6AYG5 | Echdc1 | 10116.ENSRNOP00000015440 | 824 |
| P45953 | Acadvl | 10116.ENSRNOP00000024973 | Q64591 | Decr1 | 10116.ENSRNOP00000011330 | 597 |
| P45953 | Acadvl | 10116.ENSRNOP00000024973 | P15304 | Lipe | 10116.ENSRNOP00000027911 | 468 |
| P45953 | Acadvl | 10116.ENSRNOP00000024973 | P49432 | Pdhb | 10116.ENSRNOP00000010545 | 520 |
| P45953 | Acadvl | 10116.ENSRNOP00000024973 | Q8K4D8 | Aldh1a3 | 10116.ENSRNOP00000045261 | 498 |
| P45953 | Acadvl | 10116.ENSRNOP00000024973 | P08461 | Dlat | 10116.ENSRNOP00000032890 | 583 |
| P45953 | Acadvl | 10116.ENSRNOP00000024973 | Q5XIC0 | Eci2 | 10116.ENSRNOP00000022022 | 873 |
| P45953 | Acadvl | 10116.ENSRNOP00000024973 | P35738 | Bckdhb | 10116.ENSRNOP00000013249 | 502 |
| P45953 | Acadvl | 10116.ENSRNOP00000024973 | Q499N5 | Acsf2 | 10116.ENSRNOP00000004673 | 702 |
| P45953 | Acadvl | 10116.ENSRNOP00000024973 | P08503 | Acadm | 10116.ENSRNOP00000013238 | 535 |
| P45953 | Acadvl | 10116.ENSRNOP00000024973 | Q68FU3 | Etfb | 10116.ENSRNOP00000024083 | 733 |
| P45953 | Acadvl | 10116.ENSRNOP00000024973 | Q9JLJ3 | Aldh9a1 | 10116.ENSRNOP00000005611 | 567 |
| P45953 | Acadvl | 10116.ENSRNOP00000024973 | P30839 | Aldh3a2 | 10116.ENSRNOP00000061762 | 541 |
| P45953 | Acadvl | 10116.ENSRNOP00000024973 | P13803 | Etfa | 10116.ENSRNOP00000020544 | 750 |
| P45953 | Acadvl | 10116.ENSRNOP00000024973 | P28037 | Aldh1l1 | 10116.ENSRNOP00000065882 | 507 |
| Q6P6V0 | Gpi | 10116.ENSRNOP00000029515 | P25113 | Pgam1 | 10116.ENSRNOP00000065690 | 942 |
| Q6P6V0 | Gpi | 10116.ENSRNOP00000029515 | O88989 | Mdh1 | 10116.ENSRNOP00000011429 | 624 |
| Q6P6V0 | Gpi | 10116.ENSRNOP00000029515 | P49432 | Pdhb | 10116.ENSRNOP00000010545 | 889 |
| Q6P6V0 | Gpi | 10116.ENSRNOP00000029515 | P00507 | Got2 | 10116.ENSRNOP00000015956 | 450 |
| Q6P6V0 | Gpi | 10116.ENSRNOP00000029515 | Q9EQS0 | Taldo1 | 10116.ENSRNOP00000024863 | 971 |
| Q6P6V0 | Gpi | 10116.ENSRNOP00000029515 | O35077 | Gpd1 | 10116.ENSRNOP00000026200 | 624 |
| Q6P6V0 | Gpi | 10116.ENSRNOP00000029515 | P05708 | Hk1 | 10116.ENSRNOP00000066611 | 969 |
| P52303 | Ap1b1 | 10116.ENSRNOP00000054218 | P18588 | Mx1 | 10116.ENSRNOP00000043001 | 577 |
| P00507 | Got2 | 10116.ENSRNOP00000015956 | P10960 | Psap | 10116.ENSRNOP00000000696 | 643 |
| P00507 | Got2 | 10116.ENSRNOP00000015956 | Q64611 | Csad | 10116.ENSRNOP00000016205 | 818 |
| P00507 | Got2 | 10116.ENSRNOP00000015956 | P49432 | Pdhb | 10116.ENSRNOP00000010545 | 482 |
| P00507 | Got2 | 10116.ENSRNOP00000015956 | P41562 | Idh1 | 10116.ENSRNOP00000020322 | 918 |
| P00507 | Got2 | 10116.ENSRNOP00000015956 | P30839 | Aldh3a2 | 10116.ENSRNOP00000061762 | 845 |
| P00507 | Got2 | 10116.ENSRNOP00000015956 | O88989 | Mdh1 | 10116.ENSRNOP00000011429 | 979 |
| P00507 | Got2 | 10116.ENSRNOP00000015956 | P41565 | Idh3g | 10116.ENSRNOP00000053220 | 858 |
| P00507 | Got2 | 10116.ENSRNOP00000015956 | P25409 | Gpt | 10116.ENSRNOP00000044411 | 726 |
| P00507 | Got2 | 10116.ENSRNOP00000015956 | Q9JLJ3 | Aldh9a1 | 10116.ENSRNOP00000005611 | 831 |
| P00507 | Got2 | 10116.ENSRNOP00000015956 | O35244 | Prdx6 | 10116.ENSRNOP00000030323 | 923 |
| P00507 | Got2 | 10116.ENSRNOP00000015956 | Q497B0 | Nit2 | 10116.ENSRNOP00000034144 | 902 |
| P00507 | Got2 | 10116.ENSRNOP00000015956 | P13697 | Me1 | 10116.ENSRNOP00000013244 | 472 |
| P54290 | Cacna2d1 | 10116.ENSRNOP00000034572 | P54283 | Cacnb1 | 10116.ENSRNOP00000006098 | 983 |
| P54290 | Cacna2d1 | 10116.ENSRNOP00000034572 | P41350 | Cav1 | 10116.ENSRNOP00000009253 | 655 |
| Q641Y0 | Ddost | 10116.ENSRNOP00000062365 | Q09325 | Mgat1 | 10116.ENSRNOP00000044560 | 414 |
| Q641Y0 | Ddost | 10116.ENSRNOP00000062365 | P60892 | Prps1 | 10116.ENSRNOP00000029405 | 401 |
| O35077 | Gpd1 | 10116.ENSRNOP00000026200 | P35571 | Gpd2 | 10116.ENSRNOP00000043749 | 909 |
| O35077 | Gpd1 | 10116.ENSRNOP00000026200 | P35738 | Bckdhb | 10116.ENSRNOP00000013249 | 402 |
| O35077 | Gpd1 | 10116.ENSRNOP00000026200 | P05708 | Hk1 | 10116.ENSRNOP00000066611 | 404 |
| O35077 | Gpd1 | 10116.ENSRNOP00000026200 | O88989 | Mdh1 | 10116.ENSRNOP00000011429 | 559 |
| Q68FU3 | Etfb | 10116.ENSRNOP00000024083 | O70351 | Hsd17b10 | 10116.ENSRNOP00000043608 | 917 |
| Q68FU3 | Etfb | 10116.ENSRNOP00000024083 | Q5XIC0 | Eci2 | 10116.ENSRNOP00000022022 | 580 |
| Q68FU3 | Etfb | 10116.ENSRNOP00000024083 | P13803 | Etfa | 10116.ENSRNOP00000020544 | 999 |
| Q68FU3 | Etfb | 10116.ENSRNOP00000024083 | P08503 | Acadm | 10116.ENSRNOP00000013238 | 854 |
| Q68FU3 | Etfb | 10116.ENSRNOP00000024083 | P31399 | Atp5h | 10116.ENSRNOP00000004836 | 484 |
| Q68FU3 | Etfb | 10116.ENSRNOP00000024083 | P23965 | Eci1 | 10116.ENSRNOP00000011784 | 744 |
| Q68FU3 | Etfb | 10116.ENSRNOP00000024083 | Q6AYG5 | Echdc1 | 10116.ENSRNOP00000015440 | 687 |
| O88989 | Mdh1 | 10116.ENSRNOP00000011429 | Q9JLJ3 | Aldh9a1 | 10116.ENSRNOP00000005611 | 566 |
| O88989 | Mdh1 | 10116.ENSRNOP00000011429 | P41565 | Idh3g | 10116.ENSRNOP00000053220 | 433 |
| O88989 | Mdh1 | 10116.ENSRNOP00000011429 | P25113 | Pgam1 | 10116.ENSRNOP00000065690 | 416 |
| O88989 | Mdh1 | 10116.ENSRNOP00000011429 | P28037 | Aldh1l1 | 10116.ENSRNOP00000065882 | 566 |
| O88989 | Mdh1 | 10116.ENSRNOP00000011429 | Q8K4D8 | Aldh1a3 | 10116.ENSRNOP00000045261 | 566 |
| O88989 | Mdh1 | 10116.ENSRNOP00000011429 | P08461 | Dlat | 10116.ENSRNOP00000032890 | 663 |
| O88989 | Mdh1 | 10116.ENSRNOP00000011429 | P05708 | Hk1 | 10116.ENSRNOP00000066611 | 479 |
| O88989 | Mdh1 | 10116.ENSRNOP00000011429 | P25409 | Gpt | 10116.ENSRNOP00000044411 | 607 |
| O88989 | Mdh1 | 10116.ENSRNOP00000011429 | P49432 | Pdhb | 10116.ENSRNOP00000010545 | 591 |
| O88989 | Mdh1 | 10116.ENSRNOP00000011429 | O70351 | Hsd17b10 | 10116.ENSRNOP00000043608 | 465 |
| O88989 | Mdh1 | 10116.ENSRNOP00000011429 | Q7TPB1 | Cct4 | 10116.ENSRNOP00000012847 | 549 |
| O88989 | Mdh1 | 10116.ENSRNOP00000011429 | P13697 | Me1 | 10116.ENSRNOP00000013244 | 976 |
| O88989 | Mdh1 | 10116.ENSRNOP00000011429 | P13803 | Etfa | 10116.ENSRNOP00000020544 | 621 |
| O88989 | Mdh1 | 10116.ENSRNOP00000011429 | O35244 | Prdx6 | 10116.ENSRNOP00000030323 | 443 |
| O88989 | Mdh1 | 10116.ENSRNOP00000011429 | Q68FQ0 | Cct5 | 10116.ENSRNOP00000015886 | 462 |
| O88989 | Mdh1 | 10116.ENSRNOP00000011429 | P35171 | Cox7a2 | 10116.ENSRNOP00000060398 | 510 |
| O88989 | Mdh1 | 10116.ENSRNOP00000011429 | Q9EQS0 | Taldo1 | 10116.ENSRNOP00000024863 | 607 |
| O88989 | Mdh1 | 10116.ENSRNOP00000011429 | P30839 | Aldh3a2 | 10116.ENSRNOP00000061762 | 581 |
| O88989 | Mdh1 | 10116.ENSRNOP00000011429 | P41562 | Idh1 | 10116.ENSRNOP00000020322 | 900 |
| O88989 | Mdh1 | 10116.ENSRNOP00000011429 | Q497B0 | Nit2 | 10116.ENSRNOP00000034144 | 809 |
| O88989 | Mdh1 | 10116.ENSRNOP00000011429 | Q6AYG5 | Echdc1 | 10116.ENSRNOP00000015440 | 475 |
| O88989 | Mdh1 | 10116.ENSRNOP00000011429 | P60892 | Prps1 | 10116.ENSRNOP00000029405 | 426 |
| P08010 | Gstm2 | 10116.ENSRNOP00000025939 | Q8K4D8 | Aldh1a3 | 10116.ENSRNOP00000045261 | 906 |
| P08010 | Gstm2 | 10116.ENSRNOP00000025939 | P04041 | Gpx1 | 10116.ENSRNOP00000066577 | 927 |
| P08010 | Gstm2 | 10116.ENSRNOP00000025939 | P08011 | Mgst1 | 10116.ENSRNOP00000010579 | 956 |
| P11762 | Lgals1 | 10116.ENSRNOP00000013538 | O70513 | Lgals3bp | 10116.ENSRNOP00000004320 | 479 |
| P55314 | C8b | 10116.ENSRNOP00000010100 | F1MA98 | Tpr | 10116.ENSRNOP00000062172 | 540 |
| Q64119 | Myl6 | 10116.ENSRNOP00000067282 | P17209 | Myl4 | 10116.ENSRNOP00000064224 | 542 |
| Q64119 | Myl6 | 10116.ENSRNOP00000067282 | Q8CFN2 | Cdc42 | 10116.ENSRNOP00000030928 | 624 |
| Q64119 | Myl6 | 10116.ENSRNOP00000067282 | P13832 | Rlc-a | 10116.ENSRNOP00000021048 | 482 |
| O35244 | Prdx6 | 10116.ENSRNOP00000030323 | Q497B0 | Nit2 | 10116.ENSRNOP00000034144 | 644 |
| O35244 | Prdx6 | 10116.ENSRNOP00000030323 | O88767 | Park7 | 10116.ENSRNOP00000024711 | 565 |
| O35244 | Prdx6 | 10116.ENSRNOP00000030323 | Q5I0H9 | Pdia5 | 10116.ENSRNOP00000059945 | 610 |
| O35244 | Prdx6 | 10116.ENSRNOP00000030323 | P26772 | Hspe1 | 10116.ENSRNOP00000020066 | 407 |
| O35244 | Prdx6 | 10116.ENSRNOP00000030323 | Q6AXM5 | Cept1 | 10116.ENSRNOP00000032597 | 652 |
| O35244 | Prdx6 | 10116.ENSRNOP00000030323 | P04041 | Gpx1 | 10116.ENSRNOP00000066577 | 641 |
| O35244 | Prdx6 | 10116.ENSRNOP00000030323 | Q9JLZ1 | Glrx3 | 10116.ENSRNOP00000022406 | 462 |
| Q08163 | Cap1 | 10116.ENSRNOP00000018711 | Q7M0E3 | Dstn | 10116.ENSRNOP00000007794 | 936 |
| Q08163 | Cap1 | 10116.ENSRNOP00000018711 | P63259 | Actg1 | 10116.ENSRNOP00000044296 | 701 |
| Q08163 | Cap1 | 10116.ENSRNOP00000018711 | P49134 | Itgb1 | 10116.ENSRNOP00000014785 | 692 |
| Q08163 | Cap1 | 10116.ENSRNOP00000018711 | Q63538 | Mapk12 | 10116.ENSRNOP00000046455 | 549 |
| Q08163 | Cap1 | 10116.ENSRNOP00000018711 | Q91Y81 | Sept2 | 10116.ENSRNOP00000024261 | 499 |
| Q08163 | Cap1 | 10116.ENSRNOP00000018711 | Q6AYZ1 | Tuba1c | 10116.ENSRNOP00000020932 | 463 |
| P11530 | Dmd | 10116.ENSRNOP00000029969 | Q8CFN2 | Cdc42 | 10116.ENSRNOP00000030928 | 697 |
| P11530 | Dmd | 10116.ENSRNOP00000029969 | P49134 | Itgb1 | 10116.ENSRNOP00000014785 | 735 |
| P04041 | Gpx1 | 10116.ENSRNOP00000066577 | Q9JLZ1 | Glrx3 | 10116.ENSRNOP00000022406 | 446 |
| P04041 | Gpx1 | 10116.ENSRNOP00000066577 | P06762 | Hmox1 | 10116.ENSRNOP00000019192 | 672 |
| P04041 | Gpx1 | 10116.ENSRNOP00000066577 | P08011 | Mgst1 | 10116.ENSRNOP00000010579 | 914 |
| P04041 | Gpx1 | 10116.ENSRNOP00000066577 | Q5I0H9 | Pdia5 | 10116.ENSRNOP00000059945 | 446 |
| Q64240 | Ambp | 10116.ENSRNOP00000009248 | P00787 | Ctsb | 10116.ENSRNOP00000014178 | 724 |
| Q64240 | Ambp | 10116.ENSRNOP00000009248 | P17220 | Psma2 | 10116.ENSRNOP00000066950 | 446 |
| P13803 | Etfa | 10116.ENSRNOP00000020544 | P08503 | Acadm | 10116.ENSRNOP00000013238 | 924 |
| P13803 | Etfa | 10116.ENSRNOP00000020544 | P49432 | Pdhb | 10116.ENSRNOP00000010545 | 493 |
| P13803 | Etfa | 10116.ENSRNOP00000020544 | P08461 | Dlat | 10116.ENSRNOP00000032890 | 459 |
| P13803 | Etfa | 10116.ENSRNOP00000020544 | P23965 | Eci1 | 10116.ENSRNOP00000011784 | 776 |
| P13803 | Etfa | 10116.ENSRNOP00000020544 | Q6AYG5 | Echdc1 | 10116.ENSRNOP00000015440 | 892 |
| P13803 | Etfa | 10116.ENSRNOP00000020544 | Q5XIC0 | Eci2 | 10116.ENSRNOP00000022022 | 767 |
| P13803 | Etfa | 10116.ENSRNOP00000020544 | P31399 | Atp5h | 10116.ENSRNOP00000004836 | 540 |
| P13803 | Etfa | 10116.ENSRNOP00000020544 | O70351 | Hsd17b10 | 10116.ENSRNOP00000043608 | 490 |
| P68255 | Ywhaq | 10116.ENSRNOP00000011501 | P50878 | Rpl4 | 10116.ENSRNOP00000013462 | 453 |
| P68255 | Ywhaq | 10116.ENSRNOP00000011501 | Q63538 | Mapk12 | 10116.ENSRNOP00000046455 | 545 |
| P68255 | Ywhaq | 10116.ENSRNOP00000011501 | P61983 | Ywhag | 10116.ENSRNOP00000001954 | 684 |
| B0BNN3 | Ca1 | 10116.ENSRNOP00000014267 | P08699 | Lgals3 | 10116.ENSRNOP00000014216 | 420 |
| P20761 | Igh-1a | 10116.ENSRNOP00000044037 | P31720 | C1qa | 10116.ENSRNOP00000017385 | 900 |
| P20761 | Igh-1a | 10116.ENSRNOP00000044037 | P63259 | Actg1 | 10116.ENSRNOP00000044296 | 900 |
| P20761 | Igh-1a | 10116.ENSRNOP00000044037 | P20759 | ENSRNOG00000030332 | 10116.ENSRNOP00000006975 | 900 |
| P20761 | Igh-1a | 10116.ENSRNOP00000044037 | Q6IE52 | Mug2 | 10116.ENSRNOP00000019969 | 465 |
| P85834 | Tufm | 10116.ENSRNOP00000025203 | P49432 | Pdhb | 10116.ENSRNOP00000010545 | 432 |
| P85834 | Tufm | 10116.ENSRNOP00000025203 | P62914 | Rpl11 | 10116.ENSRNOP00000037110 | 793 |
| P85834 | Tufm | 10116.ENSRNOP00000025203 | P62752 | Rpl23a | 10116.ENSRNOP00000036391 | 662 |
| P85834 | Tufm | 10116.ENSRNOP00000025203 | Q68FQ0 | Cct5 | 10116.ENSRNOP00000015886 | 654 |
| P85834 | Tufm | 10116.ENSRNOP00000025203 | P60868 | Rps20 | 10116.ENSRNOP00000066077 | 870 |
| P85834 | Tufm | 10116.ENSRNOP00000025203 | P23358 | Rpl12 | 10116.ENSRNOP00000041462 | 869 |
| P85834 | Tufm | 10116.ENSRNOP00000025203 | Q04462 | Vars | 10116.ENSRNOP00000001160 | 481 |
| P85834 | Tufm | 10116.ENSRNOP00000025203 | P62832 | Rpl23 | 10116.ENSRNOP00000005471 | 715 |
| P85834 | Tufm | 10116.ENSRNOP00000025203 | A0JPJ7 | Ola1 | 10116.ENSRNOP00000026040 | 553 |
| P85834 | Tufm | 10116.ENSRNOP00000025203 | P35427 | Rpl13a | 10116.ENSRNOP00000027976 | 429 |
| P85834 | Tufm | 10116.ENSRNOP00000025203 | Q6P799 | Sars | 10116.ENSRNOP00000038448 | 477 |
| P85834 | Tufm | 10116.ENSRNOP00000025203 | P50878 | Rpl4 | 10116.ENSRNOP00000013462 | 760 |
| P85834 | Tufm | 10116.ENSRNOP00000025203 | P26772 | Hspe1 | 10116.ENSRNOP00000020066 | 456 |
| P85834 | Tufm | 10116.ENSRNOP00000025203 | Q7TPB1 | Cct4 | 10116.ENSRNOP00000012847 | 655 |
| P85834 | Tufm | 10116.ENSRNOP00000025203 | P18445 | Rpl27a | 10116.ENSRNOP00000019247 | 720 |
| P85834 | Tufm | 10116.ENSRNOP00000025203 | P62278 | Rps13 | 10116.ENSRNOP00000036690 | 658 |
| Q6IFW6 | Krt10 | 10116.ENSRNOP00000029821 | Q6IG05 | Krt75 | 10116.ENSRNOP00000012640 | 579 |
| P49134 | Itgb1 | 10116.ENSRNOP00000014785 | Q8CFN2 | Cdc42 | 10116.ENSRNOP00000030928 | 714 |
| P49134 | Itgb1 | 10116.ENSRNOP00000014785 | Q91Y81 | Sept2 | 10116.ENSRNOP00000024261 | 407 |
| P49134 | Itgb1 | 10116.ENSRNOP00000014785 | P08699 | Lgals3 | 10116.ENSRNOP00000014216 | 717 |
| P49134 | Itgb1 | 10116.ENSRNOP00000014785 | Q63691 | Cd14 | 10116.ENSRNOP00000023977 | 619 |
| P49134 | Itgb1 | 10116.ENSRNOP00000014785 | Q63258 | Itga7 | 10116.ENSRNOP00000058370 | 995 |
| P49134 | Itgb1 | 10116.ENSRNOP00000014785 | P41350 | Cav1 | 10116.ENSRNOP00000009253 | 939 |
| P49134 | Itgb1 | 10116.ENSRNOP00000014785 | P13383 | Ncl | 10116.ENSRNOP00000024712 | 535 |
| P49134 | Itgb1 | 10116.ENSRNOP00000014785 | Q7M0E3 | Dstn | 10116.ENSRNOP00000007794 | 799 |
| P49134 | Itgb1 | 10116.ENSRNOP00000014785 | P63259 | Actg1 | 10116.ENSRNOP00000044296 | 922 |
| P06686 | Atp1a2 | 10116.ENSRNOP00000054947 | P11505 | Atp2b1 | 10116.ENSRNOP00000005491 | 822 |
| P06686 | Atp1a2 | 10116.ENSRNOP00000054947 | P54283 | Cacnb1 | 10116.ENSRNOP00000006098 | 418 |
| P13832 | Rlc-a | 10116.ENSRNOP00000021048 | P17209 | Myl4 | 10116.ENSRNOP00000064224 | 490 |
| P13832 | Rlc-a | 10116.ENSRNOP00000021048 | Q6AYZ1 | Tuba1c | 10116.ENSRNOP00000020932 | 409 |
| P13832 | Rlc-a | 10116.ENSRNOP00000021048 | Q8CFN2 | Cdc42 | 10116.ENSRNOP00000030928 | 544 |
| P13832 | Rlc-a | 10116.ENSRNOP00000021048 | P63259 | Actg1 | 10116.ENSRNOP00000044296 | 866 |
| P49432 | Pdhb | 10116.ENSRNOP00000010545 | P08503 | Acadm | 10116.ENSRNOP00000013238 | 609 |
| P49432 | Pdhb | 10116.ENSRNOP00000010545 | P08461 | Dlat | 10116.ENSRNOP00000032890 | 999 |
| P49432 | Pdhb | 10116.ENSRNOP00000010545 | P41562 | Idh1 | 10116.ENSRNOP00000020322 | 515 |
| P49432 | Pdhb | 10116.ENSRNOP00000010545 | P41565 | Idh3g | 10116.ENSRNOP00000053220 | 878 |
| P49432 | Pdhb | 10116.ENSRNOP00000010545 | Q63525 | Nudc | 10116.ENSRNOP00000009933 | 483 |
| P49432 | Pdhb | 10116.ENSRNOP00000010545 | P28037 | Aldh1l1 | 10116.ENSRNOP00000065882 | 479 |
| P49432 | Pdhb | 10116.ENSRNOP00000010545 | P35571 | Gpd2 | 10116.ENSRNOP00000043749 | 460 |
| P49432 | Pdhb | 10116.ENSRNOP00000010545 | Q8K4D8 | Aldh1a3 | 10116.ENSRNOP00000045261 | 479 |
| P49432 | Pdhb | 10116.ENSRNOP00000010545 | P30839 | Aldh3a2 | 10116.ENSRNOP00000061762 | 479 |
| P49432 | Pdhb | 10116.ENSRNOP00000010545 | Q9JLJ3 | Aldh9a1 | 10116.ENSRNOP00000005611 | 535 |
| P49432 | Pdhb | 10116.ENSRNOP00000010545 | P35738 | Bckdhb | 10116.ENSRNOP00000013249 | 623 |
| P49432 | Pdhb | 10116.ENSRNOP00000010545 | Q9EQS0 | Taldo1 | 10116.ENSRNOP00000024863 | 903 |
| P49432 | Pdhb | 10116.ENSRNOP00000010545 | P31399 | Atp5h | 10116.ENSRNOP00000004836 | 441 |
| P49432 | Pdhb | 10116.ENSRNOP00000010545 | P25113 | Pgam1 | 10116.ENSRNOP00000065690 | 685 |
| P49432 | Pdhb | 10116.ENSRNOP00000010545 | P13697 | Me1 | 10116.ENSRNOP00000013244 | 956 |
| O08651 | Phgdh | 10116.ENSRNOP00000053019 | P23457 | Akr1c9 | 10116.ENSRNOP00000023835 | 421 |
| O08651 | Phgdh | 10116.ENSRNOP00000053019 | P25113 | Pgam1 | 10116.ENSRNOP00000065690 | 913 |
| O08651 | Phgdh | 10116.ENSRNOP00000053019 | P41565 | Idh3g | 10116.ENSRNOP00000053220 | 489 |
| O08651 | Phgdh | 10116.ENSRNOP00000053019 | Q7M0E3 | Dstn | 10116.ENSRNOP00000007794 | 582 |
| O08651 | Phgdh | 10116.ENSRNOP00000053019 | P07943 | Akr1b1 | 10116.ENSRNOP00000012879 | 530 |
| P07943 | Akr1b1 | 10116.ENSRNOP00000012879 | P30839 | Aldh3a2 | 10116.ENSRNOP00000061762 | 579 |
| P07943 | Akr1b1 | 10116.ENSRNOP00000012879 | Q9JLJ3 | Aldh9a1 | 10116.ENSRNOP00000005611 | 587 |
| P07943 | Akr1b1 | 10116.ENSRNOP00000012879 | Q64611 | Csad | 10116.ENSRNOP00000016205 | 558 |
| P07943 | Akr1b1 | 10116.ENSRNOP00000012879 | Q8K4D8 | Aldh1a3 | 10116.ENSRNOP00000045261 | 529 |
| P07943 | Akr1b1 | 10116.ENSRNOP00000012879 | P28037 | Aldh1l1 | 10116.ENSRNOP00000065882 | 521 |
| O08629 | Trim28 | 10116.ENSRNOP00000031061 | P63281 | Ube2i | 10116.ENSRNOP00000024406 | 481 |
| O08629 | Trim28 | 10116.ENSRNOP00000031061 | Q68FQ0 | Cct5 | 10116.ENSRNOP00000015886 | 713 |
| P05708 | Hk1 | 10116.ENSRNOP00000066611 | P25113 | Pgam1 | 10116.ENSRNOP00000065690 | 435 |
| P05708 | Hk1 | 10116.ENSRNOP00000066611 | Q9EQS0 | Taldo1 | 10116.ENSRNOP00000024863 | 857 |
| P05708 | Hk1 | 10116.ENSRNOP00000066611 | Q66HG4 | Galm | 10116.ENSRNOP00000009221 | 937 |
| P50878 | Rpl4 | 10116.ENSRNOP00000013462 | P60868 | Rps20 | 10116.ENSRNOP00000066077 | 995 |
| P50878 | Rpl4 | 10116.ENSRNOP00000013462 | P41123 | Rpl13 | 10116.ENSRNOP00000020635 | 998 |
| P50878 | Rpl4 | 10116.ENSRNOP00000013462 | P21533 | Rpl6 | 10116.ENSRNOP00000051135 | 999 |
| P50878 | Rpl4 | 10116.ENSRNOP00000013462 | Q4KMA2 | Rad23b | 10116.ENSRNOP00000021629 | 402 |
| P50878 | Rpl4 | 10116.ENSRNOP00000013462 | P05765 | Rps21 | 10116.ENSRNOP00000008458 | 971 |
| P50878 | Rpl4 | 10116.ENSRNOP00000013462 | P17220 | Psma2 | 10116.ENSRNOP00000066950 | 439 |
| P50878 | Rpl4 | 10116.ENSRNOP00000013462 | Q68FQ0 | Cct5 | 10116.ENSRNOP00000015886 | 682 |
| P50878 | Rpl4 | 10116.ENSRNOP00000013462 | P62278 | Rps13 | 10116.ENSRNOP00000036690 | 990 |
| P50878 | Rpl4 | 10116.ENSRNOP00000013462 | P62832 | Rpl23 | 10116.ENSRNOP00000005471 | 994 |
| P50878 | Rpl4 | 10116.ENSRNOP00000013462 | P23358 | Rpl12 | 10116.ENSRNOP00000041462 | 980 |
| P50878 | Rpl4 | 10116.ENSRNOP00000013462 | Q04462 | Vars | 10116.ENSRNOP00000001160 | 479 |
| P50878 | Rpl4 | 10116.ENSRNOP00000013462 | Q63413 | Ddx39b | 10116.ENSRNOP00000001115 | 455 |
| P50878 | Rpl4 | 10116.ENSRNOP00000013462 | P62718 | Rpl18a | 10116.ENSRNOP00000025421 | 998 |
| P50878 | Rpl4 | 10116.ENSRNOP00000013462 | P62634 | Cnbp | 10116.ENSRNOP00000013884 | 536 |
| P50878 | Rpl4 | 10116.ENSRNOP00000013462 | Q7TPB1 | Cct4 | 10116.ENSRNOP00000012847 | 571 |
| P50878 | Rpl4 | 10116.ENSRNOP00000013462 | P35427 | Rpl13a | 10116.ENSRNOP00000027976 | 999 |
| P50878 | Rpl4 | 10116.ENSRNOP00000013462 | P62752 | Rpl23a | 10116.ENSRNOP00000036391 | 997 |
| P50878 | Rpl4 | 10116.ENSRNOP00000013462 | P62914 | Rpl11 | 10116.ENSRNOP00000037110 | 999 |
| P50878 | Rpl4 | 10116.ENSRNOP00000013462 | P18445 | Rpl27a | 10116.ENSRNOP00000019247 | 997 |
| P23358 | Rpl12 | 10116.ENSRNOP00000041462 | P60868 | Rps20 | 10116.ENSRNOP00000066077 | 979 |
| P23358 | Rpl12 | 10116.ENSRNOP00000041462 | P62752 | Rpl23a | 10116.ENSRNOP00000036391 | 959 |
| P23358 | Rpl12 | 10116.ENSRNOP00000041462 | P18445 | Rpl27a | 10116.ENSRNOP00000019247 | 968 |
| P23358 | Rpl12 | 10116.ENSRNOP00000041462 | Q68FQ0 | Cct5 | 10116.ENSRNOP00000015886 | 442 |
| P23358 | Rpl12 | 10116.ENSRNOP00000041462 | P62832 | Rpl23 | 10116.ENSRNOP00000005471 | 961 |
| P23358 | Rpl12 | 10116.ENSRNOP00000041462 | P05765 | Rps21 | 10116.ENSRNOP00000008458 | 942 |
| P23358 | Rpl12 | 10116.ENSRNOP00000041462 | P62718 | Rpl18a | 10116.ENSRNOP00000025421 | 995 |
| P23358 | Rpl12 | 10116.ENSRNOP00000041462 | P21533 | Rpl6 | 10116.ENSRNOP00000051135 | 944 |
| P23358 | Rpl12 | 10116.ENSRNOP00000041462 | P35427 | Rpl13a | 10116.ENSRNOP00000027976 | 991 |
| P23358 | Rpl12 | 10116.ENSRNOP00000041462 | P62278 | Rps13 | 10116.ENSRNOP00000036690 | 983 |
| P23358 | Rpl12 | 10116.ENSRNOP00000041462 | P41123 | Rpl13 | 10116.ENSRNOP00000020635 | 980 |
| P23358 | Rpl12 | 10116.ENSRNOP00000041462 | P62914 | Rpl11 | 10116.ENSRNOP00000037110 | 991 |
| Q63413 | Ddx39b | 10116.ENSRNOP00000001115 | P62278 | Rps13 | 10116.ENSRNOP00000036690 | 427 |
| Q63413 | Ddx39b | 10116.ENSRNOP00000001115 | Q27W01 | Rbm8a | 10116.ENSRNOP00000028807 | 555 |
| Q63413 | Ddx39b | 10116.ENSRNOP00000001115 | Q7TPB1 | Cct4 | 10116.ENSRNOP00000012847 | 629 |
| Q63413 | Ddx39b | 10116.ENSRNOP00000001115 | Q68FQ0 | Cct5 | 10116.ENSRNOP00000015886 | 516 |
| G3V9R8 | Hnrnpc | 10116.ENSRNOP00000057257 | Q27W01 | Rbm8a | 10116.ENSRNOP00000028807 | 927 |
| G3V9R8 | Hnrnpc | 10116.ENSRNOP00000057257 | F1LNJ2 | Snrnp200 | 10116.ENSRNOP00000048598 | 933 |
| P00787 | Ctsb | 10116.ENSRNOP00000014178 | P14841 | Cst3 | 10116.ENSRNOP00000007175 | 698 |
| P00787 | Ctsb | 10116.ENSRNOP00000014178 | P10960 | Psap | 10116.ENSRNOP00000000696 | 579 |
| P13697 | Me1 | 10116.ENSRNOP00000013244 | Q7TPB1 | Cct4 | 10116.ENSRNOP00000012847 | 665 |
| P13697 | Me1 | 10116.ENSRNOP00000013244 | P25409 | Gpt | 10116.ENSRNOP00000044411 | 532 |
| P13697 | Me1 | 10116.ENSRNOP00000013244 | P35738 | Bckdhb | 10116.ENSRNOP00000013249 | 499 |
| P13697 | Me1 | 10116.ENSRNOP00000013244 | P08461 | Dlat | 10116.ENSRNOP00000032890 | 730 |
| P13697 | Me1 | 10116.ENSRNOP00000013244 | P41562 | Idh1 | 10116.ENSRNOP00000020322 | 448 |
| Q9JLJ3 | Aldh9a1 | 10116.ENSRNOP00000005611 | P35738 | Bckdhb | 10116.ENSRNOP00000013249 | 522 |
| Q9JLJ3 | Aldh9a1 | 10116.ENSRNOP00000005611 | Q6AYG5 | Echdc1 | 10116.ENSRNOP00000015440 | 480 |
| Q9JLJ3 | Aldh9a1 | 10116.ENSRNOP00000005611 | P08503 | Acadm | 10116.ENSRNOP00000013238 | 598 |
| Q9JLJ3 | Aldh9a1 | 10116.ENSRNOP00000005611 | P35745 | Acyp2 | 10116.ENSRNOP00000061028 | 900 |
| Q9JLJ3 | Aldh9a1 | 10116.ENSRNOP00000005611 | P25113 | Pgam1 | 10116.ENSRNOP00000065690 | 489 |
| Q9JLJ3 | Aldh9a1 | 10116.ENSRNOP00000005611 | P23965 | Eci1 | 10116.ENSRNOP00000011784 | 495 |
| Q9JLJ3 | Aldh9a1 | 10116.ENSRNOP00000005611 | Q499N5 | Acsf2 | 10116.ENSRNOP00000004673 | 458 |
| Q9JLJ3 | Aldh9a1 | 10116.ENSRNOP00000005611 | Q5XIC0 | Eci2 | 10116.ENSRNOP00000022022 | 578 |
| Q9JLJ3 | Aldh9a1 | 10116.ENSRNOP00000005611 | P23457 | Akr1c9 | 10116.ENSRNOP00000023835 | 541 |
| Q9JLJ3 | Aldh9a1 | 10116.ENSRNOP00000005611 | Q64611 | Csad | 10116.ENSRNOP00000016205 | 529 |
| Q9JLJ3 | Aldh9a1 | 10116.ENSRNOP00000005611 | Q7TP48 | Apmap | 10116.ENSRNOP00000043524 | 467 |
| Q9JLJ3 | Aldh9a1 | 10116.ENSRNOP00000005611 | Q64591 | Decr1 | 10116.ENSRNOP00000011330 | 551 |
| Q9JLJ3 | Aldh9a1 | 10116.ENSRNOP00000005611 | Q497B0 | Nit2 | 10116.ENSRNOP00000034144 | 431 |
| Q9JLJ3 | Aldh9a1 | 10116.ENSRNOP00000005611 | Q02253 | Aldh6a1 | 10116.ENSRNOP00000015545 | 919 |
| F1MA98 | Tpr | 10116.ENSRNOP00000062172 | Q6AYH5 | Dctn2 | 10116.ENSRNOP00000008120 | 720 |
| F1MA98 | Tpr | 10116.ENSRNOP00000062172 | Q27W01 | Rbm8a | 10116.ENSRNOP00000028807 | 900 |
| Q7TPB1 | Cct4 | 10116.ENSRNOP00000012847 | P62193 | Psmc1 | 10116.ENSRNOP00000005329 | 483 |
| Q7TPB1 | Cct4 | 10116.ENSRNOP00000012847 | P60892 | Prps1 | 10116.ENSRNOP00000029405 | 486 |
| Q7TPB1 | Cct4 | 10116.ENSRNOP00000012847 | O88767 | Park7 | 10116.ENSRNOP00000024711 | 470 |
| Q7TPB1 | Cct4 | 10116.ENSRNOP00000012847 | P62634 | Cnbp | 10116.ENSRNOP00000013884 | 506 |
| Q7TPB1 | Cct4 | 10116.ENSRNOP00000012847 | Q6AYK6 | Cacybp | 10116.ENSRNOP00000003586 | 436 |
| Q7TPB1 | Cct4 | 10116.ENSRNOP00000012847 | P62914 | Rpl11 | 10116.ENSRNOP00000037110 | 497 |
| Q7TPB1 | Cct4 | 10116.ENSRNOP00000012847 | P35427 | Rpl13a | 10116.ENSRNOP00000027976 | 425 |
| Q7TPB1 | Cct4 | 10116.ENSRNOP00000012847 | P21533 | Rpl6 | 10116.ENSRNOP00000051135 | 481 |
| Q7TPB1 | Cct4 | 10116.ENSRNOP00000012847 | Q63525 | Nudc | 10116.ENSRNOP00000009933 | 449 |
| Q7TPB1 | Cct4 | 10116.ENSRNOP00000012847 | P63259 | Actg1 | 10116.ENSRNOP00000044296 | 966 |
| Q7TPB1 | Cct4 | 10116.ENSRNOP00000012847 | Q9QYU1 | Pex19 | 10116.ENSRNOP00000065867 | 703 |
| Q7TPB1 | Cct4 | 10116.ENSRNOP00000012847 | Q04462 | Vars | 10116.ENSRNOP00000001160 | 546 |
| Q7TPB1 | Cct4 | 10116.ENSRNOP00000012847 | Q6AYZ1 | Tuba1c | 10116.ENSRNOP00000020932 | 917 |
| Q7TPB1 | Cct4 | 10116.ENSRNOP00000012847 | P26772 | Hspe1 | 10116.ENSRNOP00000020066 | 998 |
| Q7TPB1 | Cct4 | 10116.ENSRNOP00000012847 | Q68FQ0 | Cct5 | 10116.ENSRNOP00000015886 | 999 |
| Q7TPB1 | Cct4 | 10116.ENSRNOP00000012847 | Q4KMA2 | Rad23b | 10116.ENSRNOP00000021629 | 416 |
| Q7TPB1 | Cct4 | 10116.ENSRNOP00000012847 | Q6P799 | Sars | 10116.ENSRNOP00000038448 | 429 |
| Q7TPB1 | Cct4 | 10116.ENSRNOP00000012847 | A0JPJ7 | Ola1 | 10116.ENSRNOP00000026040 | 581 |
| Q7TPB1 | Cct4 | 10116.ENSRNOP00000012847 | P50475 | Aars | 10116.ENSRNOP00000025051 | 432 |
| P31044 | Pebp1 | 10116.ENSRNOP00000001500 | P26772 | Hspe1 | 10116.ENSRNOP00000020066 | 503 |
| P31044 | Pebp1 | 10116.ENSRNOP00000001500 | P31399 | Atp5h | 10116.ENSRNOP00000004836 | 503 |
| P31044 | Pebp1 | 10116.ENSRNOP00000001500 | P35427 | Rpl13a | 10116.ENSRNOP00000027976 | 584 |
| P41123 | Rpl13 | 10116.ENSRNOP00000020635 | P62914 | Rpl11 | 10116.ENSRNOP00000037110 | 998 |
| P41123 | Rpl13 | 10116.ENSRNOP00000020635 | P62718 | Rpl18a | 10116.ENSRNOP00000025421 | 993 |
| P41123 | Rpl13 | 10116.ENSRNOP00000020635 | P21533 | Rpl6 | 10116.ENSRNOP00000051135 | 994 |
| P41123 | Rpl13 | 10116.ENSRNOP00000020635 | Q6IFU8 | Krt17 | 10116.ENSRNOP00000005382 | 552 |
| P41123 | Rpl13 | 10116.ENSRNOP00000020635 | P62752 | Rpl23a | 10116.ENSRNOP00000036391 | 966 |
| P41123 | Rpl13 | 10116.ENSRNOP00000020635 | Q04462 | Vars | 10116.ENSRNOP00000001160 | 609 |
| P41123 | Rpl13 | 10116.ENSRNOP00000020635 | P05765 | Rps21 | 10116.ENSRNOP00000008458 | 988 |
| P41123 | Rpl13 | 10116.ENSRNOP00000020635 | P18445 | Rpl27a | 10116.ENSRNOP00000019247 | 997 |
| P41123 | Rpl13 | 10116.ENSRNOP00000020635 | P62832 | Rpl23 | 10116.ENSRNOP00000005471 | 986 |
| P41123 | Rpl13 | 10116.ENSRNOP00000020635 | P35427 | Rpl13a | 10116.ENSRNOP00000027976 | 992 |
| P41123 | Rpl13 | 10116.ENSRNOP00000020635 | P62278 | Rps13 | 10116.ENSRNOP00000036690 | 984 |
| P41123 | Rpl13 | 10116.ENSRNOP00000020635 | P60868 | Rps20 | 10116.ENSRNOP00000066077 | 965 |
| P25409 | Gpt | 10116.ENSRNOP00000044411 | P08461 | Dlat | 10116.ENSRNOP00000032890 | 407 |
| Q63514 | C4bpa | 10116.ENSRNOP00000005461 | Q6IE52 | Mug2 | 10116.ENSRNOP00000019969 | 610 |
| P25113 | Pgam1 | 10116.ENSRNOP00000065690 | Q8K4D8 | Aldh1a3 | 10116.ENSRNOP00000045261 | 489 |
| P25113 | Pgam1 | 10116.ENSRNOP00000065690 | P08503 | Acadm | 10116.ENSRNOP00000013238 | 461 |
| P25113 | Pgam1 | 10116.ENSRNOP00000065690 | P28037 | Aldh1l1 | 10116.ENSRNOP00000065882 | 474 |
| P25113 | Pgam1 | 10116.ENSRNOP00000065690 | Q66HG4 | Galm | 10116.ENSRNOP00000009221 | 411 |
| P25113 | Pgam1 | 10116.ENSRNOP00000065690 | P26772 | Hspe1 | 10116.ENSRNOP00000020066 | 418 |
| P25113 | Pgam1 | 10116.ENSRNOP00000065690 | P30839 | Aldh3a2 | 10116.ENSRNOP00000061762 | 474 |
| P25113 | Pgam1 | 10116.ENSRNOP00000065690 | Q9EQS0 | Taldo1 | 10116.ENSRNOP00000024863 | 851 |
| Q9JLZ1 | Glrx3 | 10116.ENSRNOP00000022406 | G3V7W1 | Pdcd6 | 10116.ENSRNOP00000019735 | 479 |
| Q9JLZ1 | Glrx3 | 10116.ENSRNOP00000022406 | P35427 | Rpl13a | 10116.ENSRNOP00000027976 | 778 |
| Q9JLZ1 | Glrx3 | 10116.ENSRNOP00000022406 | O70351 | Hsd17b10 | 10116.ENSRNOP00000043608 | 814 |
| Q68FQ0 | Cct5 | 10116.ENSRNOP00000015886 | P60892 | Prps1 | 10116.ENSRNOP00000029405 | 616 |
| Q68FQ0 | Cct5 | 10116.ENSRNOP00000015886 | Q6AYK6 | Cacybp | 10116.ENSRNOP00000003586 | 611 |
| Q68FQ0 | Cct5 | 10116.ENSRNOP00000015886 | Q6DGG0 | Ppid | 10116.ENSRNOP00000035797 | 451 |
| Q68FQ0 | Cct5 | 10116.ENSRNOP00000015886 | Q6AYZ1 | Tuba1c | 10116.ENSRNOP00000020932 | 916 |
| Q68FQ0 | Cct5 | 10116.ENSRNOP00000015886 | P62193 | Psmc1 | 10116.ENSRNOP00000005329 | 477 |
| Q68FQ0 | Cct5 | 10116.ENSRNOP00000015886 | P62278 | Rps13 | 10116.ENSRNOP00000036690 | 438 |
| Q68FQ0 | Cct5 | 10116.ENSRNOP00000015886 | P35738 | Bckdhb | 10116.ENSRNOP00000013249 | 471 |
| Q68FQ0 | Cct5 | 10116.ENSRNOP00000015886 | A0JPJ7 | Ola1 | 10116.ENSRNOP00000026040 | 411 |
| Q68FQ0 | Cct5 | 10116.ENSRNOP00000015886 | P18445 | Rpl27a | 10116.ENSRNOP00000019247 | 401 |
| Q68FQ0 | Cct5 | 10116.ENSRNOP00000015886 | P62914 | Rpl11 | 10116.ENSRNOP00000037110 | 505 |
| Q68FQ0 | Cct5 | 10116.ENSRNOP00000015886 | P26772 | Hspe1 | 10116.ENSRNOP00000020066 | 998 |
| Q68FQ0 | Cct5 | 10116.ENSRNOP00000015886 | P63259 | Actg1 | 10116.ENSRNOP00000044296 | 931 |
| Q68FQ0 | Cct5 | 10116.ENSRNOP00000015886 | Q04462 | Vars | 10116.ENSRNOP00000001160 | 603 |
| Q68FQ0 | Cct5 | 10116.ENSRNOP00000015886 | P21533 | Rpl6 | 10116.ENSRNOP00000051135 | 405 |
| Q68FQ0 | Cct5 | 10116.ENSRNOP00000015886 | Q6P799 | Sars | 10116.ENSRNOP00000038448 | 428 |
| Q68FQ0 | Cct5 | 10116.ENSRNOP00000015886 | Q5I0G4 | Gars | 10116.ENSRNOP00000014780 | 482 |
| P10960 | Psap | 10116.ENSRNOP00000000696 | Q63083 | Nucb1 | 10116.ENSRNOP00000028390 | 829 |
| Q62920 | Pdlim5 | 10116.ENSRNOP00000022387 | P23965 | Eci1 | 10116.ENSRNOP00000011784 | 416 |
| Q04462 | Vars | 10116.ENSRNOP00000001160 | Q6P799 | Sars | 10116.ENSRNOP00000038448 | 612 |
| Q04462 | Vars | 10116.ENSRNOP00000001160 | P18445 | Rpl27a | 10116.ENSRNOP00000019247 | 455 |
| Q04462 | Vars | 10116.ENSRNOP00000001160 | Q5I0G4 | Gars | 10116.ENSRNOP00000014780 | 791 |
| Q04462 | Vars | 10116.ENSRNOP00000001160 | P62914 | Rpl11 | 10116.ENSRNOP00000037110 | 468 |
| Q04462 | Vars | 10116.ENSRNOP00000001160 | P50475 | Aars | 10116.ENSRNOP00000025051 | 751 |
| Q04462 | Vars | 10116.ENSRNOP00000001160 | P62278 | Rps13 | 10116.ENSRNOP00000036690 | 490 |
| Q04462 | Vars | 10116.ENSRNOP00000001160 | P60892 | Prps1 | 10116.ENSRNOP00000029405 | 423 |
| Q04462 | Vars | 10116.ENSRNOP00000001160 | A0JPJ7 | Ola1 | 10116.ENSRNOP00000026040 | 686 |
| Q9Z269 | Vapb | 10116.ENSRNOP00000007554 | P29418 | Atp5e | 10116.ENSRNOP00000067815 | 481 |
| Q9Z269 | Vapb | 10116.ENSRNOP00000007554 | Q6AXM5 | Cept1 | 10116.ENSRNOP00000032597 | 400 |
| P26772 | Hspe1 | 10116.ENSRNOP00000020066 | P35427 | Rpl13a | 10116.ENSRNOP00000027976 | 465 |
| P26772 | Hspe1 | 10116.ENSRNOP00000020066 | P05765 | Rps21 | 10116.ENSRNOP00000008458 | 442 |
| P26772 | Hspe1 | 10116.ENSRNOP00000020066 | P62959 | Hint1 | 10116.ENSRNOP00000000772 | 410 |
| P26772 | Hspe1 | 10116.ENSRNOP00000020066 | P62966 | Crabp1 | 10116.ENSRNOP00000033840 | 595 |
| P26772 | Hspe1 | 10116.ENSRNOP00000020066 | P17220 | Psma2 | 10116.ENSRNOP00000066950 | 475 |
| P26772 | Hspe1 | 10116.ENSRNOP00000020066 | P24155 | Thop1 | 10116.ENSRNOP00000027045 | 436 |
| P26772 | Hspe1 | 10116.ENSRNOP00000020066 | P21571 | Atp5j | 10116.ENSRNOP00000002116 | 712 |
| P26772 | Hspe1 | 10116.ENSRNOP00000020066 | P31399 | Atp5h | 10116.ENSRNOP00000004836 | 827 |
| P26772 | Hspe1 | 10116.ENSRNOP00000020066 | Q6DGG0 | Ppid | 10116.ENSRNOP00000035797 | 411 |
| P26772 | Hspe1 | 10116.ENSRNOP00000020066 | Q6AYK6 | Cacybp | 10116.ENSRNOP00000003586 | 497 |
| P26772 | Hspe1 | 10116.ENSRNOP00000020066 | P35171 | Cox7a2 | 10116.ENSRNOP00000060398 | 547 |
| P26772 | Hspe1 | 10116.ENSRNOP00000020066 | Q27W01 | Rbm8a | 10116.ENSRNOP00000028807 | 502 |
| Q62969 | Ptgis | 10116.ENSRNOP00000010891 | Q6IUR5 | Nenf | 10116.ENSRNOP00000005190 | 486 |
| Q62969 | Ptgis | 10116.ENSRNOP00000010891 | Q64611 | Csad | 10116.ENSRNOP00000016205 | 410 |
| Q62969 | Ptgis | 10116.ENSRNOP00000010891 | Q499N5 | Acsf2 | 10116.ENSRNOP00000004673 | 460 |
| Q7M0E3 | Dstn | 10116.ENSRNOP00000007794 | Q6AYK6 | Cacybp | 10116.ENSRNOP00000003586 | 483 |
| Q7M0E3 | Dstn | 10116.ENSRNOP00000007794 | P63259 | Actg1 | 10116.ENSRNOP00000044296 | 817 |
| Q7M0E3 | Dstn | 10116.ENSRNOP00000007794 | Q8CFN2 | Cdc42 | 10116.ENSRNOP00000030928 | 506 |
| P41350 | Cav1 | 10116.ENSRNOP00000009253 | Q62658 | Fkbp1a | 10116.ENSRNOP00000012608 | 575 |
| P41350 | Cav1 | 10116.ENSRNOP00000009253 | P15337 | Creb1 | 10116.ENSRNOP00000018326 | 773 |
| P41350 | Cav1 | 10116.ENSRNOP00000009253 | P06762 | Hmox1 | 10116.ENSRNOP00000019192 | 716 |
| P41350 | Cav1 | 10116.ENSRNOP00000009253 | Q8CFN2 | Cdc42 | 10116.ENSRNOP00000030928 | 816 |
| P41350 | Cav1 | 10116.ENSRNOP00000009253 | P63259 | Actg1 | 10116.ENSRNOP00000044296 | 611 |
| P41350 | Cav1 | 10116.ENSRNOP00000009253 | P01015 | Agt | 10116.ENSRNOP00000024917 | 765 |
| P41350 | Cav1 | 10116.ENSRNOP00000009253 | P15304 | Lipe | 10116.ENSRNOP00000027911 | 900 |
| P41350 | Cav1 | 10116.ENSRNOP00000009253 | Q63538 | Mapk12 | 10116.ENSRNOP00000046455 | 566 |
| P08503 | Acadm | 10116.ENSRNOP00000013238 | O70351 | Hsd17b10 | 10116.ENSRNOP00000043608 | 515 |
| P08503 | Acadm | 10116.ENSRNOP00000013238 | Q6AYG5 | Echdc1 | 10116.ENSRNOP00000015440 | 978 |
| P08503 | Acadm | 10116.ENSRNOP00000013238 | P07483 | Fabp3 | 10116.ENSRNOP00000017325 | 516 |
| P08503 | Acadm | 10116.ENSRNOP00000013238 | Q02253 | Aldh6a1 | 10116.ENSRNOP00000015545 | 926 |
| P08503 | Acadm | 10116.ENSRNOP00000013238 | P23965 | Eci1 | 10116.ENSRNOP00000011784 | 890 |
| P08503 | Acadm | 10116.ENSRNOP00000013238 | P08461 | Dlat | 10116.ENSRNOP00000032890 | 601 |
| P08503 | Acadm | 10116.ENSRNOP00000013238 | O55171 | Acot2 | 10116.ENSRNOP00000013515 | 426 |
| P08503 | Acadm | 10116.ENSRNOP00000013238 | Q499N5 | Acsf2 | 10116.ENSRNOP00000004673 | 702 |
| P08503 | Acadm | 10116.ENSRNOP00000013238 | Q5XIC0 | Eci2 | 10116.ENSRNOP00000022022 | 898 |
| P08503 | Acadm | 10116.ENSRNOP00000013238 | Q8K4D8 | Aldh1a3 | 10116.ENSRNOP00000045261 | 508 |
| P08503 | Acadm | 10116.ENSRNOP00000013238 | P30839 | Aldh3a2 | 10116.ENSRNOP00000061762 | 624 |
| P08503 | Acadm | 10116.ENSRNOP00000013238 | P28037 | Aldh1l1 | 10116.ENSRNOP00000065882 | 514 |
| P08503 | Acadm | 10116.ENSRNOP00000013238 | Q64591 | Decr1 | 10116.ENSRNOP00000011330 | 962 |
| P08503 | Acadm | 10116.ENSRNOP00000013238 | P35738 | Bckdhb | 10116.ENSRNOP00000013249 | 667 |
| P13383 | Ncl | 10116.ENSRNOP00000024712 | Q6B345 | S100a11 | 10116.ENSRNOP00000013393 | 755 |
| P13383 | Ncl | 10116.ENSRNOP00000024712 | P62914 | Rpl11 | 10116.ENSRNOP00000037110 | 665 |
| Q63691 | Cd14 | 10116.ENSRNOP00000023977 | Q6IFU8 | Krt17 | 10116.ENSRNOP00000005382 | 551 |
| O70351 | Hsd17b10 | 10116.ENSRNOP00000043608 | Q5XIC0 | Eci2 | 10116.ENSRNOP00000022022 | 642 |
| O70351 | Hsd17b10 | 10116.ENSRNOP00000043608 | P23965 | Eci1 | 10116.ENSRNOP00000011784 | 786 |
| O70351 | Hsd17b10 | 10116.ENSRNOP00000043608 | P80254 | Ddt | 10116.ENSRNOP00000001664 | 483 |
| O70351 | Hsd17b10 | 10116.ENSRNOP00000043608 | Q499N5 | Acsf2 | 10116.ENSRNOP00000004673 | 403 |
| O70351 | Hsd17b10 | 10116.ENSRNOP00000043608 | O88767 | Park7 | 10116.ENSRNOP00000024711 | 423 |
| O70351 | Hsd17b10 | 10116.ENSRNOP00000043608 | Q6AYG5 | Echdc1 | 10116.ENSRNOP00000015440 | 833 |
| O70351 | Hsd17b10 | 10116.ENSRNOP00000043608 | P35427 | Rpl13a | 10116.ENSRNOP00000027976 | 620 |
| P17220 | Psma2 | 10116.ENSRNOP00000066950 | P62193 | Psmc1 | 10116.ENSRNOP00000005329 | 991 |
| P17220 | Psma2 | 10116.ENSRNOP00000066950 | P21571 | Atp5j | 10116.ENSRNOP00000002116 | 538 |
| P17220 | Psma2 | 10116.ENSRNOP00000066950 | P62959 | Hint1 | 10116.ENSRNOP00000000772 | 434 |
| P17220 | Psma2 | 10116.ENSRNOP00000066950 | Q496Z0 | Elp2 | 10116.ENSRNOP00000020601 | 720 |
| P17220 | Psma2 | 10116.ENSRNOP00000066950 | P62914 | Rpl11 | 10116.ENSRNOP00000037110 | 452 |
| P17220 | Psma2 | 10116.ENSRNOP00000066950 | Q4KMA2 | Rad23b | 10116.ENSRNOP00000021629 | 713 |
| P28037 | Aldh1l1 | 10116.ENSRNOP00000065882 | Q5XIC0 | Eci2 | 10116.ENSRNOP00000022022 | 481 |
| P28037 | Aldh1l1 | 10116.ENSRNOP00000065882 | Q64591 | Decr1 | 10116.ENSRNOP00000011330 | 531 |
| P28037 | Aldh1l1 | 10116.ENSRNOP00000065882 | Q6AYG5 | Echdc1 | 10116.ENSRNOP00000015440 | 480 |
| P28037 | Aldh1l1 | 10116.ENSRNOP00000065882 | Q499N5 | Acsf2 | 10116.ENSRNOP00000004673 | 458 |
| P28037 | Aldh1l1 | 10116.ENSRNOP00000065882 | Q64611 | Csad | 10116.ENSRNOP00000016205 | 529 |
| P28037 | Aldh1l1 | 10116.ENSRNOP00000065882 | P35738 | Bckdhb | 10116.ENSRNOP00000013249 | 479 |
| P28037 | Aldh1l1 | 10116.ENSRNOP00000065882 | P23457 | Akr1c9 | 10116.ENSRNOP00000023835 | 523 |
| P28037 | Aldh1l1 | 10116.ENSRNOP00000065882 | P23965 | Eci1 | 10116.ENSRNOP00000011784 | 480 |
| Q64611 | Csad | 10116.ENSRNOP00000016205 | Q8K4D8 | Aldh1a3 | 10116.ENSRNOP00000045261 | 529 |
| Q64611 | Csad | 10116.ENSRNOP00000016205 | P08461 | Dlat | 10116.ENSRNOP00000032890 | 401 |
| Q64611 | Csad | 10116.ENSRNOP00000016205 | P30839 | Aldh3a2 | 10116.ENSRNOP00000061762 | 529 |
| Q9EQS0 | Taldo1 | 10116.ENSRNOP00000024863 | P35738 | Bckdhb | 10116.ENSRNOP00000013249 | 568 |
| Q9EQS0 | Taldo1 | 10116.ENSRNOP00000024863 | P08461 | Dlat | 10116.ENSRNOP00000032890 | 458 |
| P35571 | Gpd2 | 10116.ENSRNOP00000043749 | P08461 | Dlat | 10116.ENSRNOP00000032890 | 523 |
| P35571 | Gpd2 | 10116.ENSRNOP00000043749 | P35738 | Bckdhb | 10116.ENSRNOP00000013249 | 447 |
| Q6AYH5 | Dctn2 | 10116.ENSRNOP00000008120 | P63259 | Actg1 | 10116.ENSRNOP00000044296 | 594 |
| Q6AYH5 | Dctn2 | 10116.ENSRNOP00000008120 | P61983 | Ywhag | 10116.ENSRNOP00000001954 | 902 |
| Q8CFN2 | Cdc42 | 10116.ENSRNOP00000030928 | B0BNF1 | Sept8 | 10116.ENSRNOP00000063893 | 455 |
| Q8CFN2 | Cdc42 | 10116.ENSRNOP00000030928 | P55161 | Nckap1 | 10116.ENSRNOP00000059233 | 454 |
| Q8CFN2 | Cdc42 | 10116.ENSRNOP00000030928 | Q63538 | Mapk12 | 10116.ENSRNOP00000046455 | 975 |
| Q8CFN2 | Cdc42 | 10116.ENSRNOP00000030928 | Q91Y81 | Sept2 | 10116.ENSRNOP00000024261 | 772 |
| Q8CFN2 | Cdc42 | 10116.ENSRNOP00000030928 | Q64591 | Decr1 | 10116.ENSRNOP00000011330 | 539 |
| Q8CFN2 | Cdc42 | 10116.ENSRNOP00000030928 | P01015 | Agt | 10116.ENSRNOP00000024917 | 555 |
| Q8CFN2 | Cdc42 | 10116.ENSRNOP00000030928 | P50398 | Gdi1 | 10116.ENSRNOP00000053135 | 943 |
| Q8CFN2 | Cdc42 | 10116.ENSRNOP00000030928 | P63259 | Actg1 | 10116.ENSRNOP00000044296 | 912 |
| Q8CFN2 | Cdc42 | 10116.ENSRNOP00000030928 | P15337 | Creb1 | 10116.ENSRNOP00000018326 | 660 |
| P27139 | Ca2 | 10116.ENSRNOP00000013354 | P15337 | Creb1 | 10116.ENSRNOP00000018326 | 457 |
| P27139 | Ca2 | 10116.ENSRNOP00000013354 | P08699 | Lgals3 | 10116.ENSRNOP00000014216 | 687 |
| Q6IFU8 | Krt17 | 10116.ENSRNOP00000005382 | Q6IG05 | Krt75 | 10116.ENSRNOP00000012640 | 543 |
| Q6IFU8 | Krt17 | 10116.ENSRNOP00000005382 | Q6P6Q2 | Krt5 | 10116.ENSRNOP00000011644 | 903 |
| Q6IFU8 | Krt17 | 10116.ENSRNOP00000005382 | Q6IFV3 | Krt15 | 10116.ENSRNOP00000019037 | 904 |
| P21961 | Cpa3 | 10116.ENSRNOP00000014970 | P01015 | Agt | 10116.ENSRNOP00000024917 | 902 |
| Q63525 | Nudc | 10116.ENSRNOP00000009933 | Q6AYK6 | Cacybp | 10116.ENSRNOP00000003586 | 657 |
| P35427 | Rpl13a | 10116.ENSRNOP00000027976 | P62278 | Rps13 | 10116.ENSRNOP00000036690 | 983 |
| P35427 | Rpl13a | 10116.ENSRNOP00000027976 | P62914 | Rpl11 | 10116.ENSRNOP00000037110 | 997 |
| P35427 | Rpl13a | 10116.ENSRNOP00000027976 | P60868 | Rps20 | 10116.ENSRNOP00000066077 | 980 |
| P35427 | Rpl13a | 10116.ENSRNOP00000027976 | P62832 | Rpl23 | 10116.ENSRNOP00000005471 | 981 |
| P35427 | Rpl13a | 10116.ENSRNOP00000027976 | P63259 | Actg1 | 10116.ENSRNOP00000044296 | 621 |
| P35427 | Rpl13a | 10116.ENSRNOP00000027976 | P62718 | Rpl18a | 10116.ENSRNOP00000025421 | 998 |
| P35427 | Rpl13a | 10116.ENSRNOP00000027976 | P21533 | Rpl6 | 10116.ENSRNOP00000051135 | 983 |
| P35427 | Rpl13a | 10116.ENSRNOP00000027976 | P62752 | Rpl23a | 10116.ENSRNOP00000036391 | 984 |
| P35427 | Rpl13a | 10116.ENSRNOP00000027976 | P18445 | Rpl27a | 10116.ENSRNOP00000019247 | 998 |
| P35427 | Rpl13a | 10116.ENSRNOP00000027976 | P05765 | Rps21 | 10116.ENSRNOP00000008458 | 979 |
| O88767 | Park7 | 10116.ENSRNOP00000024711 | P21571 | Atp5j | 10116.ENSRNOP00000002116 | 482 |
| O88767 | Park7 | 10116.ENSRNOP00000024711 | P62914 | Rpl11 | 10116.ENSRNOP00000037110 | 420 |
| O88767 | Park7 | 10116.ENSRNOP00000024711 | P84817 | Fis1 | 10116.ENSRNOP00000061895 | 665 |
| O88767 | Park7 | 10116.ENSRNOP00000024711 | P31399 | Atp5h | 10116.ENSRNOP00000004836 | 505 |
| Q64591 | Decr1 | 10116.ENSRNOP00000011330 | Q8K4D8 | Aldh1a3 | 10116.ENSRNOP00000045261 | 531 |
| Q64591 | Decr1 | 10116.ENSRNOP00000011330 | P06762 | Hmox1 | 10116.ENSRNOP00000019192 | 841 |
| Q64591 | Decr1 | 10116.ENSRNOP00000011330 | Q6AYG5 | Echdc1 | 10116.ENSRNOP00000015440 | 502 |
| Q64591 | Decr1 | 10116.ENSRNOP00000011330 | Q5XIC0 | Eci2 | 10116.ENSRNOP00000022022 | 718 |
| Q64591 | Decr1 | 10116.ENSRNOP00000011330 | P30839 | Aldh3a2 | 10116.ENSRNOP00000061762 | 565 |
| Q64591 | Decr1 | 10116.ENSRNOP00000011330 | P01015 | Agt | 10116.ENSRNOP00000024917 | 846 |
| Q64591 | Decr1 | 10116.ENSRNOP00000011330 | P23965 | Eci1 | 10116.ENSRNOP00000011784 | 977 |
| Q64591 | Decr1 | 10116.ENSRNOP00000011330 | Q499N5 | Acsf2 | 10116.ENSRNOP00000004673 | 522 |
| Q64591 | Decr1 | 10116.ENSRNOP00000011330 | Q63538 | Mapk12 | 10116.ENSRNOP00000046455 | 577 |
| Q64591 | Decr1 | 10116.ENSRNOP00000011330 | D3ZBP4 | Mical1 | 10116.ENSRNOP00000000337 | 508 |
| P05765 | Rps21 | 10116.ENSRNOP00000008458 | P62832 | Rpl23 | 10116.ENSRNOP00000005471 | 987 |
| P05765 | Rps21 | 10116.ENSRNOP00000008458 | P18445 | Rpl27a | 10116.ENSRNOP00000019247 | 975 |
| P05765 | Rps21 | 10116.ENSRNOP00000008458 | P62914 | Rpl11 | 10116.ENSRNOP00000037110 | 987 |
| P05765 | Rps21 | 10116.ENSRNOP00000008458 | Q4KMA2 | Rad23b | 10116.ENSRNOP00000021629 | 400 |
| P05765 | Rps21 | 10116.ENSRNOP00000008458 | P62278 | Rps13 | 10116.ENSRNOP00000036690 | 990 |
| P05765 | Rps21 | 10116.ENSRNOP00000008458 | P35171 | Cox7a2 | 10116.ENSRNOP00000060398 | 403 |
| P05765 | Rps21 | 10116.ENSRNOP00000008458 | P62752 | Rpl23a | 10116.ENSRNOP00000036391 | 988 |
| P05765 | Rps21 | 10116.ENSRNOP00000008458 | P60868 | Rps20 | 10116.ENSRNOP00000066077 | 994 |
| P05765 | Rps21 | 10116.ENSRNOP00000008458 | P29418 | Atp5e | 10116.ENSRNOP00000067815 | 722 |
| P05765 | Rps21 | 10116.ENSRNOP00000008458 | P21533 | Rpl6 | 10116.ENSRNOP00000051135 | 971 |
| P05765 | Rps21 | 10116.ENSRNOP00000008458 | P62718 | Rpl18a | 10116.ENSRNOP00000025421 | 997 |
| Q63258 | Itga7 | 10116.ENSRNOP00000058370 | P63259 | Actg1 | 10116.ENSRNOP00000044296 | 803 |
| Q6P799 | Sars | 10116.ENSRNOP00000038448 | P62278 | Rps13 | 10116.ENSRNOP00000036690 | 510 |
| Q6P799 | Sars | 10116.ENSRNOP00000038448 | Q5I0G4 | Gars | 10116.ENSRNOP00000014780 | 876 |
| Q6P799 | Sars | 10116.ENSRNOP00000038448 | P50475 | Aars | 10116.ENSRNOP00000025051 | 705 |
| Q9R1T3 | Ctsz | 10116.ENSRNOP00000066523 | P29418 | Atp5e | 10116.ENSRNOP00000067815 | 562 |
| Q9R1T3 | Ctsz | 10116.ENSRNOP00000066523 | P01015 | Agt | 10116.ENSRNOP00000024917 | 916 |
| B0BNF1 | Sept8 | 10116.ENSRNOP00000063893 | Q91Y81 | Sept2 | 10116.ENSRNOP00000024261 | 746 |
| P30839 | Aldh3a2 | 10116.ENSRNOP00000061762 | P23457 | Akr1c9 | 10116.ENSRNOP00000023835 | 543 |
| P30839 | Aldh3a2 | 10116.ENSRNOP00000061762 | Q6AYG5 | Echdc1 | 10116.ENSRNOP00000015440 | 480 |
| P30839 | Aldh3a2 | 10116.ENSRNOP00000061762 | P23965 | Eci1 | 10116.ENSRNOP00000011784 | 504 |
| P30839 | Aldh3a2 | 10116.ENSRNOP00000061762 | P35745 | Acyp2 | 10116.ENSRNOP00000061028 | 900 |
| P30839 | Aldh3a2 | 10116.ENSRNOP00000061762 | Q499N5 | Acsf2 | 10116.ENSRNOP00000004673 | 458 |
| P30839 | Aldh3a2 | 10116.ENSRNOP00000061762 | Q5XIC0 | Eci2 | 10116.ENSRNOP00000022022 | 584 |
| P30839 | Aldh3a2 | 10116.ENSRNOP00000061762 | P35738 | Bckdhb | 10116.ENSRNOP00000013249 | 479 |
| P30839 | Aldh3a2 | 10116.ENSRNOP00000061762 | Q02253 | Aldh6a1 | 10116.ENSRNOP00000015545 | 920 |
| P08699 | Lgals3 | 10116.ENSRNOP00000014216 | O70513 | Lgals3bp | 10116.ENSRNOP00000004320 | 981 |
| P08699 | Lgals3 | 10116.ENSRNOP00000014216 | P14841 | Cst3 | 10116.ENSRNOP00000007175 | 436 |
| P18588 | Mx1 | 10116.ENSRNOP00000043001 | P84817 | Fis1 | 10116.ENSRNOP00000061895 | 759 |
| P18588 | Mx1 | 10116.ENSRNOP00000043001 | P63259 | Actg1 | 10116.ENSRNOP00000044296 | 635 |
| P18588 | Mx1 | 10116.ENSRNOP00000043001 | Q9QYU1 | Pex19 | 10116.ENSRNOP00000065867 | 659 |
| P18588 | Mx1 | 10116.ENSRNOP00000043001 | P97840 | Lgals9 | 10116.ENSRNOP00000017042 | 418 |
| P61983 | Ywhag | 10116.ENSRNOP00000001954 | Q63538 | Mapk12 | 10116.ENSRNOP00000046455 | 544 |
| P61983 | Ywhag | 10116.ENSRNOP00000001954 | P63259 | Actg1 | 10116.ENSRNOP00000044296 | 433 |
| P50475 | Aars | 10116.ENSRNOP00000025051 | Q5I0G4 | Gars | 10116.ENSRNOP00000014780 | 986 |
| P50475 | Aars | 10116.ENSRNOP00000025051 | P62959 | Hint1 | 10116.ENSRNOP00000000772 | 419 |
| P01015 | Agt | 10116.ENSRNOP00000024917 | Q9JID2 | Gna11 | 10116.ENSRNOP00000007498 | 950 |
| P01015 | Agt | 10116.ENSRNOP00000024917 | Q63538 | Mapk12 | 10116.ENSRNOP00000046455 | 802 |
| P01015 | Agt | 10116.ENSRNOP00000024917 | P06762 | Hmox1 | 10116.ENSRNOP00000019192 | 890 |
| P01015 | Agt | 10116.ENSRNOP00000024917 | P15337 | Creb1 | 10116.ENSRNOP00000018326 | 925 |
| P08461 | Dlat | 10116.ENSRNOP00000032890 | P41562 | Idh1 | 10116.ENSRNOP00000020322 | 613 |
| P08461 | Dlat | 10116.ENSRNOP00000032890 | P41565 | Idh3g | 10116.ENSRNOP00000053220 | 891 |
| P08461 | Dlat | 10116.ENSRNOP00000032890 | P35738 | Bckdhb | 10116.ENSRNOP00000013249 | 965 |
| Q5XFX0 | Tagln2 | 10116.ENSRNOP00000011208 | P63259 | Actg1 | 10116.ENSRNOP00000044296 | 485 |
| P31399 | Atp5h | 10116.ENSRNOP00000004836 | P35171 | Cox7a2 | 10116.ENSRNOP00000060398 | 905 |
| P31399 | Atp5h | 10116.ENSRNOP00000004836 | P62914 | Rpl11 | 10116.ENSRNOP00000037110 | 485 |
| P31399 | Atp5h | 10116.ENSRNOP00000004836 | P21571 | Atp5j | 10116.ENSRNOP00000002116 | 998 |
| P31399 | Atp5h | 10116.ENSRNOP00000004836 | P29418 | Atp5e | 10116.ENSRNOP00000067815 | 998 |
| P15304 | Lipe | 10116.ENSRNOP00000027911 | Q499N5 | Acsf2 | 10116.ENSRNOP00000004673 | 485 |
| Q497B0 | Nit2 | 10116.ENSRNOP00000034144 | P41562 | Idh1 | 10116.ENSRNOP00000020322 | 806 |
| Q497B0 | Nit2 | 10116.ENSRNOP00000034144 | P41565 | Idh3g | 10116.ENSRNOP00000053220 | 800 |
| P41562 | Idh1 | 10116.ENSRNOP00000020322 | P41565 | Idh3g | 10116.ENSRNOP00000053220 | 974 |
| P41562 | Idh1 | 10116.ENSRNOP00000020322 | P32089 | Slc25a1 | 10116.ENSRNOP00000000306 | 528 |
| P41562 | Idh1 | 10116.ENSRNOP00000020322 | P35738 | Bckdhb | 10116.ENSRNOP00000013249 | 445 |
| Q02253 | Aldh6a1 | 10116.ENSRNOP00000015545 | P35738 | Bckdhb | 10116.ENSRNOP00000013249 | 439 |
| Q02253 | Aldh6a1 | 10116.ENSRNOP00000015545 | Q6AYG5 | Echdc1 | 10116.ENSRNOP00000015440 | 926 |
| P47967 | Lgals5 | 10116.ENSRNOP00000016897 | O70513 | Lgals3bp | 10116.ENSRNOP00000004320 | 507 |
| P12007 | Ivd | 10116.ENSRNOP00000013829 | Q6AYG5 | Echdc1 | 10116.ENSRNOP00000015440 | 517 |
| P12007 | Ivd | 10116.ENSRNOP00000013829 | P23965 | Eci1 | 10116.ENSRNOP00000011784 | 533 |
| P12007 | Ivd | 10116.ENSRNOP00000013829 | Q5XIC0 | Eci2 | 10116.ENSRNOP00000022022 | 495 |
| P12007 | Ivd | 10116.ENSRNOP00000013829 | P35738 | Bckdhb | 10116.ENSRNOP00000013249 | 950 |
| P62914 | Rpl11 | 10116.ENSRNOP00000037110 | P62832 | Rpl23 | 10116.ENSRNOP00000005471 | 998 |
| P62914 | Rpl11 | 10116.ENSRNOP00000037110 | P60868 | Rps20 | 10116.ENSRNOP00000066077 | 993 |
| P62914 | Rpl11 | 10116.ENSRNOP00000037110 | P29418 | Atp5e | 10116.ENSRNOP00000067815 | 418 |
| P62914 | Rpl11 | 10116.ENSRNOP00000037110 | P62278 | Rps13 | 10116.ENSRNOP00000036690 | 978 |
| P62914 | Rpl11 | 10116.ENSRNOP00000037110 | Q4KMA2 | Rad23b | 10116.ENSRNOP00000021629 | 449 |
| P62914 | Rpl11 | 10116.ENSRNOP00000037110 | P62718 | Rpl18a | 10116.ENSRNOP00000025421 | 998 |
| P62914 | Rpl11 | 10116.ENSRNOP00000037110 | P18445 | Rpl27a | 10116.ENSRNOP00000019247 | 999 |
| P62914 | Rpl11 | 10116.ENSRNOP00000037110 | P21533 | Rpl6 | 10116.ENSRNOP00000051135 | 997 |
| P62914 | Rpl11 | 10116.ENSRNOP00000037110 | P62752 | Rpl23a | 10116.ENSRNOP00000036391 | 996 |
| P62278 | Rps13 | 10116.ENSRNOP00000036690 | P62832 | Rpl23 | 10116.ENSRNOP00000005471 | 987 |
| P62278 | Rps13 | 10116.ENSRNOP00000036690 | P62752 | Rpl23a | 10116.ENSRNOP00000036391 | 987 |
| P62278 | Rps13 | 10116.ENSRNOP00000036690 | P62718 | Rpl18a | 10116.ENSRNOP00000025421 | 990 |
| P62278 | Rps13 | 10116.ENSRNOP00000036690 | P21533 | Rpl6 | 10116.ENSRNOP00000051135 | 990 |
| P62278 | Rps13 | 10116.ENSRNOP00000036690 | P18445 | Rpl27a | 10116.ENSRNOP00000019247 | 986 |
| P62278 | Rps13 | 10116.ENSRNOP00000036690 | P60868 | Rps20 | 10116.ENSRNOP00000066077 | 995 |
| O70513 | Lgals3bp | 10116.ENSRNOP00000004320 | P14841 | Cst3 | 10116.ENSRNOP00000007175 | 436 |
| O70513 | Lgals3bp | 10116.ENSRNOP00000004320 | P97840 | Lgals9 | 10116.ENSRNOP00000017042 | 777 |
| Q6DGG0 | Ppid | 10116.ENSRNOP00000035797 | P55161 | Nckap1 | 10116.ENSRNOP00000059233 | 420 |
| Q8K4D8 | Aldh1a3 | 10116.ENSRNOP00000045261 | Q5XIC0 | Eci2 | 10116.ENSRNOP00000022022 | 488 |
| Q8K4D8 | Aldh1a3 | 10116.ENSRNOP00000045261 | Q6AYG5 | Echdc1 | 10116.ENSRNOP00000015440 | 480 |
| Q8K4D8 | Aldh1a3 | 10116.ENSRNOP00000045261 | P23457 | Akr1c9 | 10116.ENSRNOP00000023835 | 481 |
| Q8K4D8 | Aldh1a3 | 10116.ENSRNOP00000045261 | Q499N5 | Acsf2 | 10116.ENSRNOP00000004673 | 458 |
| Q8K4D8 | Aldh1a3 | 10116.ENSRNOP00000045261 | P35738 | Bckdhb | 10116.ENSRNOP00000013249 | 479 |
| Q8K4D8 | Aldh1a3 | 10116.ENSRNOP00000045261 | P08011 | Mgst1 | 10116.ENSRNOP00000010579 | 900 |
| Q8K4D8 | Aldh1a3 | 10116.ENSRNOP00000045261 | P23965 | Eci1 | 10116.ENSRNOP00000011784 | 480 |
| P62193 | Psmc1 | 10116.ENSRNOP00000005329 | Q4KMA2 | Rad23b | 10116.ENSRNOP00000021629 | 887 |
| P62193 | Psmc1 | 10116.ENSRNOP00000005329 | Q496Z0 | Elp2 | 10116.ENSRNOP00000020601 | 727 |
| P60868 | Rps20 | 10116.ENSRNOP00000066077 | P21533 | Rpl6 | 10116.ENSRNOP00000051135 | 964 |
| P60868 | Rps20 | 10116.ENSRNOP00000066077 | P62752 | Rpl23a | 10116.ENSRNOP00000036391 | 992 |
| P60868 | Rps20 | 10116.ENSRNOP00000066077 | P62832 | Rpl23 | 10116.ENSRNOP00000005471 | 990 |
| P60868 | Rps20 | 10116.ENSRNOP00000066077 | P18445 | Rpl27a | 10116.ENSRNOP00000019247 | 992 |
| P60868 | Rps20 | 10116.ENSRNOP00000066077 | P62718 | Rpl18a | 10116.ENSRNOP00000025421 | 989 |
| Q6AYZ1 | Tuba1c | 10116.ENSRNOP00000020932 | P63259 | Actg1 | 10116.ENSRNOP00000044296 | 958 |
| Q5I0G4 | Gars | 10116.ENSRNOP00000014780 | Q5RJR8 | Lrrc59 | 10116.ENSRNOP00000004941 | 574 |
| P18445 | Rpl27a | 10116.ENSRNOP00000019247 | A0JPJ7 | Ola1 | 10116.ENSRNOP00000026040 | 415 |
| P18445 | Rpl27a | 10116.ENSRNOP00000019247 | P62752 | Rpl23a | 10116.ENSRNOP00000036391 | 985 |
| P18445 | Rpl27a | 10116.ENSRNOP00000019247 | P62832 | Rpl23 | 10116.ENSRNOP00000005471 | 992 |
| P18445 | Rpl27a | 10116.ENSRNOP00000019247 | P62718 | Rpl18a | 10116.ENSRNOP00000025421 | 998 |
| P18445 | Rpl27a | 10116.ENSRNOP00000019247 | P21533 | Rpl6 | 10116.ENSRNOP00000051135 | 993 |
| P18445 | Rpl27a | 10116.ENSRNOP00000019247 | P29418 | Atp5e | 10116.ENSRNOP00000067815 | 626 |
| P08011 | Mgst1 | 10116.ENSRNOP00000010579 | P20291 | Alox5ap | 10116.ENSRNOP00000001207 | 596 |
| P62966 | Crabp1 | 10116.ENSRNOP00000033840 | P15337 | Creb1 | 10116.ENSRNOP00000018326 | 550 |
| P62752 | Rpl23a | 10116.ENSRNOP00000036391 | P62832 | Rpl23 | 10116.ENSRNOP00000005471 | 992 |
| P62752 | Rpl23a | 10116.ENSRNOP00000036391 | P62718 | Rpl18a | 10116.ENSRNOP00000025421 | 977 |
| P62752 | Rpl23a | 10116.ENSRNOP00000036391 | Q4KMA2 | Rad23b | 10116.ENSRNOP00000021629 | 429 |
| P62752 | Rpl23a | 10116.ENSRNOP00000036391 | P21533 | Rpl6 | 10116.ENSRNOP00000051135 | 953 |
| P62752 | Rpl23a | 10116.ENSRNOP00000036391 | P29418 | Atp5e | 10116.ENSRNOP00000067815 | 488 |
| P21571 | Atp5j | 10116.ENSRNOP00000002116 | P35171 | Cox7a2 | 10116.ENSRNOP00000060398 | 894 |
| P21571 | Atp5j | 10116.ENSRNOP00000002116 | P29418 | Atp5e | 10116.ENSRNOP00000067815 | 991 |
| Q27W01 | Rbm8a | 10116.ENSRNOP00000028807 | F1LNJ2 | Snrnp200 | 10116.ENSRNOP00000048598 | 908 |
| P20759 | ENSRNOG00000030332 | 10116.ENSRNOP00000006975 | P31720 | C1qa | 10116.ENSRNOP00000017385 | 900 |
| P20759 | ENSRNOG00000030332 | 10116.ENSRNOP00000006975 | P63259 | Actg1 | 10116.ENSRNOP00000044296 | 909 |
| Q6AYG5 | Echdc1 | 10116.ENSRNOP00000015440 | Q5XIC0 | Eci2 | 10116.ENSRNOP00000022022 | 508 |
| Q6AYG5 | Echdc1 | 10116.ENSRNOP00000015440 | P23965 | Eci1 | 10116.ENSRNOP00000011784 | 645 |
| Q6AYG5 | Echdc1 | 10116.ENSRNOP00000015440 | Q499N5 | Acsf2 | 10116.ENSRNOP00000004673 | 533 |
| P23965 | Eci1 | 10116.ENSRNOP00000011784 | Q5XIC0 | Eci2 | 10116.ENSRNOP00000022022 | 811 |
| P23965 | Eci1 | 10116.ENSRNOP00000011784 | Q499N5 | Acsf2 | 10116.ENSRNOP00000004673 | 533 |
| P63259 | Actg1 | 10116.ENSRNOP00000044296 | Q63538 | Mapk12 | 10116.ENSRNOP00000046455 | 458 |
| P35738 | Bckdhb | 10116.ENSRNOP00000013249 | P41565 | Idh3g | 10116.ENSRNOP00000053220 | 658 |
| Q5XIC0 | Eci2 | 10116.ENSRNOP00000022022 | Q499N5 | Acsf2 | 10116.ENSRNOP00000004673 | 619 |
| P06762 | Hmox1 | 10116.ENSRNOP00000019192 | Q63538 | Mapk12 | 10116.ENSRNOP00000046455 | 773 |
| P06762 | Hmox1 | 10116.ENSRNOP00000019192 | P15337 | Creb1 | 10116.ENSRNOP00000018326 | 763 |
| P21533 | Rpl6 | 10116.ENSRNOP00000051135 | P62718 | Rpl18a | 10116.ENSRNOP00000025421 | 998 |
| P21533 | Rpl6 | 10116.ENSRNOP00000051135 | P62832 | Rpl23 | 10116.ENSRNOP00000005471 | 967 |
| P60892 | Prps1 | 10116.ENSRNOP00000029405 | A0JPJ7 | Ola1 | 10116.ENSRNOP00000026040 | 642 |
| Q4KMA2 | Rad23b | 10116.ENSRNOP00000021629 | Q496Z0 | Elp2 | 10116.ENSRNOP00000020601 | 619 |
| Q4KMA2 | Rad23b | 10116.ENSRNOP00000021629 | P62718 | Rpl18a | 10116.ENSRNOP00000025421 | 454 |
| Q4KMA2 | Rad23b | 10116.ENSRNOP00000021629 | P70711 | Ube2d2b | 10116.ENSRNOP00000039621 | 407 |
| P19527 | Nefl | 10116.ENSRNOP00000018599 | Q63538 | Mapk12 | 10116.ENSRNOP00000046455 | 826 |
| Q9ET61 | Cd93 | 10116.ENSRNOP00000034001 | P31720 | C1qa | 10116.ENSRNOP00000017385 | 798 |
| P62959 | Hint1 | 10116.ENSRNOP00000000772 | P35171 | Cox7a2 | 10116.ENSRNOP00000060398 | 557 |
| Q6IFV3 | Krt15 | 10116.ENSRNOP00000019037 | Q6IG05 | Krt75 | 10116.ENSRNOP00000012640 | 432 |
| Q6IFV3 | Krt15 | 10116.ENSRNOP00000019037 | Q6P6Q2 | Krt5 | 10116.ENSRNOP00000011644 | 915 |
| Q63538 | Mapk12 | 10116.ENSRNOP00000046455 | P15337 | Creb1 | 10116.ENSRNOP00000018326 | 889 |
| Q63538 | Mapk12 | 10116.ENSRNOP00000046455 | P31720 | C1qa | 10116.ENSRNOP00000017385 | 533 |
| Q63538 | Mapk12 | 10116.ENSRNOP00000046455 | Q9JID2 | Gna11 | 10116.ENSRNOP00000007498 | 590 |
| Q62658 | Fkbp1a | 10116.ENSRNOP00000012608 | P15337 | Creb1 | 10116.ENSRNOP00000018326 | 409 |
| P62718 | Rpl18a | 10116.ENSRNOP00000025421 | G3V7W1 | Pdcd6 | 10116.ENSRNOP00000019735 | 480 |
| P62718 | Rpl18a | 10116.ENSRNOP00000025421 | P62832 | Rpl23 | 10116.ENSRNOP00000005471 | 991 |
| P62718 | Rpl18a | 10116.ENSRNOP00000025421 | P29418 | Atp5e | 10116.ENSRNOP00000067815 | 618 |
| P35171 | Cox7a2 | 10116.ENSRNOP00000060398 | P29418 | Atp5e | 10116.ENSRNOP00000067815 | 837 |
