# Supplementary material for: Differential Proteomics Analysis of the Subcutaneous Connective Tissues in Alcian Blue Tracks along Conception Vessel and Adjacent Nonmeridian in Rats
Source: Evid Based Complement Alternat Med. 2021 May 4;2021:5550694. doi: 10.1155/2021/5550694 (PMC8116161; doi:10.1155/2021/5550694)
Supplement: Supplementary Materials — 1. The data of qualitative identification of protein. 2. The false discovery rate (FDR) of SWATH data. 3. The data of biological process (Gene Ontology (GO) analysis of differential proteins). 4. The data of cell component (GO analysis of differential proteins). 5. The data of molecular function (GO analysis of differential proteins). 6. The data of KEGG analysis. 7. The data of biological progress and KEEG link. 8. The data of protein-protein interaction (PPI). 9. The information analysis note. 10. The data of western blot analysis: the upregulated differential proteins involved in ATP metabolism (ATP5E, GAPDH), redox reactions (Gpx-3), and Ca2+ transmembrane transport (CACNA2D1). [file 5550694.f1.zip › 5550694.f1/9-information analysis note.docx]

**1. Data Analysis Workflow**

The query proteins are given as a list , these protein identifers were linked to the following databases, the [Quick GO](http://www.ebi.ac.uk/QuickGO) (Gene Ontology Analysis), the [KEGG Pathway](http://www.kegg.jp/kegg/pathway.html) (Pathway Analysis) and [STRING](http://string-db.org/) (Protein-Protein Interaction Analysis) for downstream analysis.


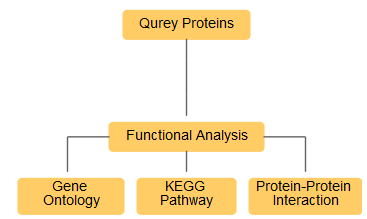


The statistics of the analysis is shown in table below. BP, CC, MF, KEGG are 4 categories of functional analysis, stands for Biological Process, Cellular Component, Molecule Function KEGG Pathway, respectively. Counts for each category represent the total associated terms in the database with the query gene/protein list. Terms with P-value < 0.05 are statistically significant. Empty list means the protein has not been annotated yet in the databases.

| Total Input Gene | 468 |
| --- | --- |
| Diff input count | 468 |
| Diff gene count | 268 |
| No ID Found | 199 |
| Not match annotation count | 200 |
| BP counts | 4001 |
| p-value < 0.05 | 1287 |
| CC counts | 552 |
| p-value < 0.05 | 207 |
| MF counts | 872 |
| p-value < 0.05 | 362 |
| KEGG counts | 195 |
| p-value < 0.05 | 43 |

Summary (Table) information of each query gene/protein: the uniprot record page of each gene/protein is linked with the uniprotID. For BP, CC, MF and KEGG pathway, the complete list of associated terms can be found in the drop down list. Empty cell means lack of annotated information in the database.

**2. Gene Ontology Analysis**

Gene ontology, or GO, is a major bioinformatics initiative to unify the representation of gene and gene product attributes across all species. The project aims to: Maintain and develop its controlled vocabulary of gene and gene product attributes; Annotate genes and gene products, and assimilate and disseminate annotation data; Provide tools for easy access to all aspects of the data provided by the project, and to enable functional interpretation of experimental data using the GO, for example via enrichment analysis. (Wikipedia) The Biological Process, Cell Component and Molecular Function are three basic information of Gene Ontology.

An Enrichment Analysis is to test whether a GO term is statistically enriched for the given set of genes, the hypergeometric test is the most common statistic method of enrichment analysis.
Where N is the number of all genes of the specific organism that were annoted in GO (Background Genes); n is the number of query genes annotated to the GO Term; M is the number of all genes that are annotated to certain GO terms(Pop Hit); m is the number of query genes annotated to certain GO terms(count).


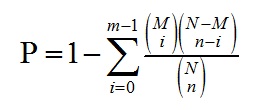


The ontology of GO are structured as a graph, with terms as nodes in the graph and the relations (also known as properties) between the terms as edges. Just as each term is defined, so the relations between GO terms are also categorized and defined. Some of the commonly used relationships in GO: is a (is a subtype of); part of; has part; regulates, negatively regulates and positively regulates. Ontology Relations

Based on the relation, the gene ontology were visualize in a directed acyclic graph (DAG).

Following chart shows an overview of the gene ontology analysis with up to 10 significantly enriched terms in BP, CC, MF categories, respectively. The cut-off of P-value is set to 0.05, Terms of same category are ordered by P-values. Left terms are more significant. Information of percentage and number of involved genes/proteins in a term is shown in left and right y-axis. You can view Biological Process, Cell Component and Molecular Function in corresponding tab.

**2.1 Biological Process Enrichment**

**4812** Biological Process were enriched for this dataset. **1607** are statistically significant with P-value. The following table shows the general information about the enrichment results.

- **Max Level:** maximal annotated level of this term in the GO graph(tree)
- **Levels:** a term can belong to different level in the GO graph. these levels are listed here, separated with ','
- **GO name:** term name
- **GO ID:** starting with GO, followed with a interne number， each GO term has a unique GO ID.
- **P Value:** calculated with Fish exact test with Hyper geometric algorithm.
- **P-value Adjusted**: using 'Benjamin-Hochberg' method for multiple tests
- **Genes:** list of involved genes in the query with this term. Fold change information is listed after the gene name separated with '|', if exists.
- **Count:** Number of genes/proteins in the query that are involved in this term.
- **Pop Hit:** Total number of consistent genes/proteins of this term in database.
- **Background Genes:** Total number of known genes/proteins in a selected species.
- **PAS Value:** The Pathway Activation Strength value, served as the activation profiles of the Signaling pathways based on the expression of individual genes.

Results as shown in file “**RM/Information analysis/GO/BP**”

**2.1 Cell Component Enrichment**

**728** Cell Component were enriched for this dataset. **341** are statistically significant with P-value. The following table shows the general information about the enrichment results.

- **Max Level:** maximal annotated level of this term in the GO graph (tree)
- **Levels:** a term can belong to different level in the GO graph. these levels are listed here, separated with ','
- **GO name:** term name
- **GO ID:** starting with GO, followed with a interge number， each GO term has a unique GO ID.
- **P Value:** calculated with Fish exact test with Hyper geometric algorithm.
- **P-value Adjusted**: using 'Benjamini-Hochberg' method for multiple tests
- **Genes:** list of involved genes in the query with this term. Fold change information is listed after the gene name separated with '|', if exists.
- **Count:** Number of genes/proteins in the query that are involved in this term.
- **Pop Hit:** Total number of consistent genes/proteins of this term in database.
- **Background Genes:** Total number of known genes/proteins in a selected species.

Results as shown in file “**RM/Information analysis/GO/CC**”

**2.1 Molecular Function Enrichment**

**987** Molecular Function were enriched for this dataset. **402** are statistically significant with P-value. The following table shows the general information about the enrichment results.

- **Max Level:** maximal annotated level of this term in the GO graph(tree)
- **Levels:** a term can belong to different level in the GO graph. these levels are listed here, separated with ','
- **GO name:** term name
- **GO ID:** starting with GO, followed with a interge number， each GO term has a unique GO ID.
- **P Value:** calculated with Fish exact test with Hyper geometric algorithm.
- **P-value Adjusted**: using 'Benjamini-Hochberg' method for multiple tests
- **Genes:** list of involved genes in the query with this term. Fold change information is listed after the gene name separated with '|', if exists.
- **Count:** Number of genes/proteins in the query that are involved in this term.
- **Pop Hit:** Total number of consistent genes/proteins of this term in database.
- **Background Genes:** Total number of known genes/proteins in a selected species.

Results as shown in file “**RM/Information analysis/GO/MF**”

## 3. Pathway Analysis

KEGG (Kyoto Encyclopedia of Genes and Genomes) is a manually curate pathway databases. According to KEGG database, Pathways are clustered into the following sub-categories, A) Metabolism, B) Genetic Information Processing, C) Environmental Information Processing, D) Cellular Processes, E) Organismal Systems, F) Human Diseases. Enrichment analysis of KEGG pathways were performed with same hyper geometric algorithm used in gene ontology enrichment analysis.

KEGG pathway was enriched for this dataset. Are statistically significant with . Enriched processes are shown here.

“Figure-Class of enriched KEGG Pathway” in File “**RM/Information analysis/KEGG**”

General information about the enrichment results

File named “Expressed Proteins of KEGG Pathway.txt” and “Enriched KEGG Pathway 2.xl”

- **Pathway Name:** term name
- **Pathway ID:** starting with KEGG, followed with a interge number， each KEGG term has a unique KEGG ID.
- **P Value:** calculated with Fish exact test with Hypergeometric algorithm.
- **P-value Adjusted**: using 'Benjamini-Hochberg' method for multiple tests
- **Genes:** list of involved genes in the query with this term. Fold change information is listed after the gene name separated with '|', if exists.
- **Count:** Number of genes/proteins in the query that are involved in this term.
- **Pop Hit:** Total number of consistent genes/proteins of this term in database.
- **Background Genes:** Total number of known genes/proteins in a selected species.

Top 10 enriched processes are shown here. P value =0.01(red) and P value =0.05(blue) as two selected cutoff are highlighted on the figure, as an indicator to show how significant the results are based on genome background enrichment.

As shown Figure “Distribution of enriched KEGG Pathway”

According to the P-value, each gene/protein in the query is assigned to a most relevant term. The results are shown in following table and pie-chart.

As shown Figure “Expressed Proteins of KEGG Pathway”

## 4. Protein-Protein Interaction Analysis

STRING (Search Tool for the Retrieval of Interacting Genes/Proteins) is a widely used biological database and web resource of known and predicted protein-protein interactions.

The following network model is generated with cytoscape web application, based on information gained up to 4 level of functional analysis: fold change of gene/protein, protein-protein interaction, KEGG pathway enrichment and biological process enrichment. Circle nodes for genes/proteins, rectangle for KEGG pathway or biological process. Pathway was colored with gradient color from yellow to blue, yellow for smaller P-value, and blue for bigger P-value. Biological processes were colored with red. In case of fold change analysis, genes/proteins were colored in red(up-regulation) and green(down-regulation). Default confidence cutoff of 400 was used: interactions with bigger confident score were show as solid lines between genes/proteins, otherwise in dashed lines.

Hints: the network is dynamic, can be zoomed in or out centered at any mouse pointer position. Nodes can be dragged and move to better position. Network can be exported and saved as PNG. Advanced option can be used to develop customized model with selected terms or pathways.

Results as shown in file “RM/Information analysis/PPI”

## 5. Biological Process link KEGG

In most cases, certain biological process and pathway can share same group of genes, implicating the potential inter-linkage between them. The following list shows the detailed linkage information for better understanding of the complexity of the regulation network.

**RM/Information analysis/**
